# Reversible alkene binding and allylic C–H activation with an aluminium(i) complex†
†Electronic supplementary information (ESI) available. Full details of the experimental and computational procedures (PDF), single crystal X-ray data (.cif), coordinates of calculated stationary points (.xyz). CCDC 1870238–1870243. For ESI and crystallographic data in CIF or other electronic format see DOI: 10.1039/c8sc04865g.


**DOI:** 10.1039/c8sc04865g

**Published:** 2019-01-08

**Authors:** Clare Bakewell, Andrew J. P. White, Mark R. Crimmin

**Affiliations:** a Department of Chemistry , Imperial College London , South Kensington , London , SW7 2AZ , UK . Email: m.crimmin@imperial.ac.uk

## Abstract

The monomeric molecular aluminium(i) complex **1** [{(ArNCMe)_2_CH}Al] (Ar = 2,6-di-iso-propylphenyl) reacts with a series of terminal and strained alkenes including ethylene, propylene, allylbenzene and norbornene to form alkene bound products. Remarkably all these reactions are reversible under mild conditions (298–353 K) with alkene binding being disfavoured at higher temperatures due to the positive reaction entropy. Van't Hoff analyses have allowed quantification of the binding events with . Calculations and single crystal X-ray diffraction studies are consistent with the alkene bound species being metallocyclopropane complexes. Alkene binding involves a reversible redox process with changes from the +1 to +3 aluminium oxidation state. Under more forcing conditions the metallocyclopropane complexes undergo non-reversible allylic C–H bond activation to generate aluminium(iii) allyl hydride complexes. This represents a rare example of redox-based main group reactivity in which reversible substrate binding is followed by a further productive bond breaking event. Analysis of the mechanism reveals a reaction network in which alkene dissociation and reformation of **1** is required for allylic C–H activation, a realisation that has important implications for the long-term goal of developing redox-based catalytic cycles with main group compounds.

## Abstract 2

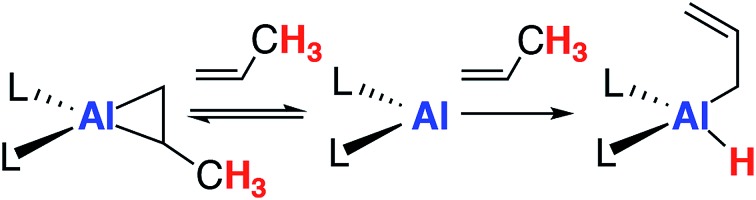
The monomeric molecular aluminium(i) complex **1** [{(ArNCMe)_2_CH}Al] (Ar = 2,6-di-iso-propylphenyl) reacts with a series of terminal and strained alkenes including ethylene, propylene, allylbenzene and norbornene to form alkene bound products.

## Graphical abstract

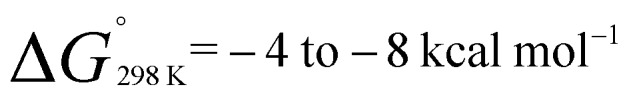

## Introduction

Since the turn of the 21^st^ century, there has been a growing realisation that main group compounds can imitate the behaviour of transition metal complexes.^1^–^4^ The identification of reversible redox processes involving substrate activation is now widely believed to be the most important bottleneck for developing transition-metal-like catalytic cycles. While examples of the oxidative addition of substrates to low-valent main group complexes are rife in the literature, reversible behaviour involving reductive elimination is far less common.^5^–^10^ Due to the paucity of data, it is not yet entirely clear how these reversible redox reactions will be integrated into redox-based catalytic cycles.^11^,^12^

**Fig. 1:**
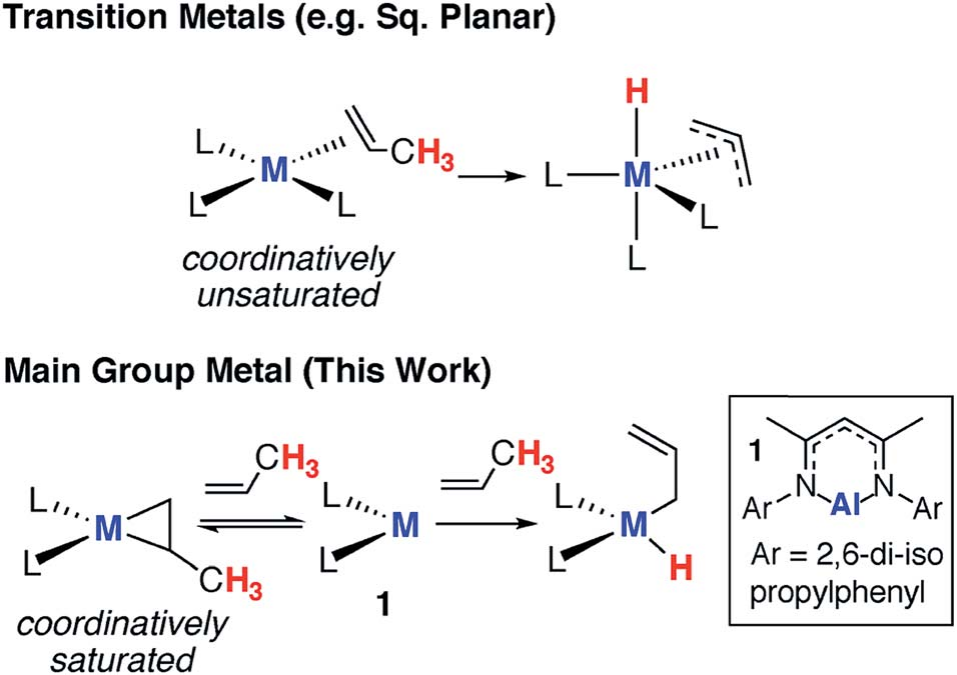
Reversible alkene binding and allylic C–H activation.

Alkenes reversibly bind to countless transition metals and this step has been invoked in numerous aspects of catalysis including hydrogenation, hydroformylation and isomerisation.^13^–^15^ For example, in the π-allyl mechanism for alkene isomerization reversible alkene binding is coupled to the intramolecular activation of an allylic C–H bond, ultimately resulting in the transposition of the unsaturated bond to a new position in the carbon chain.^16^ The key steps in this catalytic pathway have been elucidated for square planar d^8^ iridium complexes (Fig. 1) and related processes are known for d^0^ zirconium complexes.^17^,^18^

In contrast, alkene binding to main group metals is restricted to a few examples. In 2009, following an unusual account of stannylene binding to a strained alkyne,^19^ Power and co-workers documented the first reversible addition of ethylene to a distannyne complex.^20^ Subsequently, the reactions of alkenes with digallynes,^21^ digermynes,^22^–^24^ silylenes,^25^,^26^ low-valent magnesium(i) reagents,^27^ and a diazadiborinine have been reported.^28^ In all but the diazadiborinine system, the alkene scope is limited to ≤2 examples. Furthermore this latter system typically requires temperatures in the range of 383–423 K to effect alkene release.^28^ Hence, close scrutiny of the existing data begs question if these reactions are really a viable first step toward transition metal mimetic main group catalysis or just one-off curiosities.

In this paper, we show that the aluminium(i) reagent **1**, first prepared by Roesky and co-workers,^29^,^30^ reacts reversibly with a range of terminal and strained alkenes (Fig. 1).^31^–^33^ For substrates with allylic C–H bonds, alkene binding is merely a precursor to non-reversible C–H activation. We show that, counter to the mechanism often proposed for transition metal systems, sequential binding and activation events do not involve bond breaking at the metal anchored substrate. Rather dissociation of the alkene and reformation of the aluminium(i) complex is necessary to liberate the reactive site and Frontier molecular orbitals involved in an intermolecular C–H activation step. Our findings not only represent an important advance in transition metal mimetic behaviour of main group complexes, they also demonstrate the complementary mechanistic aspects of these two research fields.

## Results and discussion

### Reversible alkene binding

**Scheme 1:**
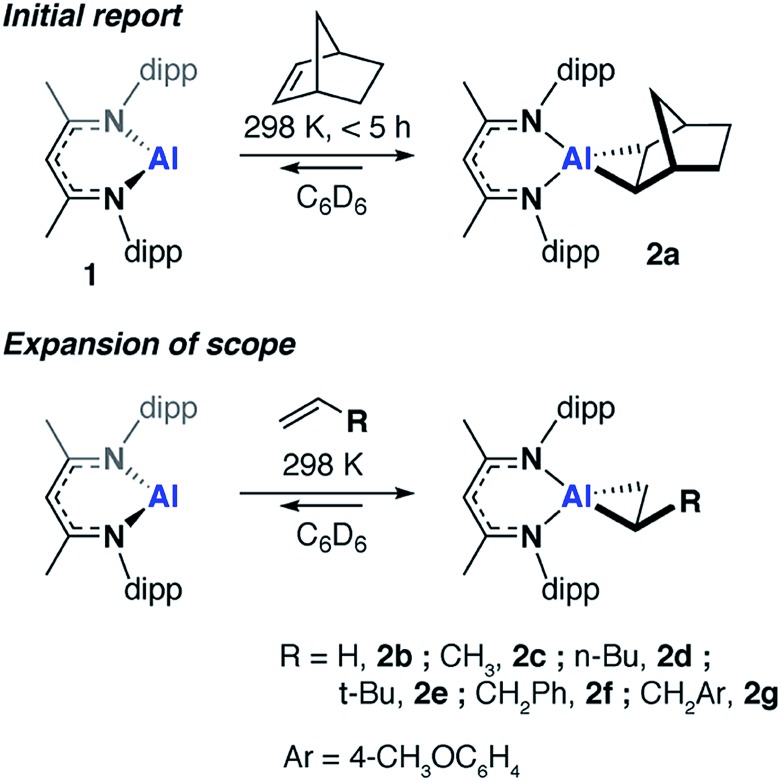
Reversible alkene binding to **1**.

We have previously communicated that the aluminium(i) complex **1**, known to activate a series of small molecules,^34^–^36^ reacts reversibly with norbornene to form the metallocyclopropane complex **2a** (Scheme 1).^37^ A preliminary analysis of the bonding within this complex allowed assignment of **2a** as an genuine aluminium(iii) complex and alkene binding as a redox process involving reversible oxidative addition and reductive elimination steps. In an effort to expand the scope of alkene binding the reaction of **1** with a series of simple and industrially relevant alkenes was investigated (Scheme 1).

The reaction of **1** with an excess of ethylene (1 bar), propylene (1 bar), hex-1-ene, 3,3-dimethyl-1-butene (10 equiv.), allylbenzene (10 equiv.) and 4-allylanisole (10 equiv.) in C_6_D_6_ led to the formation of compounds **2b**, **2c**, **2d**, **2e**, **2f** and **2g** respectively. For example, **2b** formed within 15 minutes at 298 K and the characteristic deep orange colour of **1** in benzene solution was seen to intensify upon alkene binding. In the ^1^H NMR spectrum, a new singlet peak is observed at *δ* = 0.67 ppm corresponding to the four protons of the newly formed and highly symmetric metallocyclopropane moiety of **2b**. In all other cases, the ^1^H NMR data reflect the asymmetric nature of the metallocycle derived from substituted terminal alkenes. In **2f**, magnetically and chemically inequivalent protons of the metallocyclopropane resonate at *δ* = 0.31 (dd, *J* = 12.1 and 8.3 Hz), 0.93 ppm (m) and 1.03 (m) ppm.

The solution behavior of the metallocyclopropane complexes was interrogated by obtaining ^1^H NMR spectroscopy data on isolated samples of **2a–f**, remarkably in all cases alkene binding was found to be reversible. Although the metallocyclopropane is the dominant species in hydrocarbon solutions (benzene, toluene), the position of the equilibrium was found to be dependent on the nature of the alkene. At 298 K, in toluene-d_8_, **2a** equilibrated to <1% of **1** and the non-coordinated alkene, whereas significantly more of **1** was observed to form from **2d** (Table 1). Upon dissolution of single crystals of **2d** in toluene-d_8_ after 298 K for 48 hours **1** and **2d** were present in an 18 : 82 ratio, along with free hex-1-ene. Variable temperature NMR experiments in toluene-d_8_ showed not only that the position of the equilibrium was temperature dependent, but that reaction mixtures were slow to reach equilibrium. For example, after warming to 373 K and cooling back to 298 K samples of **2a** take around 24 hours to re-establish the thermodynamic position of equilibrium (Table 1).

**Table 1:** Equilibrium data for alkene binding to **1**. Determined from [0.018] M, toluene-d_8_ solutions of **2a–f** at 298 K, 1 atm

Alkene (complex)	**1** : **2b–f**
Norbornene (**2a**)	<1 : >99
Ethylene (**2b**)	<1 : >99
Propylene (**2c**)	14 : 86
Hex-1-ene (**2d**)	18 : 82
3,3-Dimethyl-1-butene (**2e**)	10 : 90
Allylbenzene (**2f**)	2 : 98

A van't Hoff analysis of the reaction of norbornene with **1** was conducted in toluene-d_8_ over the temperature range 298–373 K. The formation of **2a** was found to be exergonic; 
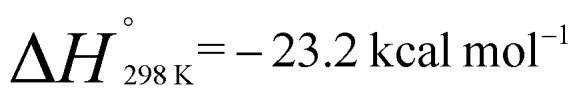
 and 
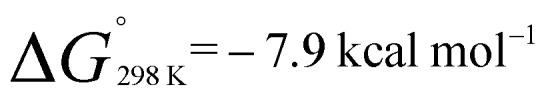
. Similarly, generation of **2d** from **1** and hex-1-ene is a downhill reaction; 
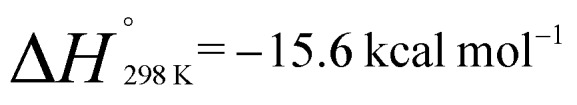
 and 
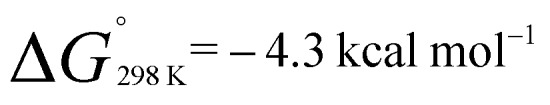
. The more favourable formation of **2a***versus***2d** is in-line with the position of the equilibria at 298 K. It appears that while the position of these equilibria is influenced by both the relief of ring strain and steric factors, the effect of these parameters on the binding energies are only small, leading to reversible behaviour for a broad range of substrates. For comparison, ethylene binding to silylenes and distannynes has been determined to be exothermic with Δ*H*^°^ in the approximate range of –5 to –20 kcal mol^–1^.^20^,^25^,^26^

**Fig. 2:**
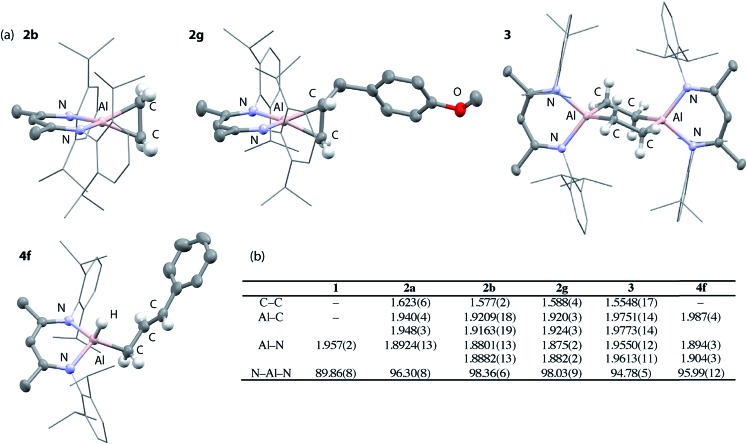
(a) Solid state structures of **2b**, **2g**, **3** and **4f**. (b) Comparison of bond lengths (Å) and angles (^°^).

Compounds **2a-b**, **2d** and **2g** have been further characterized by single crystal X-ray diffraction. Due to the positional disorder of both the metallocyclopropane ring and the *n*-Bu chain of **2d**, the data is not of high enough quality to warrant detailed discussion. High quality data was collected on the remaining members of the series and they all show similar structural parameters for the metallocyclopropane unit. The long C–C bond lengths, short Al–C bond and short Al–N bond lengths (Fig. 2b) are all consistent with the formulation of **2a–g** as metallocyclopropanes in which both the Al and C centers of the three-membered ring are near sp^3^ hybridized. The short Al–N bond lengths can be compared to those in **1**. The ∼0.05 Å difference in these distances is significant as is the change in the N–Al–N bond angle upon alkene binding. Both metrics are consistent with the higher charge density at the Al(iii) center of **2a–g** compared to the Al(i) center of **1**.

Upon ethylene or norbornene binding to **1** there is an 18–21% increase in the C–C bond length compared to the parent alkenes.^38^,^39^ For comparison, transition metal systems typically give rise to far less ‘activated’ bound alkenes. In Zeise's salt, for example, [Pt(Cl)_3_(η^2^-CH_2_CH_2_)][K] the C=C bond of bound ethylene elongates by 3% compared to that in free ethylene.^40^ An 8% elongation is observed in [Ti(Cp*)_2_(η^2^-CH_2_CH_2_)].^41^ The large increases in C–C bond lengths observed with **1** and related main group systems,^20^,^25^,^26^ are typically of complexation of alkenes bearing electron-withdrawing groups (*e.g.* F, Cl, CF_3_, CN) to late transition metals. In these instances, back-donation from metal d-orbitals to the π*-orbital of the alkene is the dominant factor in bonding and alkene coordination can be conceptualised in terms of an oxidative addition to the transition metal.

### Non-reversible metallocycle expansion

**Scheme 2:**
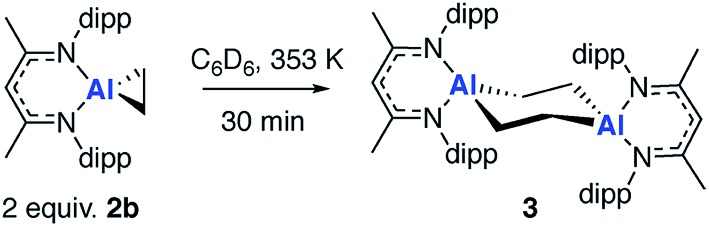
Non-reversible dimerization of **2b** to form **3**.

In the presence of an excess of ethylene, **2b** dimerises to form **3**, a product which incorporates a bimetallocyclohexane ring (Scheme 2). This dimerization occurs over the course of a week at 298 K, but can be accelerated at higher temperatures, taking less than 30 minutes at 353 K. The ^1^H NMR spectrum of **3** shows a highly symmetrical structure, with the methylene protons shifted to *δ* = –0.05 ppm. The structure of **3** was established by single crystal X-ray diffraction (Fig. 2) and DFT calculations (*vide infra*) confirm that **3** is thermodynamically favorable relative to 2 equiv. of **2b** (
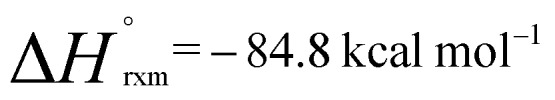
; 
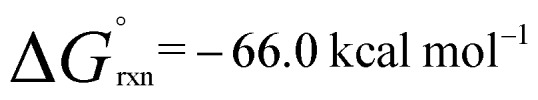
). The formation of **3** is non-reversible. While a series of related compounds were previously reported from the reaction of terminal alkenes with a bimetallic gallium complex, in this instance a metallocyclopropane intermediate could not be observed.^42^ The precise mechanism of the dimerization of **2b** to form **3** remains unclear and although it is tempting to suggest that this involves a simple bimolecular reaction of 2 equiv. of **2b**, at this point the reversible formation of **1** as a reaction intermediate cannot be excluded (*vide infra*).

### Non-reversible allylic C–H activation

**Scheme 3:**
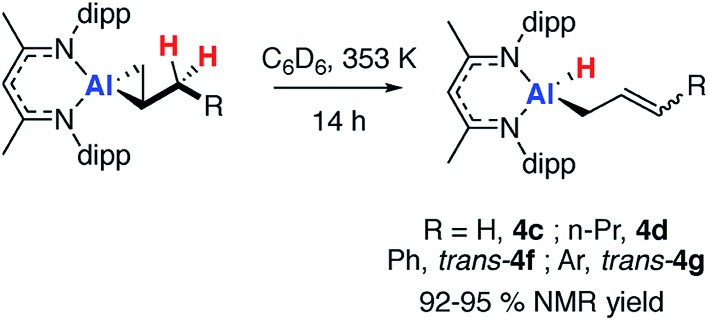
Non-reversible allylic sp^3^ C–H activation of **2c-d** and **2f-g**.

Heating **2c**, in the presence of excess alkene, at 353 K in C_6_D_6_ led to the loss of the dark orange colour and formation of the allylic C–H activation product **4c** (Scheme 3). Allylic C–H activation of propene occurs with migration of the double bond, as confirmed by both NMR spectroscopy and X-ray crystallography. The ^1^H NMR spectrum of **4c** showed three alkene proton resonances at *δ* = 4.38, 4.49 and 5.55 ppm along with the terminal hydride at *δ* ∼ 4.5 ppm.^43^ The X-ray structure showed a four-coordinate Al centre bearing a hydride ligand and an allyl chain which was disordered over two positions (see ESI, Fig. S12†).

Similarly, allylic C–H activation reactions occur upon heating of samples of **2d** and **2f-g** in the presence of an excess of the parent alkene. In these instances, *cis* and *trans* isomers of the products are possible depending on the stereoselectivity of double bond. For **2d** a 1 : 0.6 mixture of isomers of **4d** is formed. Overlapping multinuclear NMR spectra and disordered single crystal data, precluded the assignment of these stereoisomers. C–H bond activation of **2f-g** was more selective and led exclusively to the *trans* isomers *trans*-**4f-g**. The *trans* stereochemistry was confirmed by characteristically large coupling constants (^3^*J*_H–H_ = 15.6 Hz) and single crystal X-ray structure of **4f**, which clearly showed the geometry of the double bond in the chain (Fig. 2a).

Further experiments showed that the formation of these C–H activation products is non-reversible and that accessible allylic sp^3^ C–H bonds are a requirement for further reactivity. Hence, no C–H activation was observed from **2e** under the same conditions and attempts to obtain cross-over products from the reaction of **4f** with alternate alkenes or fluoroalkenes (*e.g.* hexafluoropropene) failed to provide any evidence for the reformation of **1**.

The allylic sp^3^ C–H bonds of propene have a bond dissociation energy of 88.8 ± 0.4 kcal mol^–1^ and while more reactive than those in propane, they are still challenging bonds to break with metal complexes.^44^ The addition of acidic and weak bonds of cyclopentadiene and pentamethycyclopentadiene to [Cp*Al]_4_ and **1** respectively has been reported and in the former case shown to be a reversible redox process.^34^,^45^,^46^ We have also shown that **1** reacts with the C–H bonds of benzene but only in the presence of a palladium catalyst.^43^ An anionic aluminyl complex can also effect C–H activation of benzene in the absence of catalyst.^47^

### Kinetics

Kinetic data was obtained and modelled with Copasi software. These experiments were undertaken as a means to gain insight in to the reaction mechanism and establish the activation parameters for the C–H activation step. A 0.018 M solution of **1** in benzene-d_6_ was reacted with a 5 equiv. of allylbenzene, with full formation of **2f** observed after 1 hour. The solution was heated to 343 K and monitored by ^1^H NMR spectroscopy over the course of 4 hours. Immediate formation of **4f** was observed, along with small amounts of unreacted **1**. Complex **1** remained present in low, but steady, concentration (<5%) throughout the reaction, suggesting that it may be a potential intermediate in C–H activation.

**Fig. 3:**
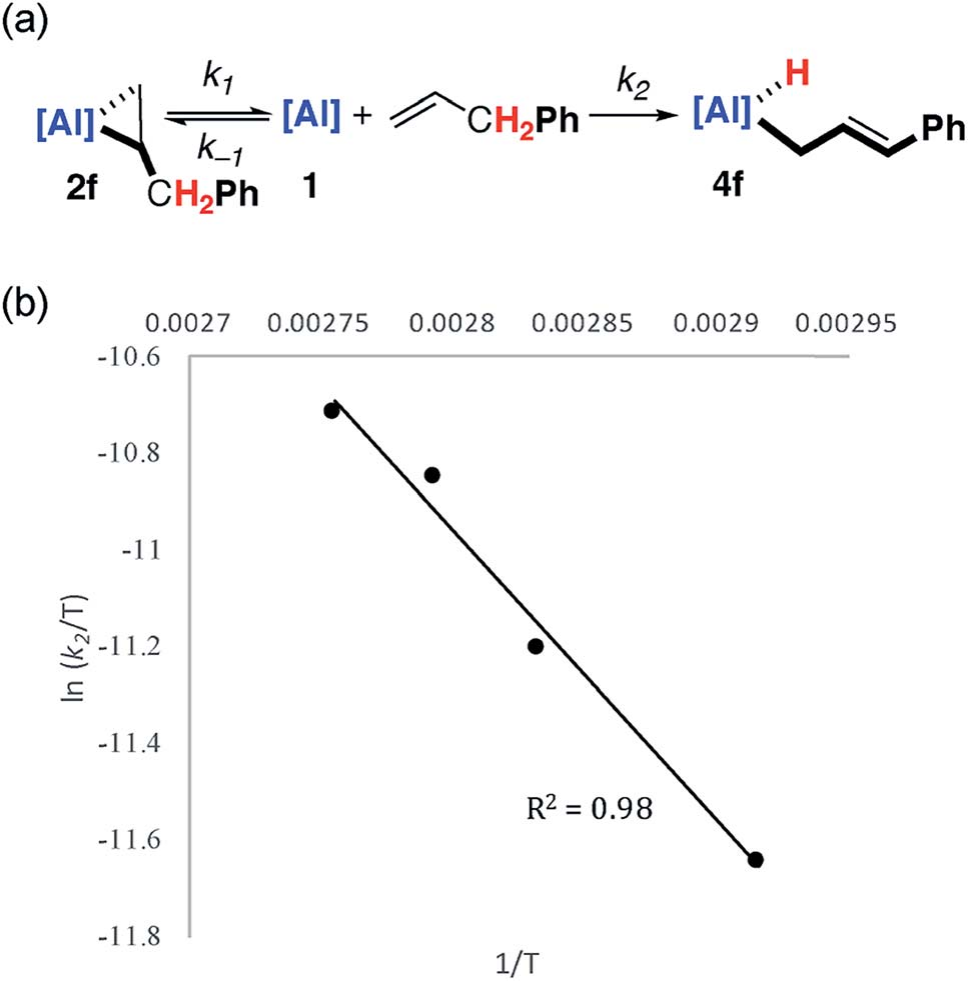
(a) Modelled (Copasi) reaction network for the reaction of **1** with allyl benzene. (b) Eyring analysis plot of ln(*k*_2_/*T*) *versus* 1/*T*.

Attempts to fit the data using pseudo-first order kinetics as a conversion from **2f** to **4f** did not lead to reasonable activation parameters. The system was considered as an equilibrium between **1** and **2f**, with non-reversible conversion of **1** to **4f** (Fig. 3a). Copasi software was used to model the reaction network, fit the kinetic data, and extract rate constants for both the equilibrium (*k*_–1_/*k*_1_ = 0.056) and the irreversible C–H activation (*k*_2_ = 3.0 × 10^–3^ s^–1^) at 343 K. An alternate kinetic model exists. The reaction network could also be considered as an equilibrium between **1** and **2f** with non-reversible conversion of **2f** to **4f**. While the two kinetic scenarios involving a pre-equilibrium step cannot be differentiated from one another experimentally, calculations provide unambiguous support for the involvement of **1** as an intermediate and show that the direct conversion of **2f** to **4f** is in fact unfavourable (*vide infra*).

Eyring analysis of the C–H activation reaction over the temperature range 343–363 K (**1** to **4f**, *k*_2_) yielded the activation parameters (Δ*H*^‡^ = 10.0 kcal mol^–1^) and (Δ*S*^‡^ = –35.5 cal K^–1^ mol^–1^), with a Gibbs activation energy of Δ*G*‡298 K = 20.5 kcal mol^–1^ (Fig. 3b). The magnitude and sign of the activation entropy is consistent with the formation of an ordered transition state and a bimolecular reaction. The Gibbs activation energy is in modest agreement with the computationally derived activation parameter Δ*G*‡298 K = 17.6 kcal mol^–1^ for the associated non-reversible allylic C–H activation step (*vide infra*).

### DFT calculations

To gain a better understanding the electronic structures of **2a-g**, the nature of the reversible binding event and the mechanism of C–H activation a series of calculations were conducted. Density functional theory (DFT) calculations were conducted using the hybrid basis set 6-31G**/SDD. A series of functionals were investigated and the M06L functional was found to best model the structural and thermodynamic parameters determined from experiment (see ESI†) whilst also capturing the observed reaction trends. Solvent and dispersion effects were considered using single point corrections on the optimised geometries. Reversible ethylene binding to a simplified model of **1** was predicted computationally prior to its experimental isolation.^48^

**Fig. 4:**
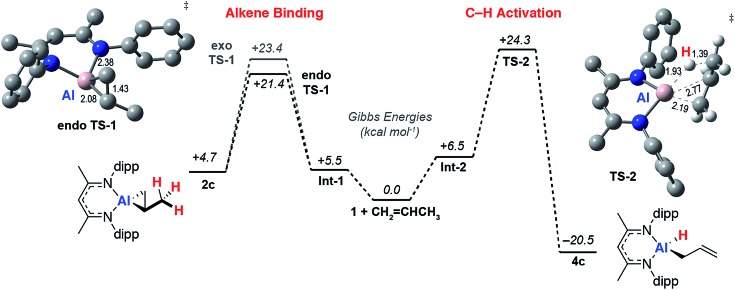
Calculated reaction pathway for reversible alkene binding and allylic C–H activation with **1**. Annotated with representations of **endo-TS-1** and **TS-2** with selected bond lengths (Å). M06L functional and hybrid (Al, SDDAll; 6-31G**, C, H, N) basis set + Δ*E*_solv_ (PCM, benzene).

As the simplest substrate to undergo both binding and C–H activation, propylene was made the focus of these studies. Alkene binding was determined to take place through an asynchronous concerted pathway involving two closely related transition states, **endo-TS-1** and **exo-TS-1** with Δ*G*^‡^ = 21.4 and 23.4 kcal mol^–1^ respectively. These transition states differ in the orientation of the alkene and both evolve from a weakly bound encounter complex **Int-1** (Fig. 4). NBO analysis of the stationary points confirms the assignment of alkene binding as a redox process. The NPA charge on Al increases as the forward reaction progresses consistent with an increase in oxidation state from +1 to +3 (**1**, 0.78; **Int-1**, 0.79; **endo-TS-1**, 1.32; **2c**, 1.83). At the same time the C=C Wiberg bond indices decrease (**Int-1**, 1.40; **endo-TS-1**, 1.14; **2c**, 1.01), while those of the Al–C bonds increase (**endo-TS-1**, 0.75 and 0.72; **2c**, 0.77 and 0.73). **Endo-TS-1** is asymmetric being characterised by not only the displacement of the Al atom out of the plane of the β-diketiminate ligand but also two distinct Al···C distances which differ by ∼0.3 Å (Fig. 3). Similar transition states were calculated for the whole series of alkenes with a range of activation energies of Δ*G*^‡^ = 18–21 kcal mol^–1^ (ESI, Tables S2 and S3†). A structurally related TS has been calculated for the addition of H_2_ to **1**.^49^,^50^ The geometry can be explained by considering the oxidative addition transition state in terms of a donation of electron density in the C=C π-bond to the vacant p-orbital on Al with concomitant back-donation from the Al sp^2^ lone pair to the π* orbital of the alkene (*vide infra*).

A low energy activation pathway was identified involving intermolecular oxidative addition of the C–H bond of propylene to **1**. This pathway proceeds by **TS-2** with an activation barrier of Δ*G*^‡^ = 24.3 kcal mol^–1^. The energies of **TS-1** and **TS-2** are consistent with the metallocyclopropane **2c** being formed as the kinetic product which ultimately converts to the thermodynamic product **4c***via* reformation of **1**. Formation of **4c** is non-reversible and proceeds in an exergonic step, 
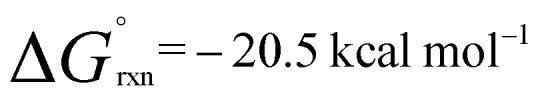
. In **TS-2**, propylene develops the character of an allylic ligand as it undergoes C–H activation. The Al···H bond length (1.93 Å) and C···H bond length (1.39 Å) are consistent with a late transition state. The allylic character is evidenced by a the relatively short interaction between Al and the terminal alkene carbon of the propylene moiety (2.19 Å). The NPA charge of Al increases as the C–H bond breaks, consistent with an oxidative addition (**1**, 0.78; **Int-2**, +0.79; **TS-2**, +1.42; **4c**, +1.76). IRC calculations confirm that **TS-2** does not evolve from **2c**, but instead is the result of C–H activation directly from **1** and free propylene.

A pathway for the direct intramolecular C–H activation of the bound propylene of the aluminium(iii) complex **2c** could not be identified at this level of theory. This latter reaction pathway involves a β-hydride elimination step known to operate for simpler three coordinate aluminium(iii) alkyls at high temperatures,^51^ but apparently disfavoured within this strained metallocyclic system. With an alternate computational approach the transition state for the direct conversion of **2** → **4** could be identified and compared with that of **1** → **4.** With the ωB97x functional, a hybrid basis-set (Al, SDDAll; 6-31G**, C, H, N) adapted for solvent (PCM, benzene) and dispersion (ωB97xD) by single point corrections, the transition state for β-hydride elimination of **2f** was located and found to be obstructively high in energy >40 kcal mol^–1^. For comparison at the same level of theory **TS-2** is Δ*G*^‡^ = 27.1 kcal mol^–1^. Related β-fluoride elimination pathways have been modelled in these systems and are lower in energy likely due to a large (and non-directional) ionic component to Al–F bonding and the fluorophilicity of the Al^3+^ ion.^37^

An analogous reaction profile to that presented in Fig. 4 was identified for allylbenzene (Fig. S20†). Experimentally *trans*-**4f** was observed as the sole reaction product over a 343–363 K temperature range. Comparison of the C–H activation transition states explains the regioselectivity. The Gibbs free energy (at 298 K) for the transition state for the formation of *trans*-**4f** was ∼3 kcal mol^–1^ lower in energy than the TS that leads to *cis*-**4f**, likely due to 1,3-allylic strain (A-strain) induced as the hydrogen atom is transferred to aluminium and the hydrocarbon ligand starts to adopt alkene character (Fig. S18†). The calculations support the experimental observation that only *trans*-**4f** is formed.

### MO analysis

**Fig. 5:**
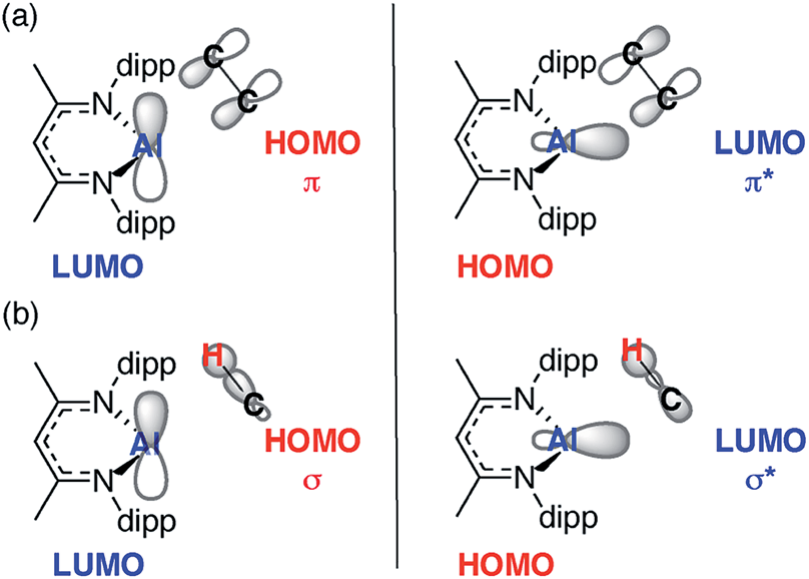
Simplified MO analysis of (a) **TS-1** and (b) **TS-2**.

In combination the DFT calculations and kinetics support the presence of two competitive pathways: (i) reversible alkene binding to form a metallocyclopropane and (ii) direct intermolecular C–H activation, *via* an oxidative addition, leading to C–H activation of the alkene. A simple MO analysis of **1** yields an intuitive understanding of the results presented herein. The Frontier molecular orbitals (fMOs) of **1** consist of a vacant 3p orbital (LUMO) and an orthogonal sp^2^-hybridised lone-pair (HOMO). Alkene binding proceeds *via* an asymmetric transition state that involves overlap of the fMOs of **1** with those of the unsaturated C=C bond (Fig. 5a). The resultant alkene complexes **2a–g** are coordinatively saturated with pseudo-tetrahedral geometries at aluminium and no low-lying molecular orbitals that can participate in facile reactions. As such intramolecular pathways for activation of the bound alkene such as β-hydride elimination, prolific for transition-metal counterparts of **1** with additional low-lying empty orbitals, are disfavoured. While sequential alkene binding and C–H activation can be observed with **2c** this requires dissociation of the alkene to liberate the coordinatively unsaturated intermediate **1**. C–H activation, like alkene binding, involves overlap of the fMOs of **1** with those the C–H σ-bond (Fig. 5b).

## Conclusions

In summary, we report the first examples of reversible alkene binding to an aluminium(i) centre, along with an extremely rare case of a reaction sequence involving reversible substrate binding and C–H activation at a main group fragment. The simple realisation that the fMOs of aluminium(i) required for alkene binding are also those required for C–H activation has broad implications for the development of catalytic cycles.

Low-valent main group species with small HOMO–LUMO gaps are often targeted as a first step to develop redox catalysis, but the reality is that the most common designs (*e.g.* two coordinate borylenes, silylenes, stannylenes or three coordinate phosphorus compounds with constrained geometries) only allow for binding or activation of a single substrate at a time. In contrast, for transition metal catalysts often several d-orbitals of suitable energy and symmetry are unoccupied. Most transition metal based redox catalytic cycles involve the coordination or activation of two substrates (or two functional groups within the same substrate), bringing them into close proximity and facilitating bond formation.

Our current approach with the main group may only be the first step toward design of redox active catalysts. Once reversible substrate binding has been achieved there needs to be a considered understanding of the subsequent possible steps. Future work must address bond formation following reversible substrate binding and there are opportunities to explore new approaches such as the use of: (i) hemi-labile ligands to open up coordination sites on the main group fragment, or (ii) integration of redox steps with more common pathways of main group compounds such as hydroelementation, σ-bond metathesis and nucleophilic addition.

## Author contributions

The manuscript was written through contributions of all authors. All authors have given approval to the final version of the manuscript.

## Conflicts of interest

There are no conflicts to declare.

## Supplementary Material

Supplementary information

Crystal structure data
