# Supplementary material for: Reversible alkene binding and allylic C–H activation with an aluminium(i) complex
Source: Chem Sci. 2019 Jan 8;10(8):2452–8. doi: 10.1039/c8sc04865g (PMC6388093; doi:10.1039/c8sc04865g)
Supplement: Supplementary file 1 [file SC-010-C8SC04865G-s001.pdf]

Electronic Supplementary Material (ESI) for Chemical Science.
This journal is © The Royal Society of Chemistry 2019

Reversible Alkene Binding and Allylic sp^3 C–H Activation with an Aluminum(I) Complex

Clare Bakewell, Andrew J. P. White and Mark R. Crimmin*

Department of Chemistry, Imperial College London, South Kensington, London, SW7 2AZ, UK.

Table of Contents

**1**	**General Experimental Section**	**S3**
**2**	**Synthetic Procedures**	**S4**
**3**	**Reversible Alkene Binding**	**S9**
**4**	**Kinetic Analysis**	**S13**
**5**	**X-ray Data**	**S18**
**6**	**Computational Details**	**S21**
**7**	**Multinuclear NMR Daya**	**S27**
**8**	**References**	**S36**

1. General Experimental Section

All manipulations were carried out using standard Schlenk-line and glovebox techniques under an inert atmosphere of argon or dinitrogen. A MBraun Labmaster glovebox was employed, operating at < 0.1 ppm O_2 and < 0.1 ppm H_2O. Solvents were dried over activated alumina from an SPS (solvent purification system) based upon the Grubbs design and degassed before use. Glassware was dried for 12 h at 120 °C prior to use. Benzene-d_6 was stored over 3Å molecular sieves and distilled prior to use. NMR-scale reactions were conducted in J. Young's tap tubes and prepared in a glovebox. All heating mentioned was done using silicone oil baths. ^{1}H (tetramethylsilane; 0 ppm) and ^{13}C (tetramethylsilane; 0 ppm) spectra were obtained on BRUKER 400 MHz or 500 MHz machines unless otherwise stated; all peak intensities are derived from internal standard peaks with values quoted in ppm. Data was processed using the MestReNova or Topsin software. C^{IV} refers to quaternary carbons.

CHN analysis were not possible on the metallocyclopropane complexes **2b- g** due to their potential to liberate the alkene under thermal conditions. Due to this thermal instability multinuclear NMR data were collected at either 273 or 298 K.

Propylene and ethylene were purchased from BOC, and dried and deoxygenated using a CRS Model 500 Molecular Sieve Drying Purifier and a CRS Model 1000 Oxygen trap, respectively. All other alkenes were purchased from Sigma Aldrich or Alfa Aeser and distilled and stored over 3Å molecular sieves prior to use. Other chemicals were purchased from Sigma Aldrich, Fluorochem or Alfa Aeser. Complex **1** was prepared by the literature procedure.[1]

2. Synthetic Procedures

2.1 General procedures for the reaction with gases

NMR Scale Reactions: Complex **1** (5 mg, 0.01 mmol) was dissolved in benzene-d_6 (0.6 mL) and the solution transferred into a Young's tap NMR tube equipped with a capillary tube containing a ferrocene standard solution; a t=0 ^{1}H NMR spectrum was recorded. The sample was then degassed (freeze/pump/thaw) three times, and the gas (ethylene or propylene) was added to the tube at approximately 1 bar pressure. The reaction was monitored by ^{1}H NMR spectroscopy. NMR yields were recorded by comparison against the ferrocene internal standard, δ = 4.00 ppm.

Preparative Scale Reactions: Complex **1** (50 mg, 0.1 mmol) was dissolved in benzene-d_6 (1 mL) and the solution transferred into a Young's tap NMR tube. The sample was then degassed (freeze/pump/thaw) three times, and the gas (ethylene or propylene) was added to the tube at approximately 1 bar pressure. The reaction was monitored by ^{1}H NMR spectroscopy. Following the end of the reaction the solvent was removed *in vacuo* and the products were recrystallized from concentrated *n*-hexane at –35 °C.

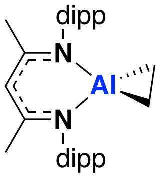

Ethylene: Reaction complete after 15 minutes; 98 % yield versus ferrocene. X-ray quality crystals were isolated from concentrated *n*-hexane at –35 °C. **2b:** ^{1}H NMR (400 MHz, benzene-d_6, 298 K): 0.67 (s, 4H, CH_2CH_2), 1.09 (d, 12H, CH(CH_3)$_2$, $^3J_{HH}$ = 6.8 Hz), 1.41 (d, 12H, CH(CH_3)$_2$, $^3J_{HH}$ = 6.8 Hz), 1.55 (s, 6H, CH_3), 3.47 (sept, 4H, CH(CH$_3$)$_2$, $^3J_{HH}$ = 6.8 Hz), 4.84 (s, 1H, C(CH$_3$)CHC(CH$_3$)), 7.07-7.14 (m, 6H, ArH); ^{13}C{^{1}H} NMR (100 MHz, benzene-d_6, 298 K): 3.8 (CH$_2$$CH_2$), ($CH_3$), 24.2 (CH($CH_3$)$_2$), 24.4 (CH($CH_3$)$_2$), 28.5 ($C$H(CH$_3$)$_2$), 95.7 (C(CH$_3$)$C$HC(CH$_3$)), 124.3 ($C$H), 127.6 ($C$H), 138.1 (C^{IV}), 144.0 (C^{IV}), 172.5 (C(CH$_3$)CHC(CH$_3$)).

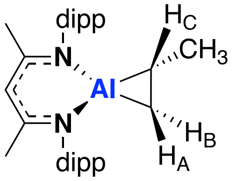

Propylene: Reaction complete after 1 hour; 88 % yield versus ferrocene. **2c:** ^{1}H NMR (400 MHz, toluene-d_8, 273 K): 0.16 (dd, 1H, CH^B, $^2J_{HH}$ = 9.7 Hz, $^3J_{HH}$ = 7.2 Hz), 0.48 (m, 1H, CH^CCH$_3$), 0.97 (d, 3H, CH(CH_3)$_2$, $^3J_{HH}$ = 6.8 Hz), 0.99 (d, 3H, CH(CH_3)$_2$, $^3J_{HH}$ = 6.8 Hz), 1.04 (d, 3H, CH^{B}CH_3, $^3J_{HH}$ = 6.2 Hz), 1.08 (d, 6H, CH(CH_3)$_2$, $^3J_{HH}$ = 6.8 Hz), 1.25 (dd, 1H, CH^B, $^2J_{HH}$ = 9.7 Hz, $^3J_{HH}$ = 9.7 Hz), 1.30 (d, 3H, CH(CH_3)$_2$, $^3J_{HH}$ = 6.8 Hz), 1.33 (d, 3H, CH(CH_3)$_2$, $^3J_{HH}$ = 6.8 Hz), 1.35 (d, 3H, CH(CH_3)$_2$, $^3J_{HH}$ = 6.8 Hz), 1.40 (d, 3H, CH(CH_3)$_2$, $^3J_{HH}$ = 6.8 Hz), 1.46 (s, 3H, CH_3), 1.48 (s, 3H, CH_3), 3.34 (m, 3H, CH(CH$_3$)$_2$), 3.45 (sept, 1H, CH(CH$_3$)$_2$, $^3J_{HH}$ = 6.8 Hz), 4.73 (s, 1H, C(CH$_3$)CHC(CH$_3$)), 6.90–7.10 (m, 6H, ArH); ^{13}C{^{1}H} NMR (100 MHz, benzene-d_6, 298 K): 15.1 (AlC(H^C)(CH$_3$)), 17.8 (AlCH^{A}H^B), 22.2 (AlC(H^A)(CH$_3$)), 23.0 (CH$_3$), 23.1 (CH$_3$), 24.1 (CH(CH$_3$)$_2$), 24.3 (3 x CH(CH$_3$)$_2$), 24.6 (CH(CH$_3$)$_2$), 24.7 (CH(CH$_3$)$_2$), 24.8 (CH(CH$_3$)$_2$), 25.0 (CH(CH$_3$)$_2$), 28.5 (CH(CH$_3$)$_2$), 28.5 (CH(CH$_3$)$_2$), 29.0 (CH(CH$_3$)$_2$), 29.1 (CH(CH$_3$)$_2$), 96.0 (C(CH$_3$)CHC(CH$_3$)), 124.4 (CH), 124.6 (CH), 124.8 (CH), 127.8 (CH), 127.8 (CH), 138.4 (C^{IV}), 138.7 (C^{IV}), 143.4 (C^{IV}), 143.7 (C^{IV}), 144.4 (C^{IV}), 144.5 (C^{IV}), 172.4 (C(CH$_3$)CHC(CH$_3$)), 172.4 (C(CH$_3$)CHC(CH$_3$)).

2.2 General procedures for the reactions with liquids and solids

NMR Scale Reactions: In a glovebox, complex **1** (5 mg, 0.01 mmol) was dissolved in benzene-d_6 (0.6 mL) and the solution transferred into a Young's tap NMR tube equipped with a capillary tube containing a ferrocene standard solution; a t=0 ^{1}H NMR spectrum was recorded. The reaction was monitored by ^{1}H NMR spectroscopy. NMR yields were recorded by comparison against the ferrocene internal standard, δ = 4.00 ppm.

Preparative Scale Reactions: Complex **1** (50 mg, 0.1 mmol) was dissolved in benzene-d_6 (1 mL), followed by ten equivalents of the alkene (0.1 mmol) the reaction mixture was transferred to a J Young's tap NMR tube and removed from the box. Following the end of the reaction the solvent was removed *in vacuo* and the products were recrystallized from concentrated *n*-hexane at –35 °C, unless stated otherwise.

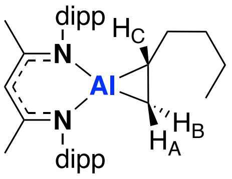

Hex-1-ene: Reaction complete after 15 mins; 98 % yield versus ferrocene. X-ray quality crystals were isolated from a concentrated solution *n*-hexane/toluene at –35 °C. **2d:** ^{1}H NMR (400 MHz, toluene-d_8, 298 K): 0.16 (dd, 1H, CH^B, $^2J_{HH}$ = 12.1 Hz, $^3J_{HH}$ = 9.6 Hz), 0.48 (m, 1H, CH^C), 0.82 (t, 3H, hexyl-CH_3, $^3J_{HH}$ = 7.2 Hz), 1.05 (d, 3H, CH(CH_3)$_2$, $^3J_{HH}$ = 6.8 Hz), 1.07 (d, 3H, CH(CH_3)$_2$, $^3J_{HH}$ = 6.8 Hz), 1.10 (1H, CH^A, under doublets), 1.13 (d, 3H, CH(CH_3)$_2$, $^3J_{HH}$ = 6.8 Hz), 1.14 (d, 3H, CH(CH_3)$_2$, $^3J_{HH}$ = 6.8 Hz), 1.36 (d, 3H, CH(CH_3)$_2$, $^3J_{HH}$ = 6.8 Hz), 1.39 (d, 3H, CH(CH_3)$_2$, $^3J_{HH}$ = 6.8 Hz), 1.42 (d, 3H, CH(CH_3)$_2$, $^3J_{HH}$ = 6.8 Hz), 1.45 (d, 3H, CH(CH_3)$_2$, $^3J_{HH}$ = 6.8 Hz), 1.54 (s, 3H, CH_3), 1.56 (s, 3H, CH_3), 3.44 (m, 4H, CH(CH$_3$)$_2$), 4.82 (s, 1H, C(CH$_3$)CHC(CH$_3$)), 6.95–7.15 (m, 6H, ArH), 3 x CH_2 groups broad multiplets between 0.80 and 1.50 ppm; ^{13}C{^{1}H} NMR (100 MHz, toluene-d_8, 273 K): 14.5 (AlCH$_A$H$_B$), 14.7 (hexyl-CH$_3$), 21.3 (AlCH$_C$), 23.1 (2 x CH$_3$), 23.2 (CH$_2$), 24.2 (2 x CH(CH$_3$)$_2$), 24.4 (2 x CH(CH$_3$)$_2$), 24.7 (CH(CH$_3$)$_2$), 24.7 (CH(CH$_3$)$_2$), 24.7 (CH(CH$_3$)$_2$), 25.0 (CH(CH$_3$)$_2$), 28.5 (2 x CH(CH$_3$)$_2$), 29.0 (CH(CH$_3$)$_2$), 29.1 (CH(CH$_3$)$_2$), 32.9 (CH$_2$), 35.6 (CH$_2$), 96.1 (C(CH$_3$)CHC(CH$_3$)), 124.3 (CH), 124. 4 (CH), 124.7 (CH), 124.8 (CH), 127.8 (CH), 127.8 (CH), 138.5 (C^{IV}), 139.0 (C^{IV}), 143.4 (C^{IV}), 142.7 (C^{IV}), 144.3 (C^{IV}), 144.6 (C^{IV}), 172.4 (C(CH$_3$)CHC(CH$_3$)), 172.4 (C(CH$_3$)CHC(CH$_3$)).

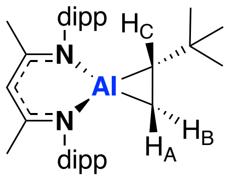

3,3-Dimethyl-1-butene: Reaction complete after 15 mins; 98 % yield versus ferrocene. X-ray quality crystals were isolated from a concentrated solution *n*-hexane/toluene at –35 °C. **2e:** ^{1}H NMR (400 MHz, toluene-d_8, 273 K): 0.46 (dd, 1H, CH^C, $^3J_{HH}$ = 13.8 Hz, $^3J_{HH}$ = 11.8 Hz), 0.60 (dd, 1H, C$H^{A/B}$, $^2J_{HH}$ = 13.8 Hz, $^3J_{HH}$ = 11.8 Hz), 0.77 (s, 9H, *t*Bu), 1.00 (d, 3H, CH(CH_3), $^3J_{HH}$ = 6.8 Hz), 1.03 (d, 3H, CH(CH_3), $^3J_{HH}$ = 6.8 Hz), 1.12 (d, 3H, CH(CH_3), $^3J_{HH}$ = 6.8 Hz), 1.14 (d, 3H, CH(CH_3), $^3J_{HH}$ = 6.8 Hz), 1.15 (m, 1H, C$H^{A/B}$), 1.36 (d, 3H, CH(CH_3), $^3J_{HH}$ = 6.8 Hz), 1.41 (d, 3H, CH(CH_3), $^3J_{HH}$ = 6.8 Hz), 1.48 (d, 3H, CH(CH_3), $^3J_{HH}$ = 6.8 Hz), 1.49 (s, 3H, CH_3), 1.49 (d, 3H, CH(CH_3), $^3J_{HH}$ = 6.8 Hz), 1.51 (s, 3H, CH_3), 3.37 (sept, 1H, CH(CH$_3$)$_2$, $^3J_{HH}$ = 6.8 Hz), 3.47 (sept, 1H, CH(CH$_3$)$_2$, $^3J_{HH}$ = 6.8 Hz), 3.54 (m, 2H, CH(CH$_3$)$_2$), 4.83 (s, 1H, C(CH$_3$)CHC(CH$_3$)), 7.00-7.15 (m, 6H, ArH); ^{13}C{^{1}H} NMR (100 MHz, toluene-d_8, 273 K): 10.2 (CH^{A}H^B), 23.4 (CH$_3$), 23.5 (CH$_3$), 24.1 (CH(CH$_3$)$_2$), 24.3 (CH(CH$_3$)$_2$), 24.4 (CH(CH$_3$)$_2$), 24.6 (CH(CH$_3$)$_2$), 24.6 (CH(CH$_3$)$_2$), 24.7 (CH(CH$_3$)$_2$), 24.9 (CH(CH$_3$)$_2$), 25.2 (CH(CH$_3$)$_2$), 28.2 (2 x

*C*H(CH$_3$)$_2$), 29.1 (*C*H(CH$_3$)$_2$), 29.2 (*C*H(CH$_3$)$_2$), 31.0 (C(*C*H$_3$)$_3$), 33.5 (*C*(CH$_3$)$_3$), 38.4 (*C*H^C), 96.7 (C(CH$_3$)C*H*C(CH$_3$)), 124.1 (*C*H), 124.4 (0, 124.8 (*C*H), 125.0 (*C*H), 127.8 (*C*H), 127.8 (*C*H), 139.4 (C^{IV}), 139.9 (C^{IV}), 142.8 (C^{IV}), 143.1 (C^{IV}), 144.6 (C^{IV}), 154.0 (C^{IV}), 172.4 (*C*(CH$_3$)CHC(CH$_3$)), 172.8 (C(CH$_3$)CH*C*(CH$_3$)).

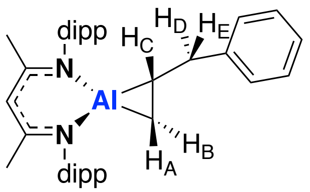

Allylbenzene: Reaction complete after 1 hour; 83 % yield versus ferrocene. X-ray quality crystals were isolated from concentrated *n*-hexane at –35 °C. **2f:** ^{1}H NMR (400 MHz, benzene-d_6, 298 K): 0.31 (dd, 1H, C*H*^{B}, $^2J_{HH}$ = 11.5 Hz, $^3J_{HH}$ = 7.8 Hz), 0.94 (m, 1H, C*H*^{C}), 1.01 (d, 3H, CH(C*H*$_3$), $^3J_{HH}$ = 6.8 Hz), 1.02 (d, 3H, CH(C*H*$_3$), $^3J_{HH}$ = 6.8 Hz), 1.03 (1H, C*H*^{A}, under doublets), 1.14 (d, 3H, CH(C*H*$_3$), $^3J_{HH}$ = 6.8 Hz), 1.15 (d, 3H, CH(C*H*$_3$), $^3J_{HH}$ = 6.8 Hz), 1.26 (d, 3H, CH(C*H*$_3$), $^3J_{HH}$ = 6.8 Hz), 1.40 (d, 3H, CH(C*H*$_3$), $^3J_{HH}$ = 6.8 Hz), 1.43 (d, 3H, CH(C*H*$_3$), $^3J_{HH}$ = 6.8 Hz), 1.46 (d, 3H, CH(C*H*$_3$), $^3J_{HH}$ = 6.8 Hz), 1.51 (s, 6H, C*H*$_3$), 1.56 (s, 6H, C*H*$_3$), 1.84 (dd, 1H, C*H*^{D} or C*H*^{E}, $^2J_{HH}$ = 14.4 Hz, $^3J_{HH}$ = 12.1 Hz), 2.93 (dd, 1H, C*H*^{D} or C*H*^{E}, $^2J_{HH}$ = 14.4 Hz, $^3J_{HH}$ = 4.2 Hz), 3.38 (sept, 2H, C*H*(CH$_3$)$_2$, $^3J_{HH}$ = 6.8 Hz), 3.48 (sept, 1H, C*H*(CH$_3$)$_2$, $^3J_{HH}$ = 6.8 Hz), 3.53 (sept, 1H, C*H*(CH$_3$)$_2$, $^3J_{HH}$ = 6.8 Hz), 4.83 (s, 1H, C(CH$_3$)C*H*C(CH$_3$)), 6.94-7.20 (m, 11H, Ar*H*); ^{13}C{^{1}H} NMR (100 MHz, benzene-d_6, 298 K): 13.7 (Al*C*H$_A$H$_B$) 21.4 (Al*C*H$_C$), 22.8 (*C*H$_3$), 22.8 (*C*H$_3$), 23.9 (CH(*C*H$_3$)$_2$), 24.0 (CH(*C*H$_3$)$_2$), 24.0 (CH(*C*H$_3$)$_2$), 24.0 (CH(*C*H$_3$)$_2$), 24.3 (2 x CH(*C*H$_3$)$_2$), 24.4 (CH(*C*H$_3$)$_2$), 24.8 (CH(*C*H$_3$)$_2$), 28.2 (*C*H(CH$_3$)$_2$), 28.3 (*C*H(CH$_3$)$_2$), 28.8 (2 x *C*H(CH$_3$)$_2$), 41.0 (*C*H$_D$H$_E$), 95.9 (C(CH$_3$)C*H*C(CH$_3$)), 124.1 (*C*H), 124.2 (*C*H), 124.3 (*C*H), 124.5 (*C*H), 124.7 (*C*H), 127.4 (*C*H), 127.6 (*C*H), 128.5 (*C*H), 138.1 (C^{IV}), 138.7 (C^{IV}), 143.4 (C^{IV}), 143.5 (C^{IV}), 144.2 (C^{IV}), 144.5 (C^{IV}), 146.5 (C^{IV}), 172.3 (C(CH$_3$)CH*C*(CH$_3$)), 172.3 (*C*(CH$_3$)CHC(CH$_3$)).

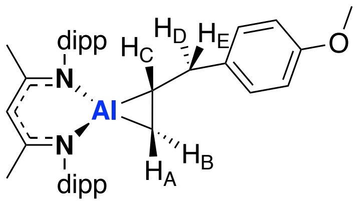

4-allylanisole: Reaction complete after 1 hour; 83 % yield versus ferrocene. The product was recrystallized from concentrated *n*-hexane at –35 °C. **2g:** ^{1}H NMR (400 MHz, benzene-d_6, 298 K): 0.31 (dd, 1H, C*H*^{B}, $^2J_{HH}$ = 12.1 Hz, $^3J_{HH}$ = 8.3 Hz), 0.93 (m, 1H, C*H*^{C}), 1.01 (d, 3H, CH(C*H*$_3$), $^3J_{HH}$ = 6.8 Hz), 1.02 (d, 3H, CH(C*H*$_3$), $^3J_{HH}$ = 6.8 Hz), 1.03 (1H, C*H*$_A$, under doublets), 1.14 (d, 3H, CH(C*H*$_3$), $^3J_{HH}$ = 6.8 Hz), 1.15 (d, 3H, CH(C*H*$_3$), $^3J_{HH}$ = 6.8 Hz), 1.28 (d, 3H, CH(C*H*$_3$), $^3J_{HH}$ = 6.8 Hz), 1.41 (d, 3H, CH(C*H*$_3$), $^3J_{HH}$ = 6.8 Hz), 1.45 (d, 3H, CH(C*H*$_3$), $^3J_{HH}$ = 6.8 Hz), 1.47 (d, 3H, CH(C*H*$_3$), $^3J_{HH}$ = 6.8 Hz), 1.51 (s, 6H, C*H*$_3$), 1.56 (s, 6H, C*H*$_3$), 1.82 (dd, 1H, C*H*$_D$ or C*H*$_E$, $^2J_{HH}$ = 14.5 Hz, $^3J_{HH}$ = 12.3 Hz), 2.92 (dd, 1H, C*H*$_D$ or C*H*$_E$, $^2J_{HH}$ = 14.5 Hz, $^3J_{HH}$ = 4.3 Hz), 3.30 (s, 3H, OC*H*$_3$), 3.37 (sept, 1H, C*H*(CH$_3$)$_2$, $^3J_{HH}$ = 6.8 Hz), 3.42 (sept, 1H, C*H*(CH$_3$)$_2$, $^3J_{HH}$ = 6.8 Hz), 3.48 (sept, 1H, C*H*(CH$_3$)$_2$, $^3J_{HH}$ = 6.8 Hz), 3.55 (sept, 1H, C*H*(CH$_3$)$_2$, $^3J_{HH}$ = 6.8 Hz), 4.83 (s, 1H, C(CH$_3$)C*H*C(CH$_3$)), 6.72 (m, 2H, Ar*H*), 7.00-7.20 (m, 8H, Ar*H*); ^{13}C{^{1}H} NMR (100 MHz, benzene-d_6, 298 K): 13.8 (Al*C*H$_A$H$_B$) 21.7 (Al*C*H$_C$), 22.8 (*C*H$_3$), 22.8 (*C*H$_3$), 24.0 (CH(*C*H$_3$)$_2$), 24.0 (2 x CH(*C*H$_3$)$_2$), 24.1 (CH(*C*H$_3$)$_2$), 24.3 (2 x CH(*C*H$_3$)$_2$), 24.4 (CH(*C*H$_3$)$_2$), 24.8 (CH(*C*H$_3$)$_2$), 28.2 (*C*H(CH$_3$)$_2$), 28.3 (*C*H(CH$_3$)$_2$), 28.8 (*C*H(CH$_3$)$_2$), 28.8 (*C*H(CH$_3$)$_2$), 40.0 (*C*H$_D$H$_E$), 54.3 (O*C*H$_3$), 95.9 (C(CH$_3$)C*H*C(CH$_3$)), 113.1 (*C*H), 124.1 (*C*H), 124.3 (*C*H), 124.5 (*C*H), 124.6 (*C*H), 127.6 (*C*H), 129.1 (*C*H), 138.1 (C^{IV}), 138.6 (C^{IV}), 138.7 (C^{IV}), 143.4 (C^{IV}), 143.6 (C^{IV}), 144.3 (C^{IV}), 144.5 (C^{IV}), 157.2 (C^{IV}), 172.3 (C(CH$_3$)CH*C*(CH$_3$)), 172.3 (*C*(CH$_3$)CHC(CH$_3$)).

2.2 Dimerization of 2b

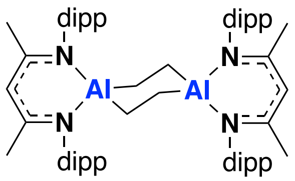

Synthesis of **3**: Complex **1** (15 mg, 0.03 mmol) was dissolved in benzene-d_6 (0.6 mL) and the solution transferred into a Young's tap NMR tube. The sample was then degassed (freeze/pump/thaw) three times, and the ethylene was added to the tube at approximately 1 bar pressure. The formation of compound **2b** was confirmed by ^{1}H NMR spectroscopy. The sample was kept at 298 K for 10 days, after which time quantitative conversion to product **3** was achieved. Compound **3** is insoluble in hydrocarbon solvents, thus preventing the recording of conversion versus an internal NMR standard. The dimerization reaction can be accelerated by heating the solution to 353 K for 30 minutes. X-ray quality crystals of **3** were grown from benzene-d_6 solution at room temperature. **3:** ^{1}H NMR (400 MHz, toluene-d_8, 298 K): -0.12 (s, 8H, AlCH_2), 1.02 (d, 24H, CH(CH_3)$_2$, $^3J_{HH}$ = 6.8 Hz), 1.16 (d, 24H, CH(CH_3)$_2$, $^3J_{HH}$ = 6.8 Hz), 1.44 (s, 12H, CH_3), 3.21 (sept, 8H, CH(CH$_3$)$_2$, $^3J_{HH}$ = 6.8 Hz), 4.69 (s, 2H, C(CH$_3$)CHC(CH$_3$)), 6.95–7.13 (m, 12H, ArH); ^{13}C{^{1}H} NMR (100 MHz, toluene-d_8, 298 K): 4.0 (AlCH$_2$), 23.9 (CH$_3$), 24.9 (CH(CH$_3$)$_2$), 25.5 (CH(CH$_3$)$_2$), 28.2 (CH(CH$_3$)$_2$), 95.4 (C(CH$_3$)CHC(CH$_3$)), 124.4 (CH), 127.0 (CH), 142.0 (C^{IV}), 144.3 (C^{IV}), 169.1 (C(CH$_3$)CHC(CH$_3$)). Anal. Calc. ($C_{62}H_{90}Al_2N_4$): C, 78.77; H, 9.60; N, 5.93. Found: C, 78.66; H, 9.49; N, 5.76.

2.3 Allylic sp^{3}C–H Activation

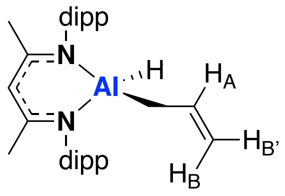

Complex **1** (15 mg, 0.03 mmol) was dissolved in benzene-d_6 (0.6 mL) and the solution transferred into a Young's tap NMR tube. The sample was then degassed (freeze/pump/thaw) three times, and the ethylene was added to the tube at approximately 1 bar pressure. The formation of compound **2c** was confirmed by ^{1}H NMR spectroscopy. The sample was then heated, in the presence of an excess of propylene, at 353 K overnight. The solvent was removed and X-ray quality crystals were isolated from concentrated *n*-hexane at –35 °C.

2c: ^{1}H NMR (400 MHz, benzene-d_6, 298 K): 1.09 (d, 6H, CH(CH_3)$_2$, $^3J_{HH}$ = 6.8 Hz), 1.17 (d, 6H, CH(CH_3)$_2$, $^3J_{HH}$ = 6.8 Hz), 1.29 (d, 2H, AlCH_2, $^3J_{HH}$ = 6.8 Hz), 1.31 (d, 6H, CH(CH_3)$_2$, $^3J_{HH}$ = 6.8 Hz), 1.40 (d, 6H, CH(CH_3)$_2$, $^3J_{HH}$ = 6.8 Hz), 1.55 (s, 6H, CH_3), 3.30 (sept, 8H, CH(CH$_3$)$_2$, $^3J_{HH}$ = 6.8 Hz), 3.45 (sept, 8H, CH(CH$_3$)$_2$, $^3J_{HH}$ = 6.8 Hz), 4.38 (dm, 1H, CH_B, $^3J_{HH}$ = 16.8 Hz), 4.49 (dm, 1H, C$H_{B'}$, $^3J_{HH}$ = 10.1 Hz), 4.50 (bs, 1H, AlH), 4.91 (s, 1H, C(CH$_3$)CHC(CH$_3$)), 5.55 (m, 1H, CH_A), 7.00-7.15 (m, 6H, ArH).

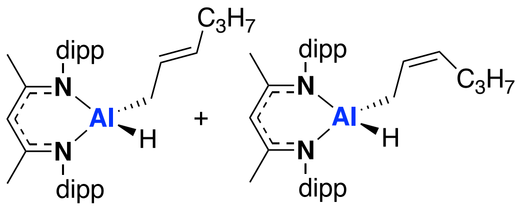

C–H Activation of Hex-1-ene: Complex **1** (5 mg, 0.01 mmol) and hex-1-ene (14 μL, 0.1 mmol) were dissolved in benzene-d_6 (0.6 mL) and the solution transferred into a Young's tap NMR tube. The formation of compound **cis/trans-2d** was confirmed by ^{1}H NMR spectroscopy. The sample was then heated, in the presence of an excess of the alkene, at 353 K overnight. Two new species were observed to form in a 1:0.6 ratio by ^{1}H NMR spectroscopy, however the diagnostic peaks of these products overlapped, preventing identification of the major isomer.

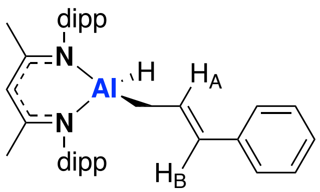

C–H Activation of Allylbenzene: Complex **1** (5 mg, 0.01 mmol) and allylbenzene (14.9 μL, 0.1 mmol) were dissolved in benzene-d_6 (0.6 mL) and the solution transferred into a Young's tap NMR tube. The formation of compound **2f** was confirmed by ^{1}H NMR spectroscopy. The sample was then heated, in the presence of an excess of the alkene, at 353 K overnight (92 % yield versus ferrocene). The solvent was removed and X-ray quality crystals were isolated from concentrated *n*-hexane at –35 °C. ***trans*-4f:** ^{1}H NMR (400 MHz, benzene-d_6, 298 K): 1.10 (d, 6H, CH(C*H*$_3$), $^3J_{HH}$ = 6.8 Hz), 1.11 (d, 6H, CH(C*H*$_3$), $^3J_{HH}$ = 6.8 Hz), 1.22 (d, 2H, AlC*H*$_2$, $^3J_{HH}$ = 8.8 Hz), 1.29 (d, 6H, CH(C*H*$_3$), $^3J_{HH}$ = 6.8 Hz), 1.36 (d, 6H, CH(C*H*$_3$), $^3J_{HH}$ = 6.8 Hz), 1.53 (s, 6H, C*H*$_3$), 3.33 (sept, 2H, C*H*(CH$_3$)$_2$, $^3J_{HH}$ = 6.8 Hz), 3.39 (sept, 2H, C*H*(CH$_3$)$_2$, $^3J_{HH}$ = 6.8 Hz), 4.50 (bs {FWHM ~ 180 Hz}, 1H, Al*H*), 4.89 (s, 1H, C(CH$_3$)C*H*C(CH$_3$)), 5.62 (d, 1H, C*H*^{B}, $^3J_{HH}$ = 15.6 Hz), 5.90 (dt, 1H, C*H*^{A}, $^3J_{HH}$ = 15.6 Hz, $^3J_{HH}$ = 8.8 Hz), 6.96-7.00 (m, 3H Ar*H*), 7.05-7.20 (m, 6H, Ar*H*); ^{13}C{^{1}H} NMR (100 MHz, benzene-d_6, 298 K): 17.3 (Al*C*H$_2$), 22.5 (*C*H$_3$), 23.8 (CH(*C*H$_3$)$_2$), 24.0 (CH(*C*H$_3$)$_2$), 24.6 (CH(*C*H$_3$)$_2$), 27.9 (*C*H(CH$_3$)$_2$), 28.6 (*C*H(CH$_3$)$_2$), 96.8 (C(CH$_3$)C*H*C(CH$_3$)), 123.1 (*C*H^B), 123.8 (*C*H), 124.5 (*C*H), 124.9 (*C*H), 125.1 (*C*H), 127.1 (*C*H), 127.8 (*C*H), 132.7 (*C*H^A), 139.8 (C^{IV}), 139.9 (C^{IV}), 142.8 (C^{IV}), 145.1 (C^{IV}), 170.0 (*C*(CH$_3$)CH*C*(CH$_3$)). Anal. Calc. ($C_{38}H_{51}AlN_2$): C, 81.09; H, 9.13; N, 4.98. Found: C, 80.98; H, 9.04; N, 4.95.

C–H Activation of 4-Allylanisole: **1** (5 mg, 0.01 mmol) and 4-allylanisole (17.2 μL, 0.1 mmol) were dissolved in benzene-d_6 (0.6 mL) and the solution transferred into a Young's tap NMR tube. The formation of compound **trans-4g** was confirmed by ^{1}H NMR spectroscopy. The sample was then heated, in the presence of an excess of the alkene, at 353 K overnight (95 % yield versus ferrocene). The solvent was removed and a colourless crystalline solid was isolated from *n*-hexane at –35 °C. ***trans*-4g:** ^{1}H NMR (400 MHz, benzene-d_6, 298 K): 1.10 (d, 6H, CH(C*H*$_3$), $^3J_{HH}$ = 6.8 Hz), 1.12 (d, 6H, CH(C*H*$_3$), $^3J_{HH}$ = 6.8 Hz), 1.21 (d, 2H, AlC*H*$_2$, $^3J_{HH}$ = 8.5 Hz), 1.31 (d, 6H, CH(C*H*$_3$), $^3J_{HH}$ = 6.8 Hz), 1.37 (d, 6H, CH(C*H*$_3$), $^3J_{HH}$ = 6.8 Hz), 1.53 (s, 6H, C*H*$_3$), 3.32 (s, 3H, OC*H*$_3$), 3.34 (sept, 1H, C*H*(CH$_3$)$_2$, $^3J_{HH}$ = 6.8 Hz), 3.41 (sept, 1H, C*H*(CH$_3$)$_2$, $^3J_{HH}$ = 6.8 Hz), 4.57 (bs {FWHM ~ 150 Hz), 4.89 (s, 1H, C(CH$_3$)C*H*C(CH$_3$)), 5.62 (d, 1H, C*H*^{B}, $^3J_{HH}$ = 15.6 Hz), 5.75 (dt, 1H, C*H*^{A}, $^3J_{HH}$ = 15.6 Hz, $^3J_{HH}$ = 8.5 Hz), 6.75 (dm, 2H, Ar*H*, $^3J_{HH}$ = 8.8 Hz), 6.91 (dm, 2H, Ar*H*, $^3J_{HH}$ = 8.8 Hz), 7.06-7.20 (m, 6H, Ar*H*); ^{13}C{^{1}H} NMR (100 MHz, benzene-d_6, 298 K): 16.8 (Al*C*H$_2$), 22.5 (*C*H$_3$), 23.8 (CH(*C*H$_3$)$_2$), 24.0 (CH(*C*H$_3$)$_2$), 24.5 (CH(*C*H$_3$)$_2$), 25.7 (CH(*C*H$_3$)$_2$), 27.9 (*C*H(CH$_3$)$_2$), 28.6 (*C*H(CH$_3$)$_2$), 54.3 (O*C*H$_3$), 96.8 (C(CH$_3$)C*H*C(CH$_3$)), 113.4 (*C*H), 122.7 (*C*H^B), 123.8 (*C*H), 124.8 (*C*H), 126.1 (*C*H), 127.1 (*C*H), 130.2 (*C*H^A), 132.9 (C^{IV}), 139.9 (C^{IV}), 142.9 (C^{IV}), 145.1 (C^{IV}), 157.4 (C^{IV}), 170.0 (*C*(CH$_3$)CH*C*(CH$_3$)). Anal. Calc. ($C_{39}H_{53}AlN_2$): C, 79.01; H, 9.01; N, 4.73. Found: C, 78.92; H, 9.18; N, 4.58.

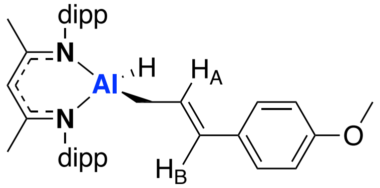

3. Reversible Alkene Binding to 1

3.1 Variable Temperature NMR of metallocyclopropanes

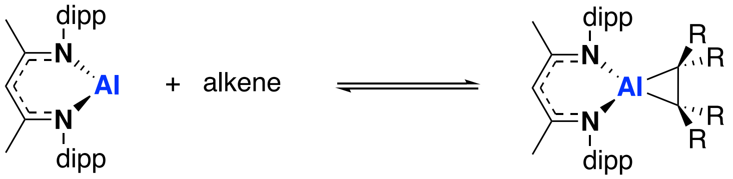

1

Complex **1** (15 mg, 0.3 mmol) and an excess of the alkene (0.1 mmol) were dissolved in benzene-d_6 (0.6 mL) and the solution transferred into a Young's tap NMR tube. The formation of the previously characterized (vide supra) metallocyclopropane was confirmed by ^{1}H NMR spectroscopy. The excess alkene was removed *in vacuo* after which time the sample was dissolved in toluene-d_8 (0.6 mL) and left to equilibrate at 298 K for 24 hours. Variable temperature ^{1}H NMR analysis was performed on the sample over the temperature range 343–373 K. At temperatures lower than 353 K it was found the be necessary to hold the sample at temperature for a prolonged period to allow equilibrium to be reached (e.g. for a sample of compound **2a**, this was found to take 15 minutes at 343 K).

3.2 Van't Hoff Analysis

$$K_{eq} = \frac{[\text{aluminiumcyclopropane}]}{[\mathbf{1}][\text{alkene}]}$$

The Van't Hoff equation was used in order to determine $\boldsymbol{\Delta H}$ and $\boldsymbol{\Delta S}$ from the slope and the intercept of the plot of $\ln(K_{eq})$ versus 1/T. $\boldsymbol{\Delta G}$ was calculated according to Gibb's Free Energy equation.

Figure S1: Variable temperature ^{1}H NMR spectra of **2a** (343–373 K) in toluene-d_8.

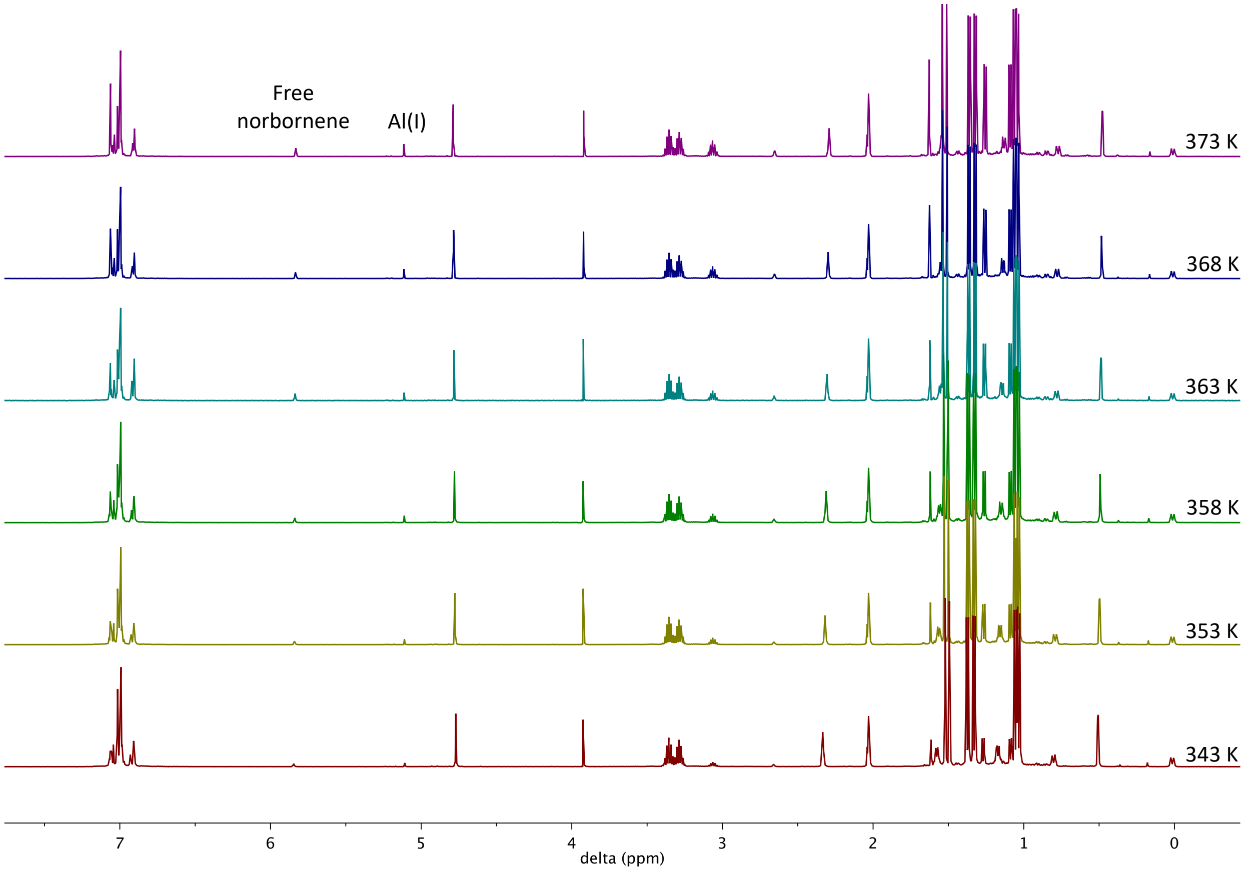

Figure S2: Van't Hoff analysis for the equilibrium between **1, 2a** and norbornene.

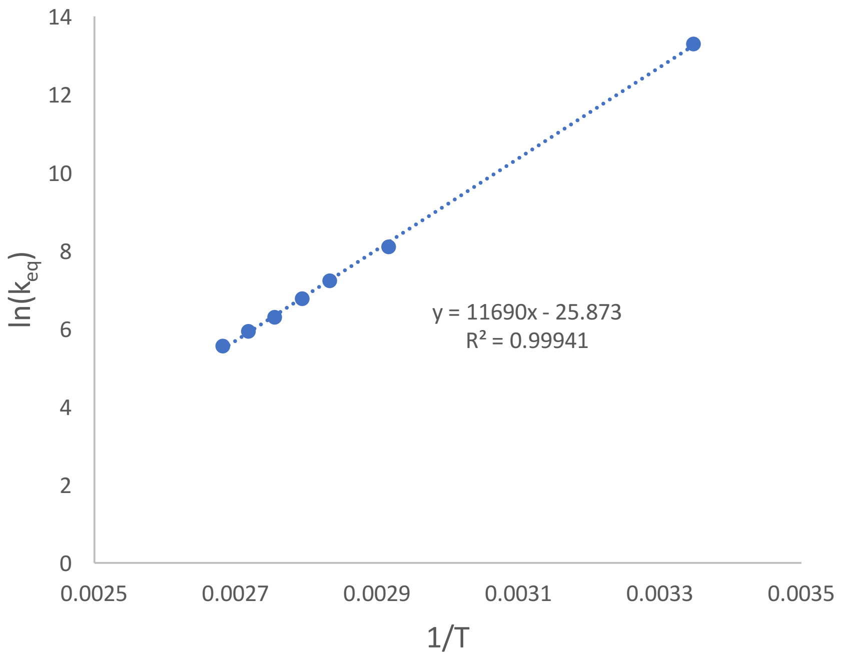

**ΔS**	–51 cal K^{-1} mol^{-1}
**ΔH**	–23.2 kcal mol^{-1}
**ΔG**	–7.9 kcal mol^{-1}

Figure S3: Variable temperature ^{1}H NMR spectra of **2d** (343–373 K) in toluene-d_8.

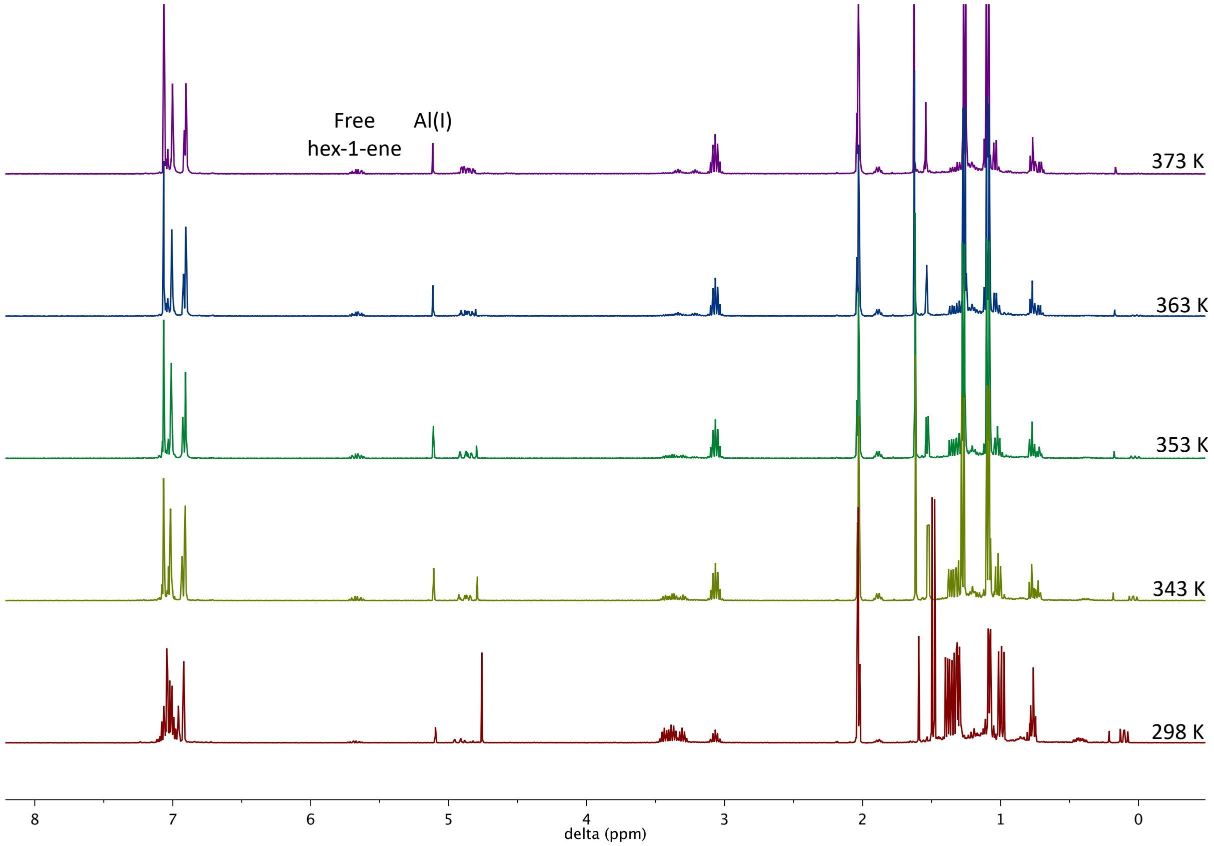

Figure S4: Van't Hoff analysis for the equilibrium between **1**, **2d** and hex-1-ene.

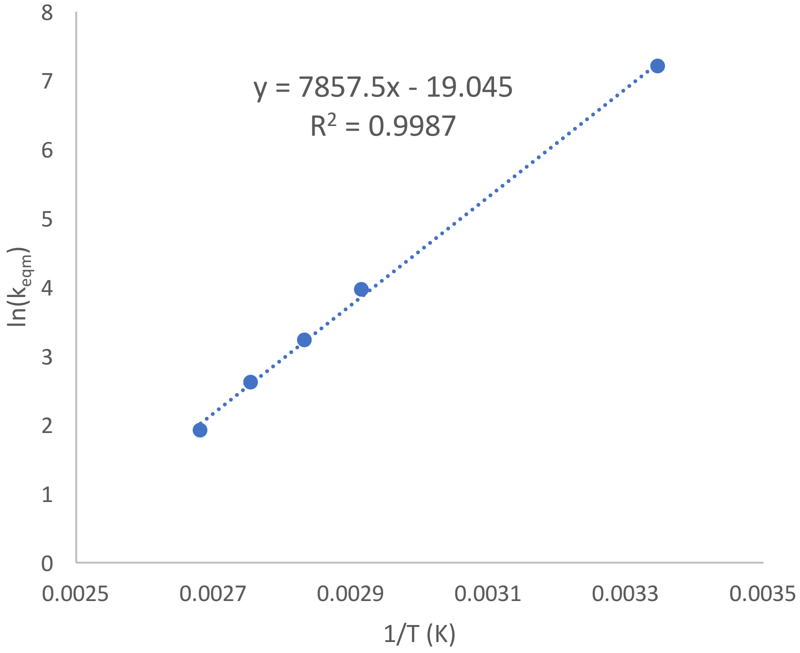

**ΔS**	–38 cal K^{-1} mol^{-1}
**ΔH**	–15.6 kcal mol^{-1}
**ΔG**	–4.3 kcal mol^{-1}

The variable temperature ^{1}H NMR spectrum of compound **2b** showed the formation of **1** and free ethylene. However, a significant amount of degradation or further reaction also occurs (*note:* Compound **2b** further reacts to form **3**), therefore it was not possible to perform van't Hoff analysis on the sample.

Figure S5: Variable temperature ^{1}H NMR spectra of **2b** (343–373 K) in toluene-d_8.

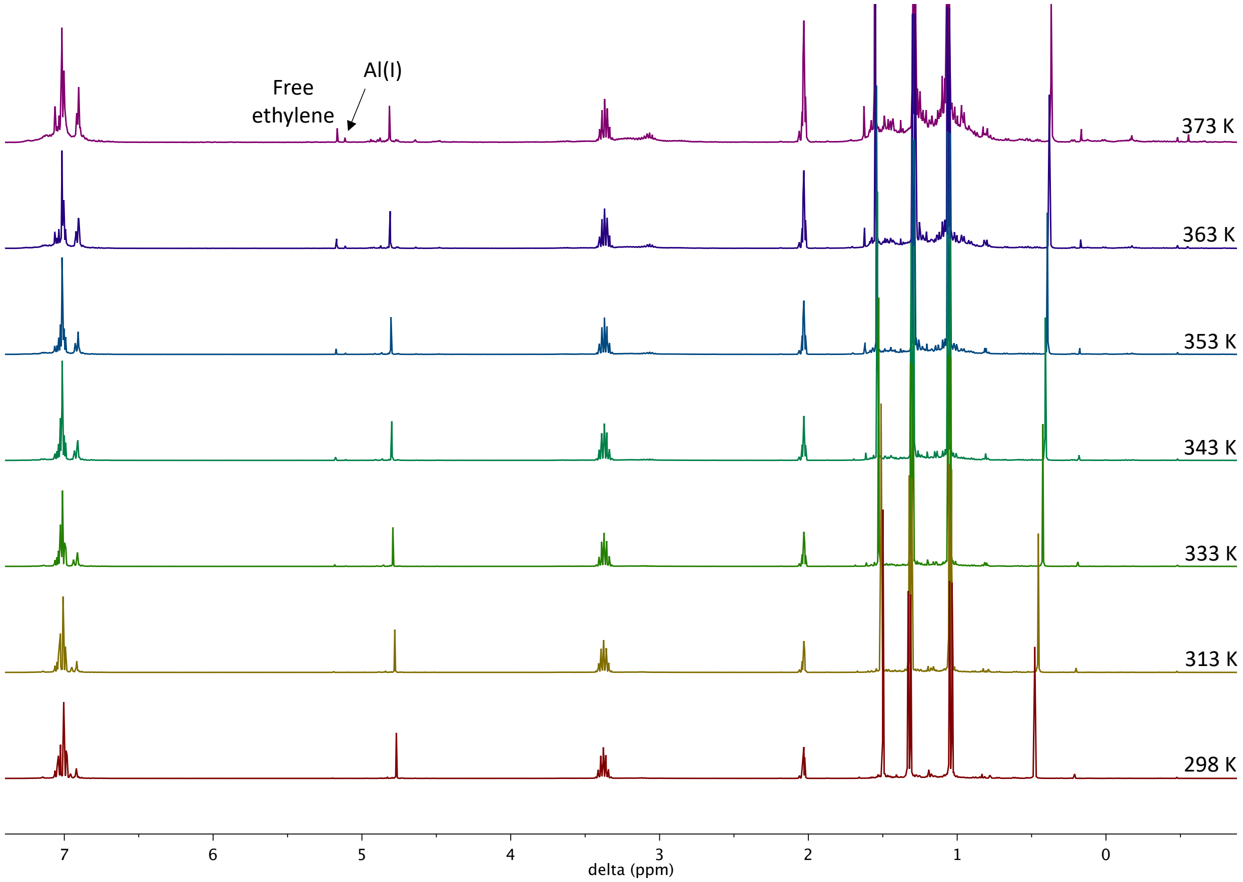

4. Kinetic Analysis

Complex **1** (5 mg, 0.01 mmol) was dissolved in toluene-d_8 (0.6 mL) and five equivalents of allylbenzene (7.5 μL, 0.05 mmol) were added along with a small amount of ferrocene (2 mg), to be used as an internal standard. The solution transferred into a Young's tap NMR tube and the formation of compound **2f** was confirmed by ^{1}H NMR spectroscopy. Kinetic analysis was conducted using compound **1** at 18.7 mM concentration in toluene-d_8 over a range of temperatures (343 - 363 K). ^{1}H NMR spectra were recorded at regular intervals throughout the reaction, and the data was processed using Topspin and MNova software. The ferrocene was used as an internal standard to ensure that the overall concentration of substrates remained consistent throughout the reaction. The data was modelled using Copasi software consider two pre-equilibria scenarios: model A **2f** = **1** + alkene -> **4f** and model B **1** + alkene = **2f** -> **4f**. While these models can not be distinguished experimentally, model A is unambiguously favoured by the DFT calculations (*vide infra).*

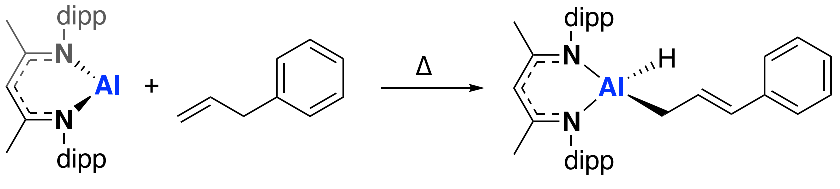

Figure S6: Plot of [Species] vs time of experimental (dots) and Copasi fitted (smooth lines) data for the reaction of **2f** (yellow) to **4f** (blue), via **1** (green) at 343 K.

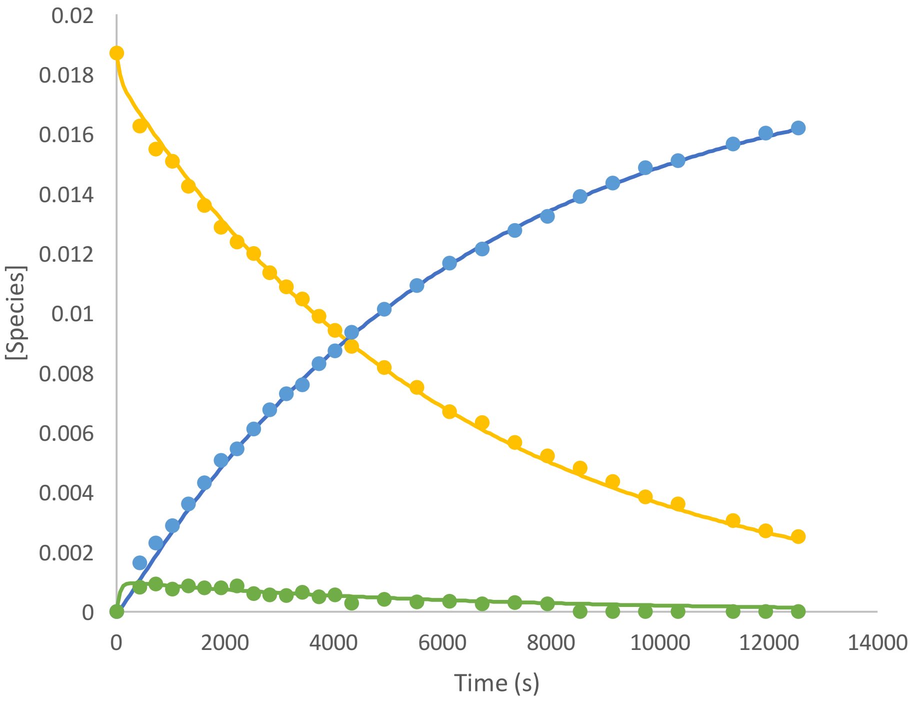

Figure S7: Plot of [Species] vs time of experimental (dots) and Copasi fitted (smooth lines) data for the reaction of **2f** (yellow) to **4f** (blue), via **1** (green) at 353 K.

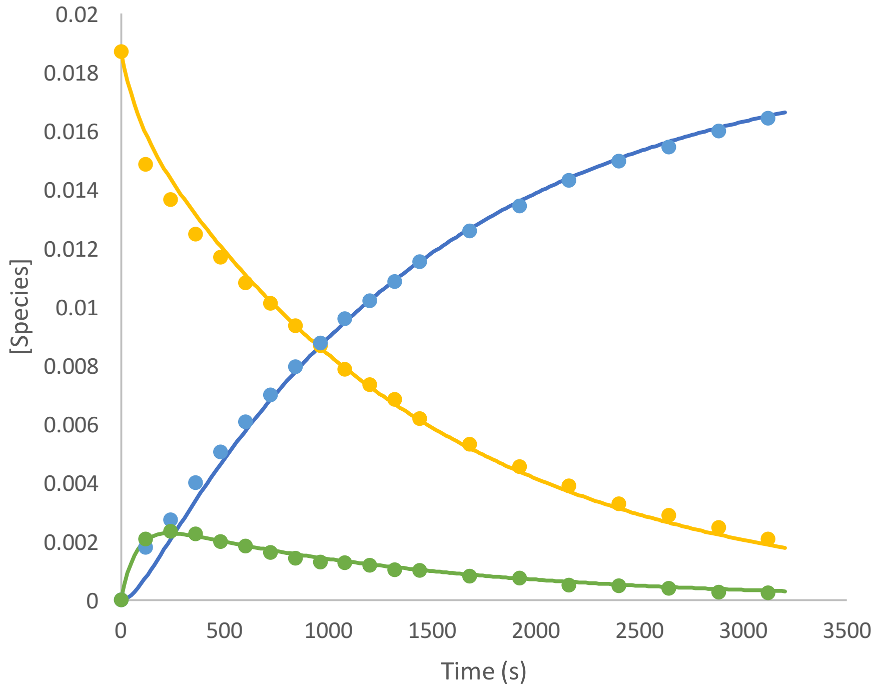

Figure S8: Plot of [Species] vs time of experimental (dots) and Copasi fitted (smooth lines) data for the reaction of **2f** (yellow) to **4f** (blue), via **1** (green) at 358 K.

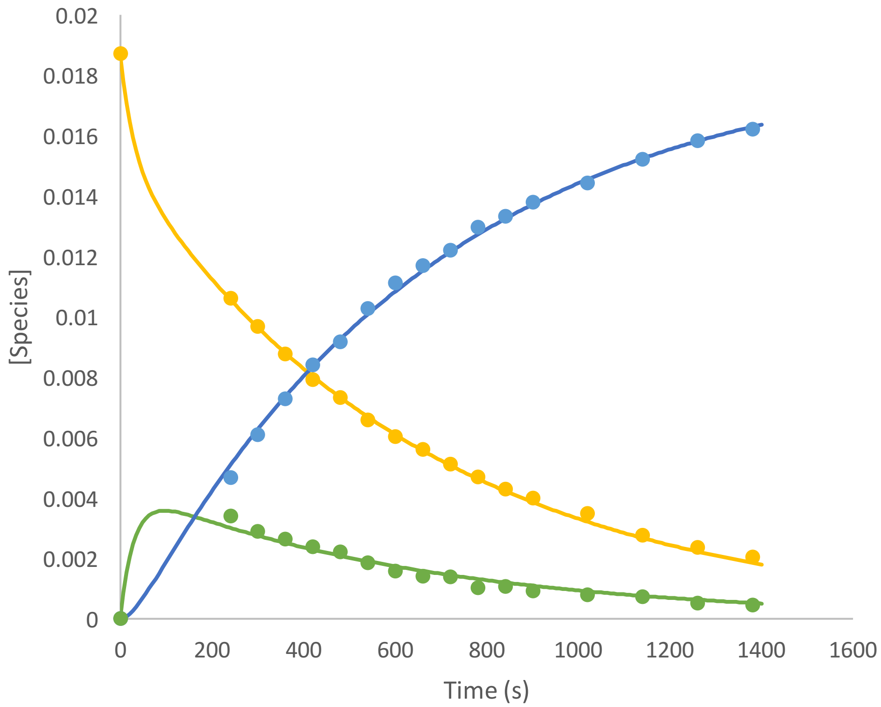

Figure S9: Plot of [Species] vs time of experimental (dots) and Copasi fitted (smooth lines) data for the reaction of **2f** (yellow) to **4f** (blue), via **1** (green) at 363 K.

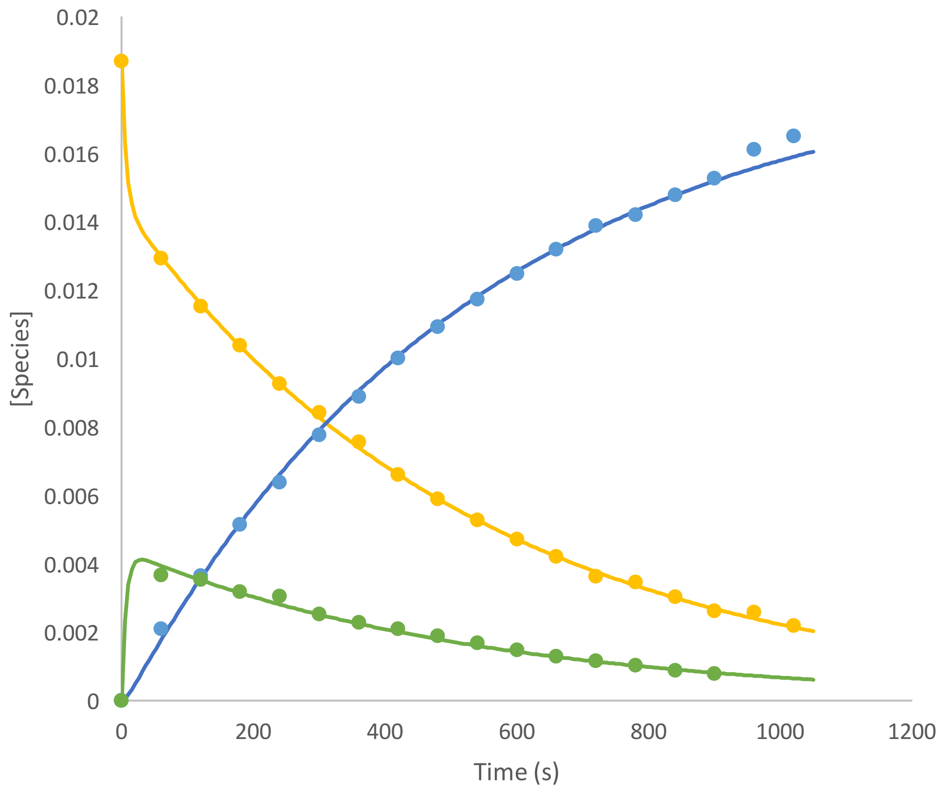

Model A:

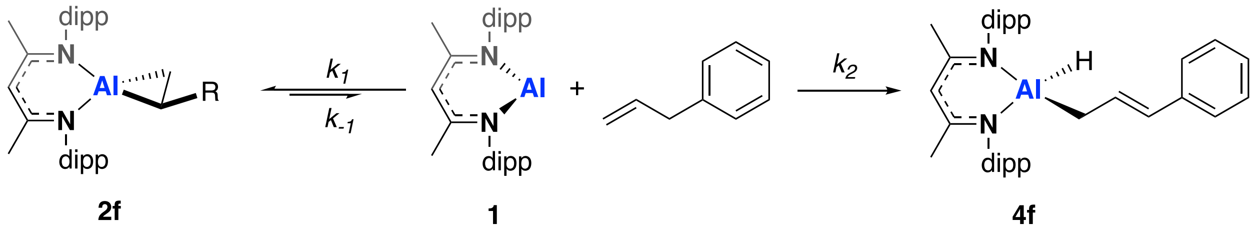

Table S1: Table of rate constants obtained from the Copasi generated models over the temperature range 343–363 K.

	**k_{-1}**	**k_1**	**k_2**	**k_{-1}/k_1**
**343 K**	0.0146538	0.25983	0.00299006	0.05639764
**353 K**	0.0099177	0.0568375	0.00478135	0.17449219
**358 K**	0.00586177	0.0150144	0.00692216	0.39040987
**363 K**	0.0343894	0.106978	0.0080431	0.32146236

Figure S10: Eyring analysis: ln{k_2/T} versus 1/T for the reaction of **1** to **4f** (where k_2 is a modelled rate constant).

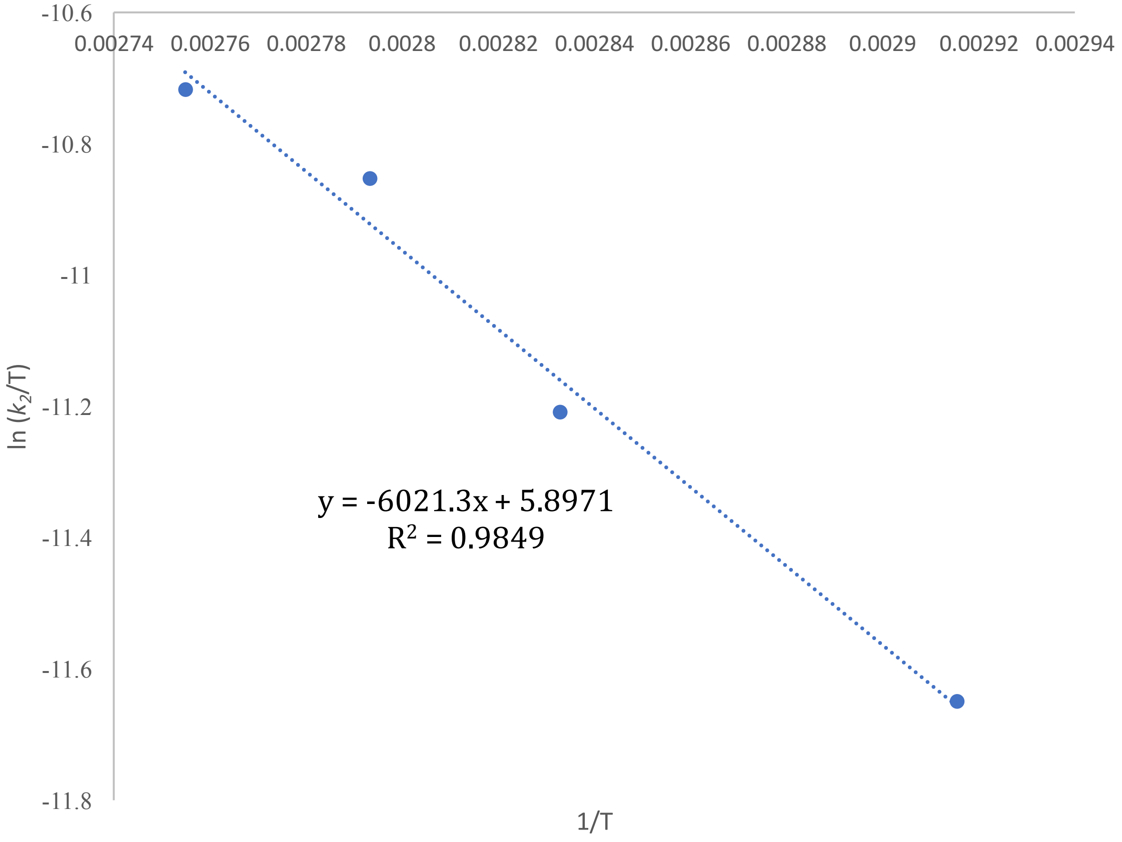

$\Delta S^{\ddagger}$	–35.5 cal K^{-1} mol^{-1}
$\Delta H^{\ddagger}$	+10.0 kcal mol^{-1}
$\Delta G^{\ddagger}$	+20.5 kcal mol^{-1}

Model B

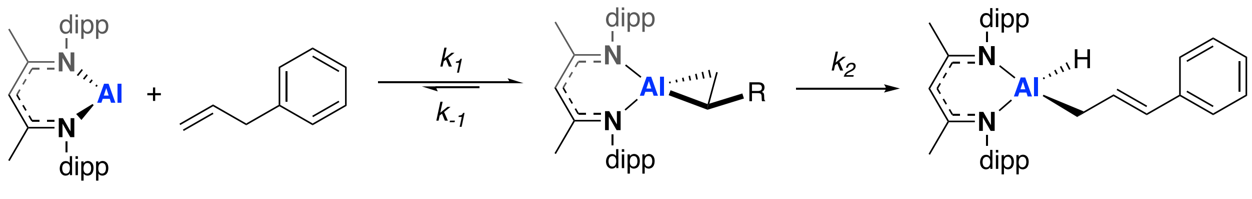

1 2f 4f

Table S2: Table of rate constants obtained from the Copasi generated models over the temperature range 343–363 K.

	k_{-1}	k_1	k_2	k_{-1}/k_1
**343 K**	0.00865515	0.00047488	0.00016647	18.2259777
**353 K**	0.2177802	0.03481205	0.00076695	6.25588564
**358 K**	0.15895	0.0467458	0.00185207	3.40035048
**363 K**	0.21026567	0.06273438	0.00239816	3.35168175

Figure S11: Eyring analysis: ln{k_2/T} versus 1/T for the reaction of **2f** to **4f** (where k_2 is a modelled rate constant).

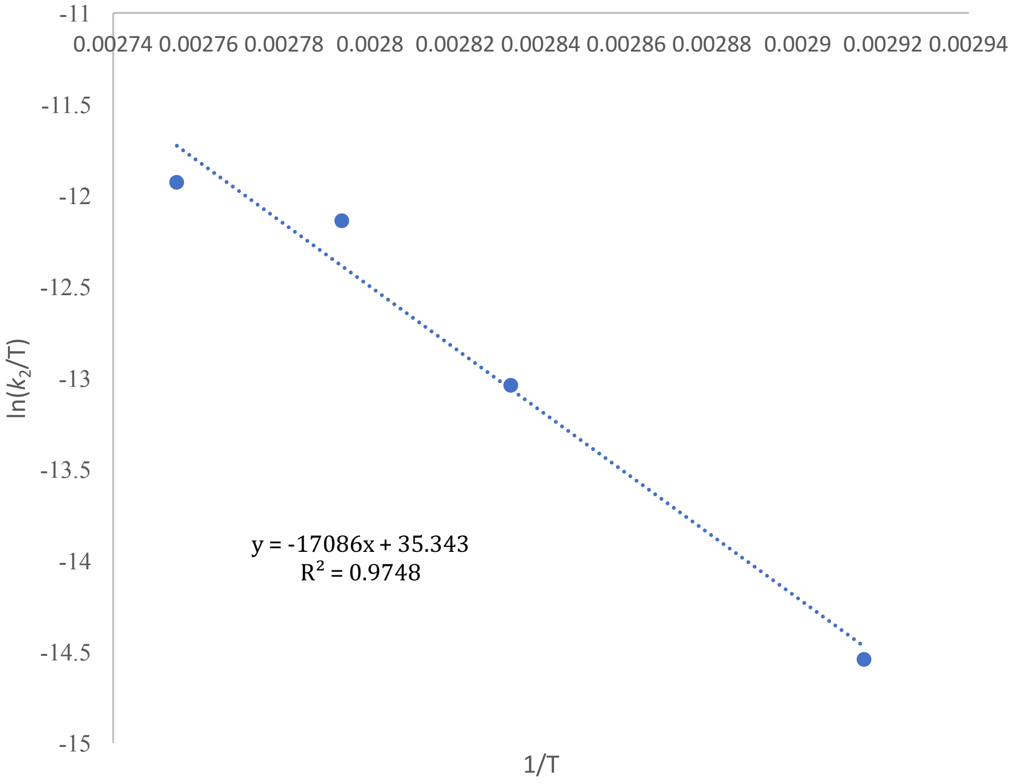

**Δ$S^{‡}$**	+23.0 cal K^{-1} mol^{-1}
**Δ$H^{‡}$**	+33.9 kcal mol^{-1}
**Δ$G^{‡}$**	+27.1 kcal mol^{-1}

5. X-ray Crystallographic Data

The X-ray crystal structure of 2b

Crystal Data for **2b:** $C_{31}H_{45}AlN_2$, M =472.67, monoclinic, P2_1/n (no. 14), a = 12.3658(5) Å, b = 17.1209(6) Å, c = 14.0230(5) Å, β = 104.507(4)°, V = 2874.20(18) Å^3, Z = 4, ρ_{calc}g/cm^3 = 1.092, μ(MoKα) = 0.091 mm^{-1}, T = 172.95(10), orange blocks, F^2 refinement, R_1(obs) = 0.0465, wR_2(all) = 0.1255, 5791 independent observed reflections (R_{int} = 0.0242), 4461 independent measured reflections [|F_o| > 4σ(|F_o|), 2θ_{full} = 56.326], 317 parameters. CCDC 1870241.

The X-ray crystal structure of 2d

Crystal data for **2d**: $C_{35}H_{53}AlN_2$·0.5(C_7H_8), M = 574.84, monoclinic, $C2/c$ (no. 15), a = 19.4468(12), b = 18.4781(7), c = 20.5105(13) Å, β = 102.346(7)°, V = 7199.8(7) Å^3, Z = 8, D_c = 1.061 g cm^{-3}, μ(Mo-Kα) = 0.083 mm^{-1}, T = 173 K, dark orange blocks, Agilent Xcalibur 3 E diffractometer; 7244 independent measured reflections (R_{int} = 0.0203), F^2 refinement,[2,3] R_1(obs) = 0.0752, wR_2(all) = 0.2093, 4860 independent observed absorption-corrected reflections [|F_o| > 4σ(|F_o|), completeness to θ_{full}(25.2°) = 98.8%], 428 parameters. CCDC 1870239.

The C18-based 2,6-diisopropylphenyl group in the structure of **2d** was found to be disordered. Two orientations were identified of *ca*. 80 and 20% occupancy, their geometries were optimised, the thermal parameters of adjacent atoms were restrained to be similar, and only the non-hydrogen atoms of the major occupancy orientation were refined anisotropically (those of the minor occupancy orientation were refined isotropically). The C31-based hexyl group was found to be disordered, and three orientations were identified of *ca*. 43, 34 and 23% occupancy. The geometries of the three orientations were optimised, the thermal parameters of adjacent atoms were restrained to be similar, and all of the non-hydrogen atoms were refined isotropically. The C40-based included toluene solvent molecule was found to be disordered across a centre of symmetry, and two unique orientations were identified of *ca*. 33 and 17% occupancy (with two further orientations of the same occupancies being generated by operation of the inversion centre). The geometries of both unique orientations were optimised, the thermal parameters of adjacent atoms were restrained to be similar, and all of the atoms of both orientations were refined isotropically.

The X-ray crystal structure of 2g

Crystal Data for **2g***:* $C_{39}H_{53}AlN_2O$, M =592.81, monoclinic, P2_1/n (no. 14), a = 12.3296(4) Å, b = 16.8397(5) Å, c = 17.7035(6) Å, β = 106.384(4)°, V = 3526.5(2) Å^3, Z = 4, ρ_{calc}g/cm^3 = 1.117, μ(MoKα) = 0.089 mm^{-1}, T = 173.00(14), red blocks, F^2 refinement, R_1(obs) = 0.0650, wR_2(all) = 0.1647, 7084 independent observed reflections (R_{int} = 0.0219), 5230 independent measured reflections [|F_o| > 4σ(|F_o|), 2θ_{full} = 56.364], 399 parameters. CCDC 1870242.

The X-ray crystal structure of 3

Crystal data for **3**: $C_{62}H_{90}Al_2N_4$, *M* = 945.33, monoclinic, *C*2/*c* (no. 15), *a* = 24.6053(3), *b* = 15.38681(19), *c* = 14.82658(16) Å, β = 96.6729(11)°, *V* = 5575.27(11) Å^3, *Z* = 4 [C_i symmetry], D_c = 1.126 g cm^{-3}, μ(Cu-Kα) = 0.772 mm^{-1}, *T* = 173 K, yellows blocks, Agilent Xcalibur PX Ultra A diffractometer; 5366 independent measured reflections (R_{int} = 0.0208), F^2 refinement,[2,3] R_1(obs) = 0.0401, wR_2(all) = 0.1122, 4618 independent observed absorption-corrected reflections [$|F_o| > 4\sigma(|F_o|)$, completeness to θ_{full}(67.7°) = 98.5%], 317 parameters. CCDC 1870240.

The structure of **3** was found to sit across a centre of symmetry at the middle of the C_4Al_2 ring.

The X-ray crystal structure of 4c

Crystal data for **4c**: $C_{32}H_{47}AlN_2$, *M* = 486.69, monoclinic, *P*2$_1$/*c* (no. 14), *a* = 10.5476(5), *b* = 13.0639(6), *c* = 22.5422(13) Å, β = 99.217(5)°, *V* = 3066.0(3) Å^3, *Z* = 4, D_c = 1.054 g cm^{-3}, μ(Mo-Kα) = 0.087 mm^{-1}, *T* = 173 K, colourless blocks, Agilent Xcalibur 3 E diffractometer; 6182 independent measured reflections (R_{int} = 0.0241), F^2 refinement,[2,3] R_1(obs) = 0.0586, wR_2(all) = 0.1591, 4459 independent observed absorption-corrected reflections [$|F_o| > 4\sigma(|F_o|)$, completeness to θ_{full}(25.2°) = 99.2%], 342 parameters. CCDC 1870238.

The Al–H hydrogen atom in the structure of **4c** was located from a Δ*F* map and refined freely. The C31-based allyl group was found to be disordered, and three orientations were identified of *ca*. 42, 39 and 19% occupancy. The geometries of the three orientations were optimised, the thermal parameters of adjacent atoms were restrained to be similar, and all of the non-hydrogen atoms were refined isotropically.

The X-ray crystal structure of 4f

Crystal Data for **4f***:* $C_{38}H_{50}AlN_2$, *M* =561.78, orthorhombic, P2$_1$2$_1$2$_1$ (no. 19), *a* = 11.2270(4) Å, *b* = 14.6841(6) Å, *c* = 21.0838(9) Å, *V* = 3475.8(2) Å^3, *Z* = 4, ρ_{calc}g/cm^3 = 1.074, μ(MoKα) = 0.085 mm^{-1}, *T* = 172.95(10), colourless plates, F^2 refinement, R_1(obs) = 0.0502, wR_2(all) = 0.1458, 6159 independent observed reflections (R_{int} = 0.0200), 4682 independent measured reflections [$|F_o| > 4\sigma(|F_o|)$, $2\theta_{full}$ = 56.45], 405 parameters. CCDC 1870243.

The allylbenzene group was found to be disordered with major and minor occupancies ca. 66% and 34%. Non-hydrogen atoms of the major occupancy orientation were refined anisotropically (those of the minor occupancy orientation were refined isotropically).

Figure S12: The crystal structure of **2d** (50% probability ellipsoids).

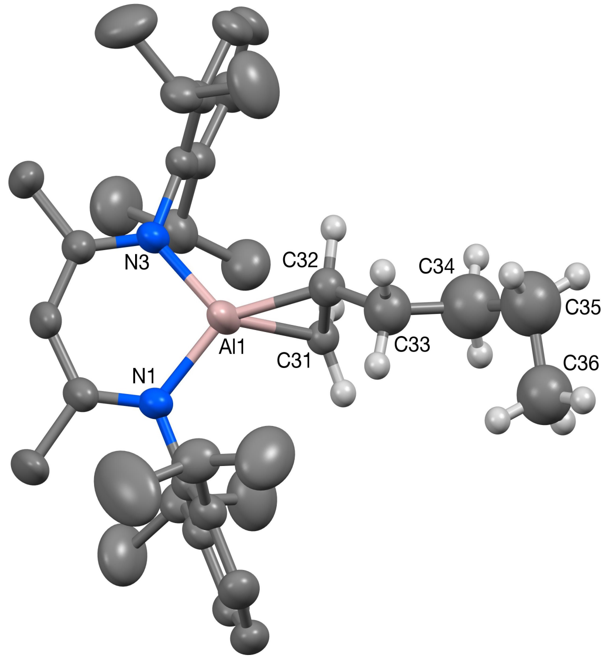

Figure S13: The crystal structure of **4c** (50% probability ellipsoids).

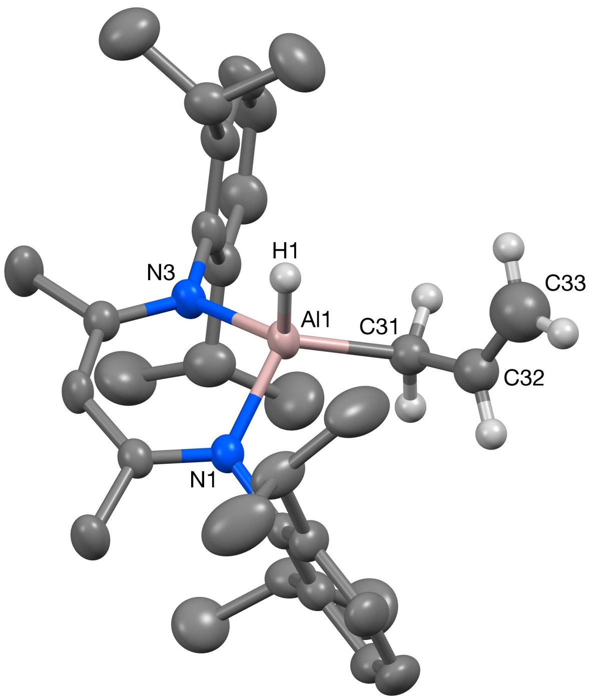

6. Computational Details

DFT calculations were run using Gaussian 09 (Revision D.01)[4] using the M06L Minnesota density functional. Al centers were described with Stuttgart SDDAll RECPs and associated basis sets and the 6-31G** basis sets were used for all other atoms.[5,6,7] Alternate functions were also investigated: ωB97X, M062X, B3PW91.

Geometry optimisation calculations were performed without symmetry constraints. Frequency analyses for all stationary points were performed using the enhanced criteria to confirm the nature of the structures as either minima (no imaginary frequency) or transition states (only one imaginary frequency).

Intrinsic reaction coordinate (IRC) calculations were used to connect transition states and minima located on the potential energy surface allowing a full energy profile (calculated at 298.15 K, 1 atm) of the reaction to be constructed.[8,9] Free energies reported within the main text are corrected for the effects of benzene solvent (ε=2.2706) using the using the polarizable continuum model (PCM).[10] In addition, single point dispersion corrections were applied to the ωB97X optimised geometries (dispersion corrected ωB97X-D functional) and to the B3PW91 functional (dispersion corrected GD3BJ functional).[11] QTAIM calculations were run with the AIMAll package.

The graphical user interface used to visualise the various properties of the intermediates and transition states was GaussView 5.0.9.[12] Natural Bond Orbital analysis was carried out using NBO 6.0.[13]

6.1 Calculated Reaction Pathways

Figure S14a: Selected bond lengths (Å) for **TS-1** (**ethylene**); M06L, Al (SDDAll), C H N (6-31G**). Some Hydrogen atoms have been omitted for clarity.

.

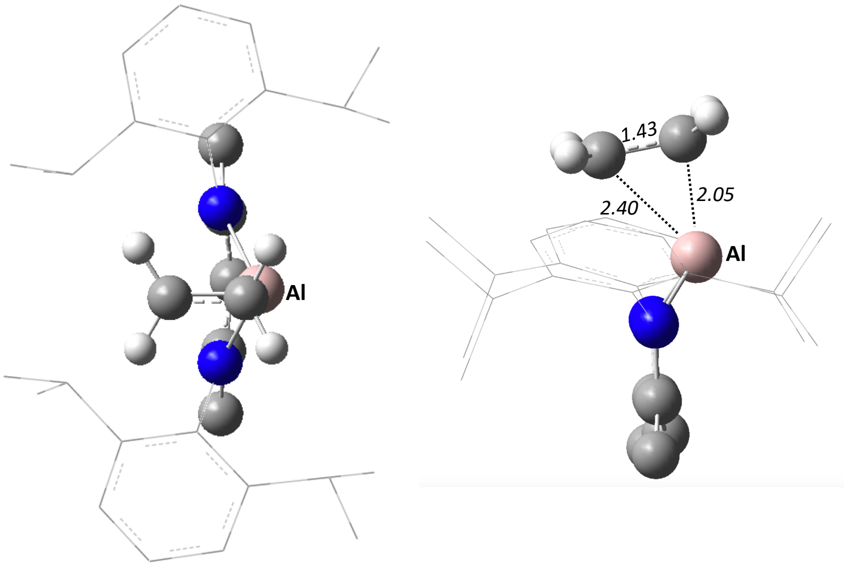

Figure S14b. QTAIM molecular graphs of (a) **TS-1 (ethylene)** and (b) **2b**

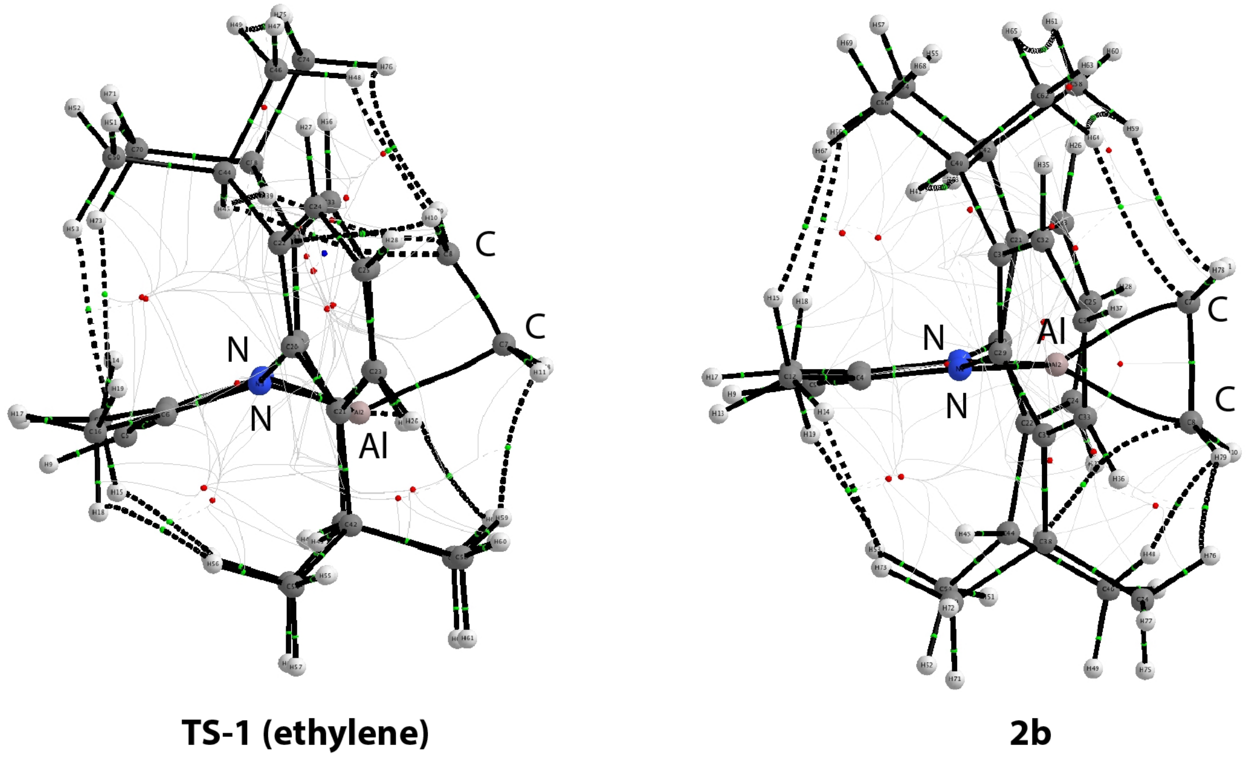

Figure S15: Front and side view of **endo-TS-1** with selected bond lengths (Å) (**propylene: endo approach**); M06L, Al (SDDAll), C H N (6-31G**). Some Hydrogen atoms have been omitted for clarity.

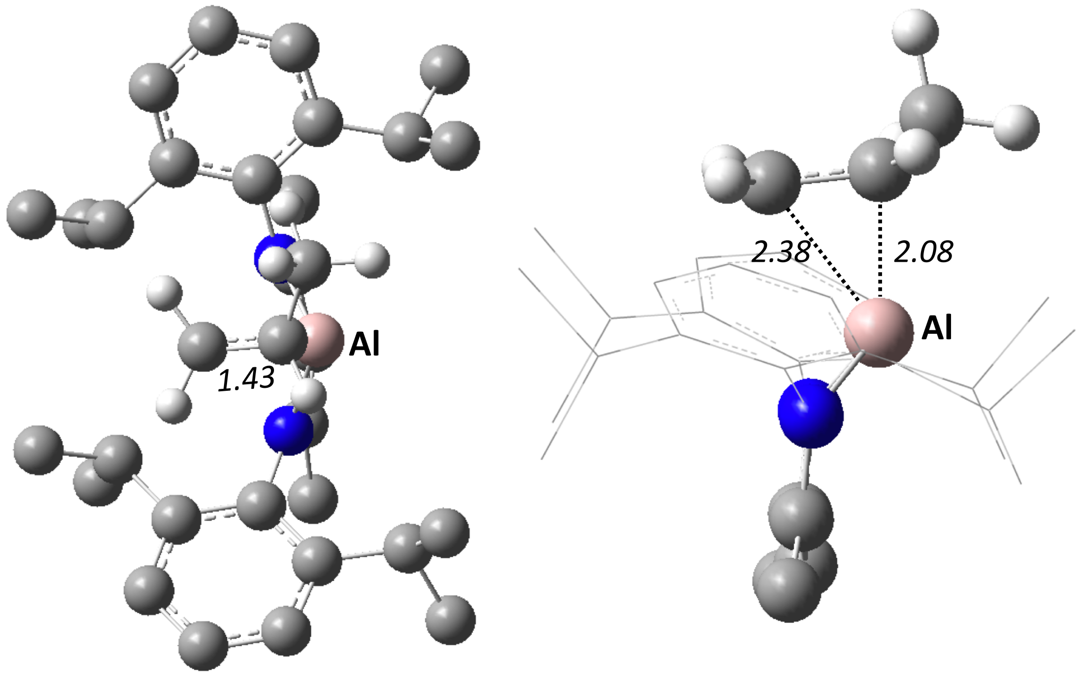

Figure S16: Front and side view of **exo-TS-1** with selected bond lengths (Å) (**propylene: exo approach**); M06L, Al (SDDAll), C H N (6-31G**). Some Hydrogen atoms have been omitted for clarity.

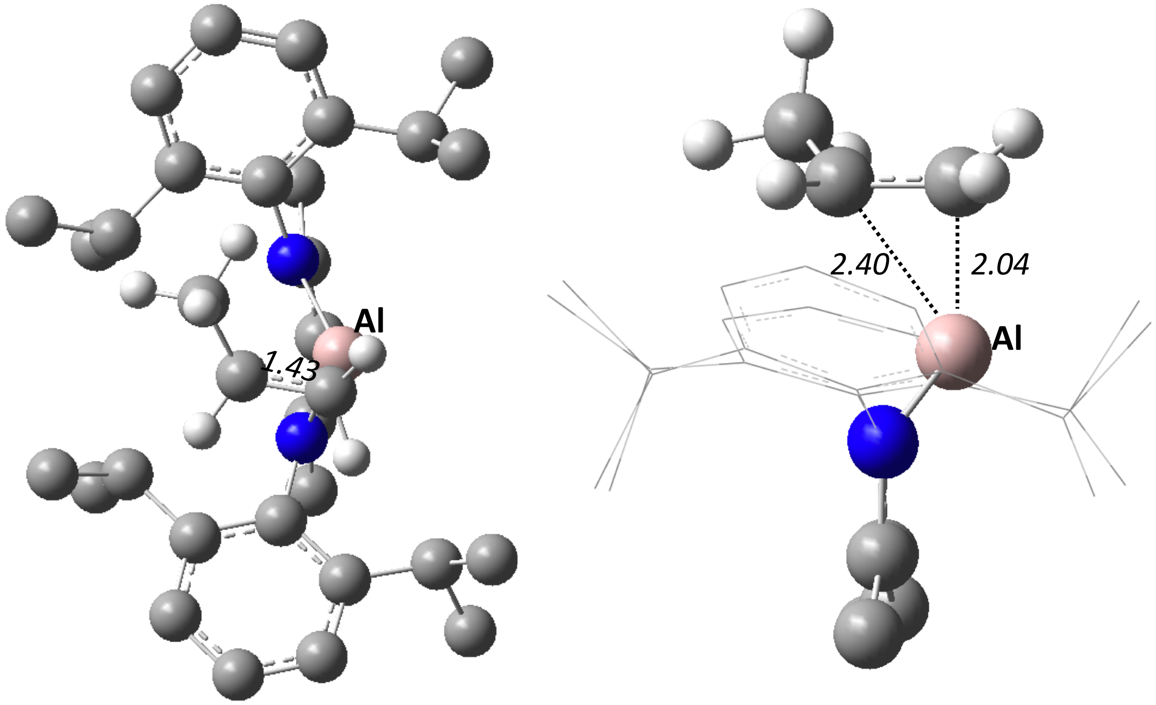

Figure S17: Two views of **TS-2** with selected bond lengths (Å) (**propylene C–H activation**); M06L, Al (SDDAll), C H N (6-31G**). Some Hydrogen atoms have been omitted for clarity.

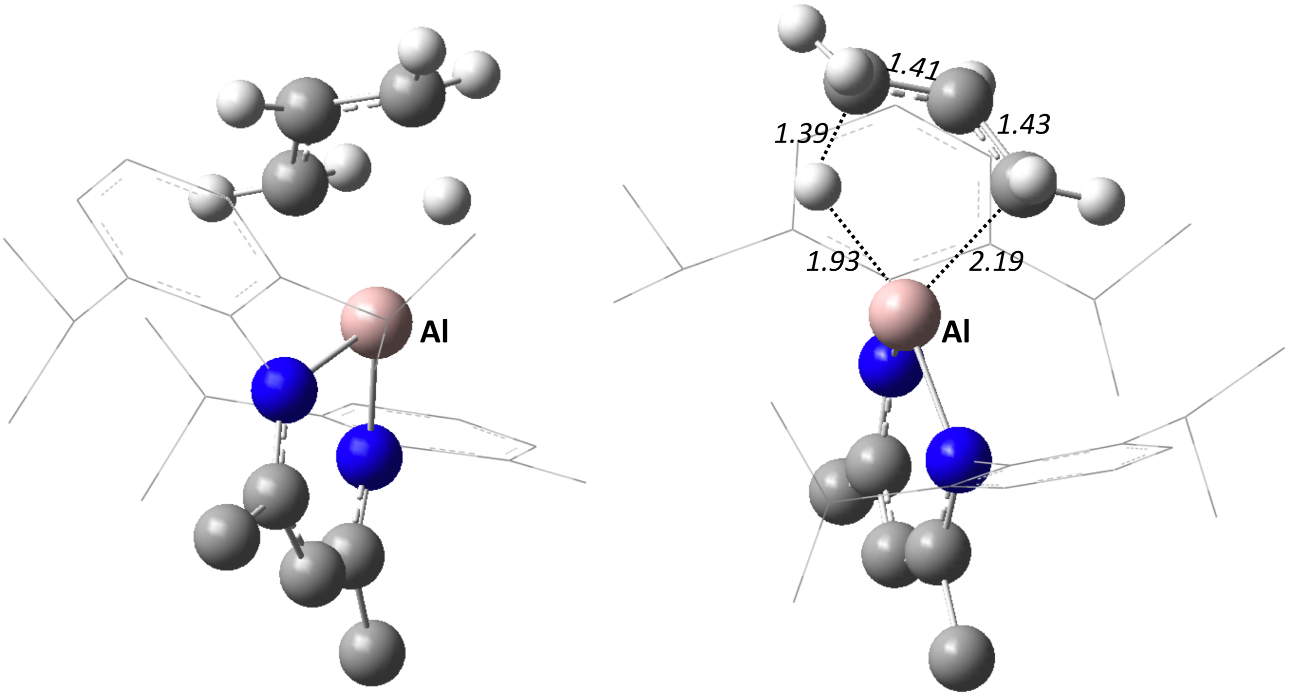

Figure S18: Selected bond lengths (Å) for **TS-1** (**allylbenzene: exo (LHS) and endo (RHS) approach**); M06L, Al (SDDAll), C H N (6-31G**). Some Hydrogen atoms have been omitted for clarity.

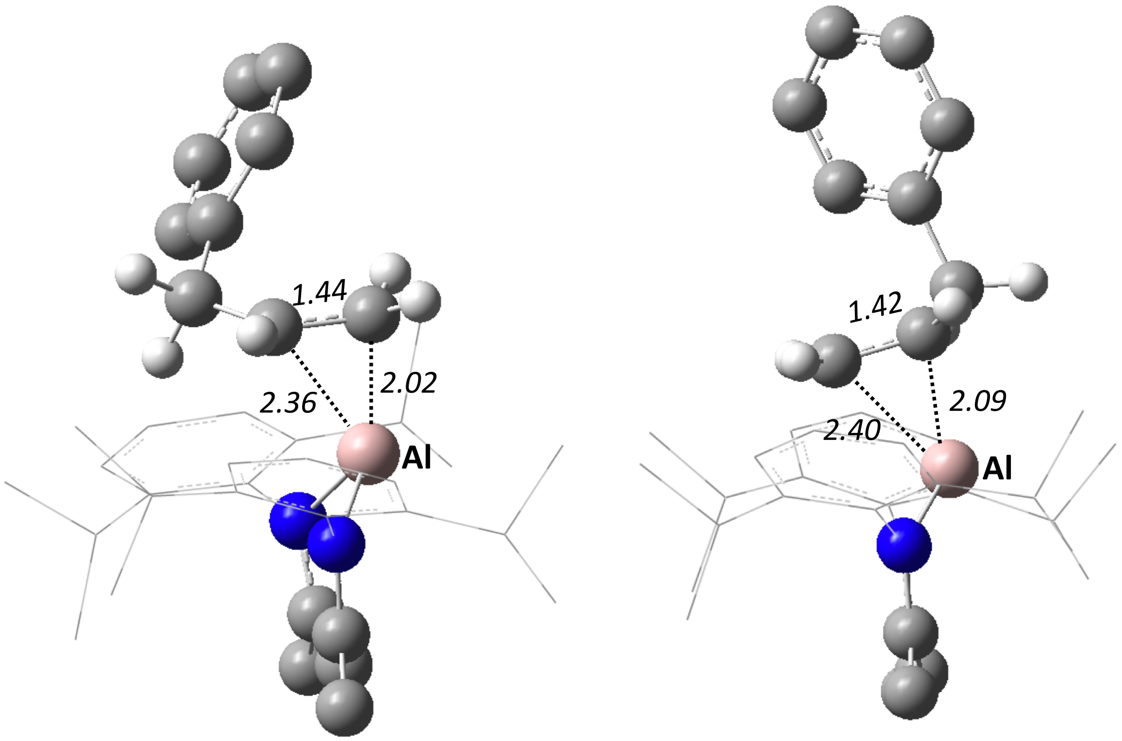

Figure S19: Selected bond lengths (Å) for **TS-3** (**allylbenzene: trans (LHS) and cis (RHS)**);; M06L, Al (SDDAll), C H N (6-31G**). Some Hydrogen atoms have been omitted for clarity.

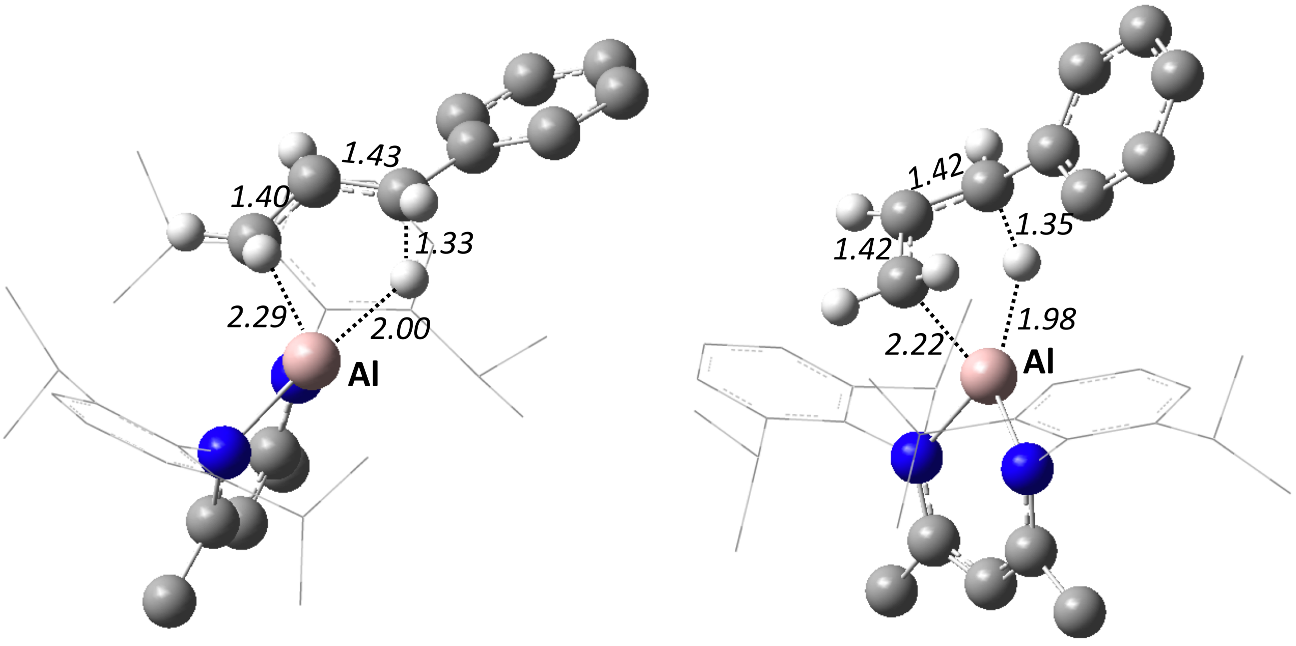

Table S3: Relative free energies (enthalpies) kcal mol^{-1} of **Int-1**, **TS-1** and **2** for a range of alkenes. M06L, Al (SDDAll), C H N (6-31G**) + ΔE_{solv} (PCM, benzene).

Substrate/Product	Int-1	TS-1	2
**Norbornene/2a**	5.6 (-5.3)	18.3 (2.1)	-1.1 (-17.1)
**Ethylene/2b**	7.0 (-3.8)	18.0 (4.2)	0.0 (-14.5)
**Propylene/2c**	5.5 (-5.1)	21.4 (6.3)	4.7 (-10.1)
**Butene/2d'**	5.6 (0.0)	20.5 (5.4)	3.3 (-11.3)
**Allylbenzene/2f**	4.9 (-8.0)	19.2 (4.3)	2.0 (-13.4)

Figure S20: Calculated pathways for the reaction of **1** with propylene; Gibbs free energies in kcal mol^{-1}. M06L, Al (SDDAll), C H N (6-31G**) + ΔE_{solv} (PCM, benzene).

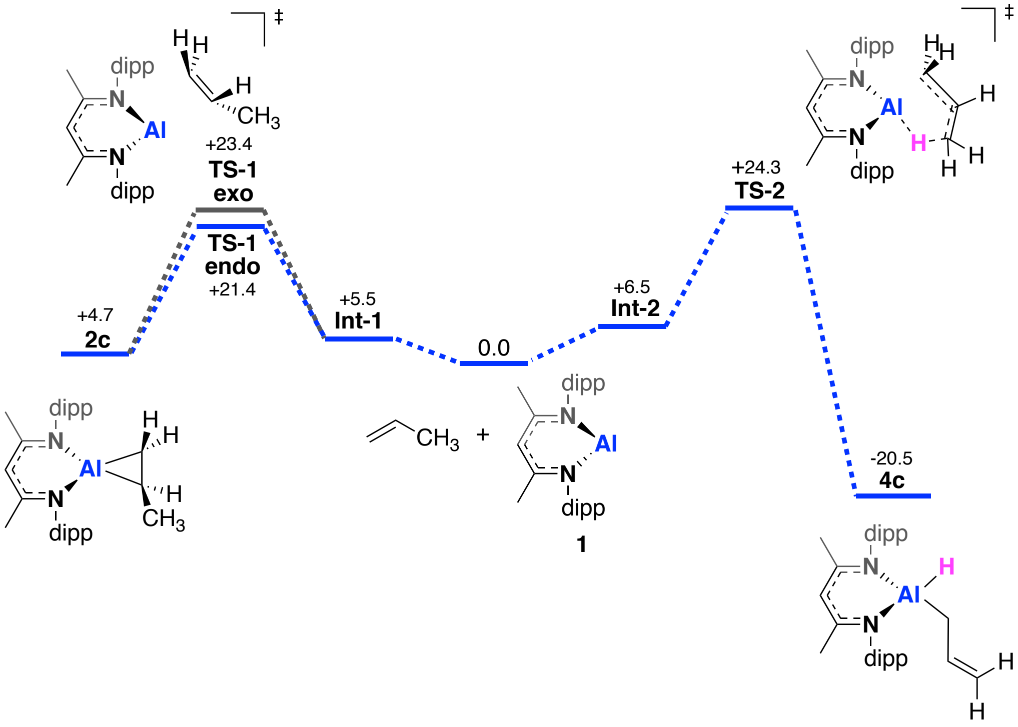

Table S4: Relative free energies and enthalpies (kcal mol^{-1}) of **TS-1$_{endo}$**, **TS-1c$_{exo}$** and **TS-2** using specified density functionals. All values single point corrected for solvent (except entry 1). Dispersion single point correction using GD3BJ for B3PW91, D2 single point correction for ωB97X.

**Functional**		**TS-1$_{exo}$**	**TS-1c$_{endo}$**	**TS-2**	$\Delta\Delta G^{\ddagger}$ **TS-1c$_{exo}$/TS-2**	$\Delta\Delta G^{\ddagger}$ **TS-1c$_{endo}$/TS-2**
**ωB97X**	ΔH	21.5	18.3	20.6		
	ΔG	36.6	33.8	35.9	−0.7	2.1
**ωB97XD**	ΔH	14.2	11.5	14.1		
	ΔG	29.2	27.1	29.4	0.2	2.3
**M062X**	ΔH	12.2	10.2	13.9		
	ΔG	29.3	26.2	30.0	0.7	3.8
**M06L**	ΔH	7.8	6.3	8.9		
	ΔG	23.4	21.4	24.3	0.9	2.9
**B3PW91**	ΔH	12.1	9.6	11.3		
	ΔG	26.6	24.3	25.3	−1.3	1.0

Figure S21: Calculated pathways for the reaction of **1** with allylbenzene; Gibbs free energies in kcal mol^{-1}. M06L, Al (SDDAll), C H N (6-31G**) + ΔE$_{solv}$ (PCM, benzene).

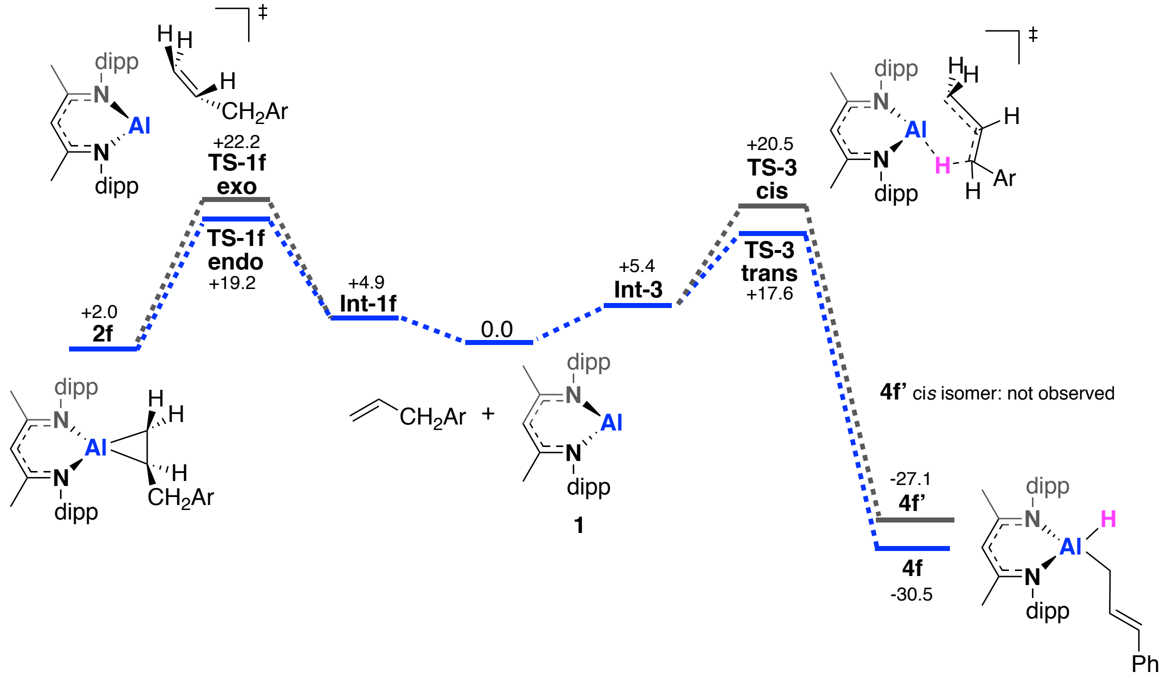

Table S5: Relative free energies and enthalpies (kcal mol^{-1}) of **TS-1f$_{exo}$**, **TS-1f$_{endo}$**, **TS-3$_{trans}$** and **TS-3$_{cis}$** using specified density functionals. All values single point corrected for solvent (except entry 1). Dispersion single point correction using GD3BJ for B3PW91, D2 single point correction for ωB97X.

**Functional**		**TS-1f$_{endo}$**	**TS-1f$_{exo}$**	**TS-3$_{trans}$**	**TS-3$_{cis}$**	**$\Delta\Delta G^{\ddagger}$ TS-1f$_{endo}$/TS-3$_{trans}$**	**$\Delta\Delta G^{\ddagger}$ TS-1f$_{exo}$/TS-3$_{trans}$**	**$\Delta\Delta G^{\ddagger}$ TS-3$_{trans}$/TS-3$_{cis}$**
**ωB97X**	ΔH	17.5	22.2	15.3	18.3			
	ΔG	33.3	35.6	31.5	34.6	-1.8	-1.0	3.1
**ωB97XD**	ΔH	8.8	10.4	6.8	9.4			
	ΔG	24.6	25.9	23.1	25.7	-1.5	-2.8	2.6
**M062X**	ΔH	8.8	10.6	7.0	10.1			
	ΔG	26.1	30.0	26.0	28	-0.1	-4.0	2.0
**M06L**	ΔH	4.3	4.9	0.2	3.3			
	ΔG	**19.2**	**22.2**	**17.6**	**20.5**	**-1.6**	**-4.6**	**2.9**
**B3PW91**	ΔH	7.7	8.9	4.8	-0.8			
	ΔG	22.2	23.4	19.7	20.8	-2.5	-3.7	1.1

7. Multinuclear NMR Data

Figure S22: ^{1}H NMR spectrum of compound **2b**.

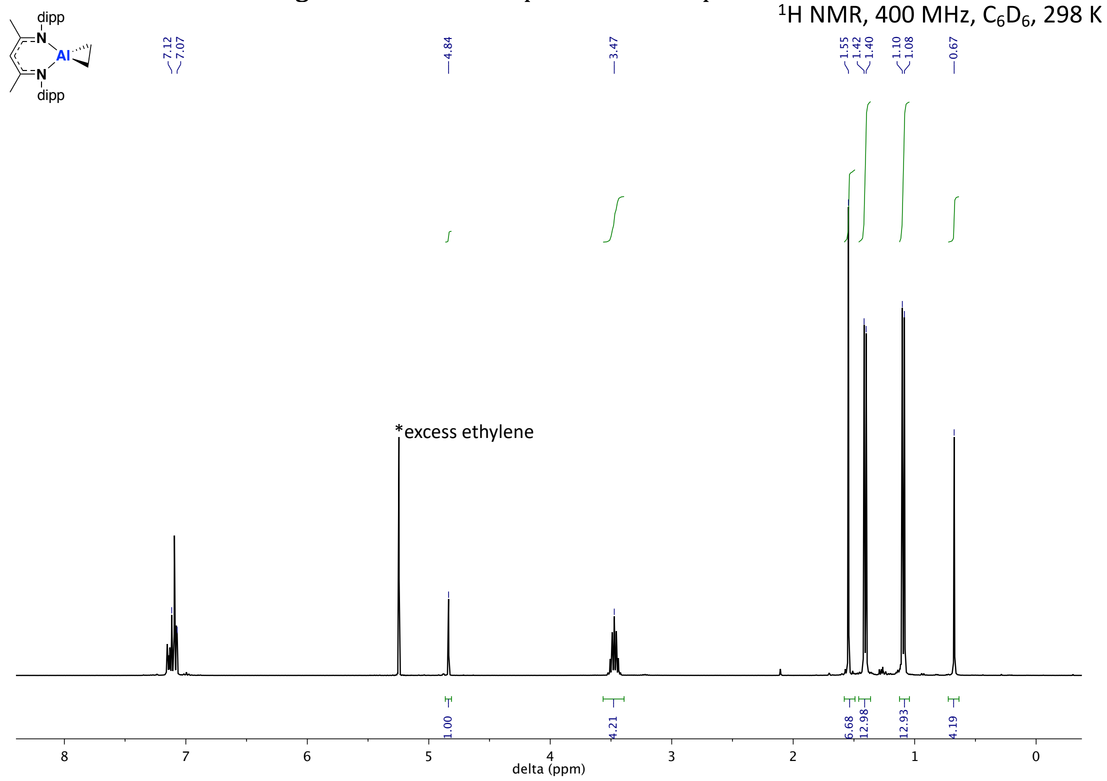

Figure S23: ^{1}H NMR spectrum of compound **2c**.

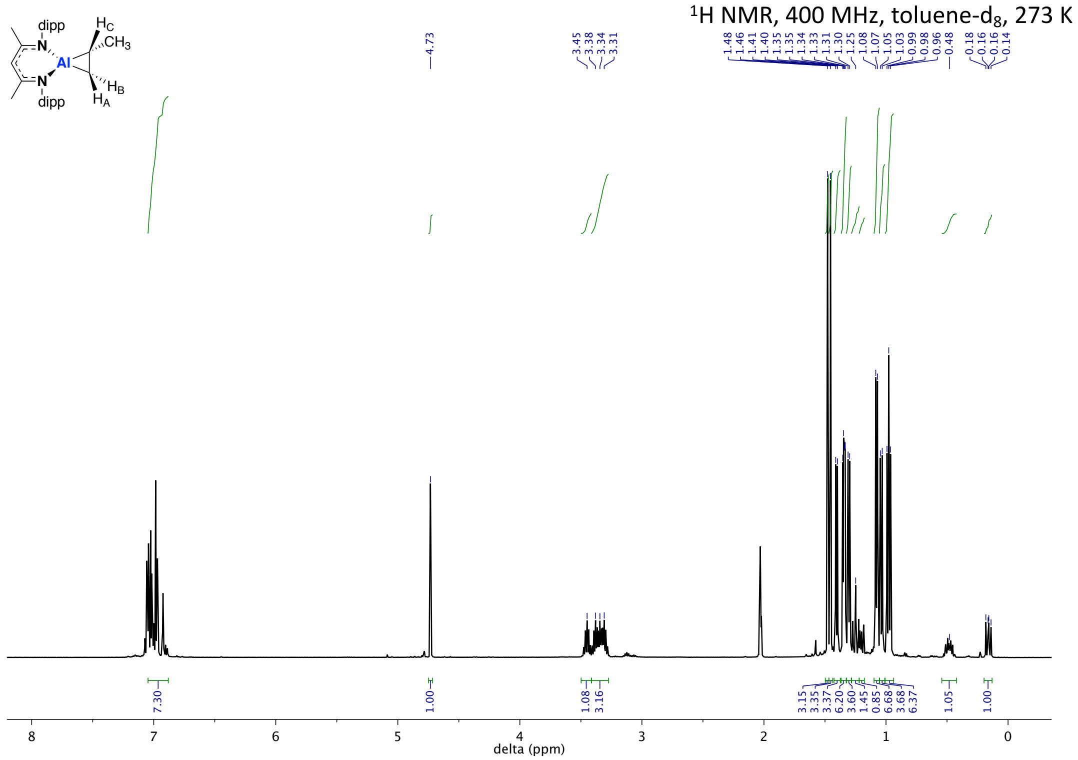

Figure S24: $^{13}C\{^{1}H\}$ NMR spectrum of compound **2c**.

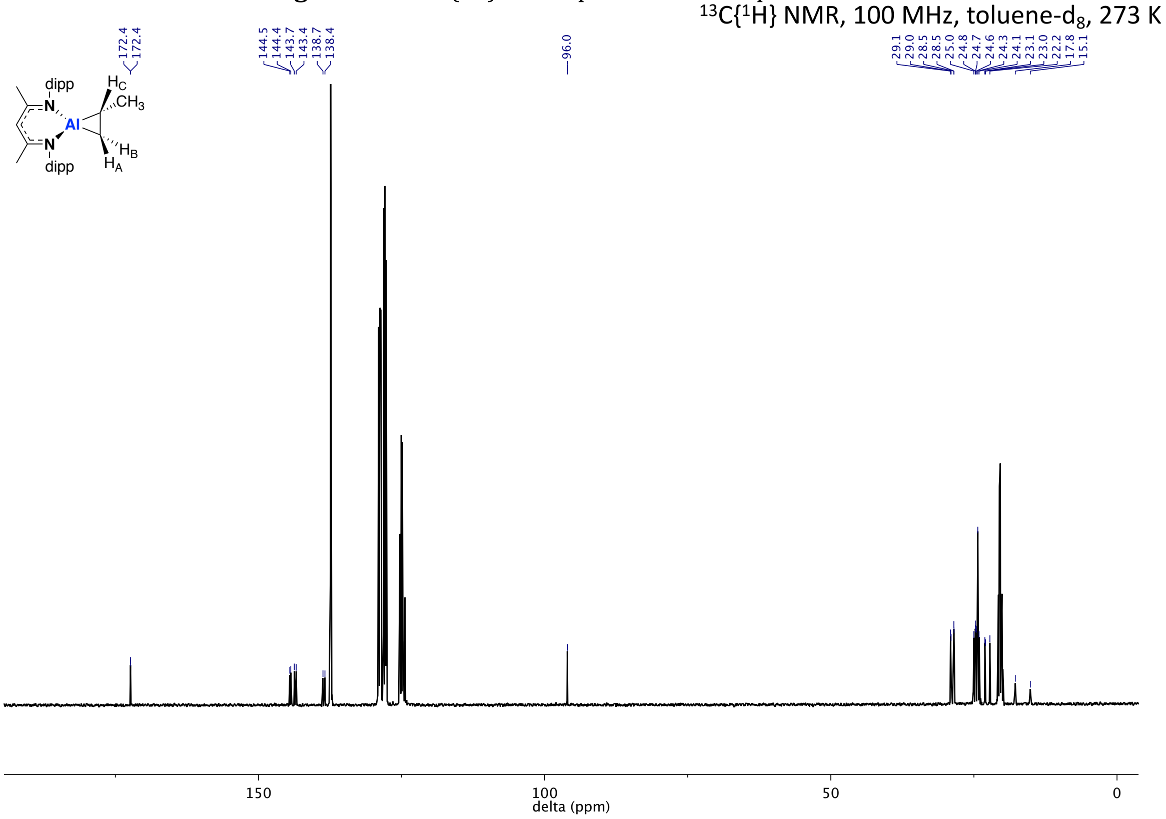

Figure S25: ^{1}H NMR spectrum of compound **2d**.

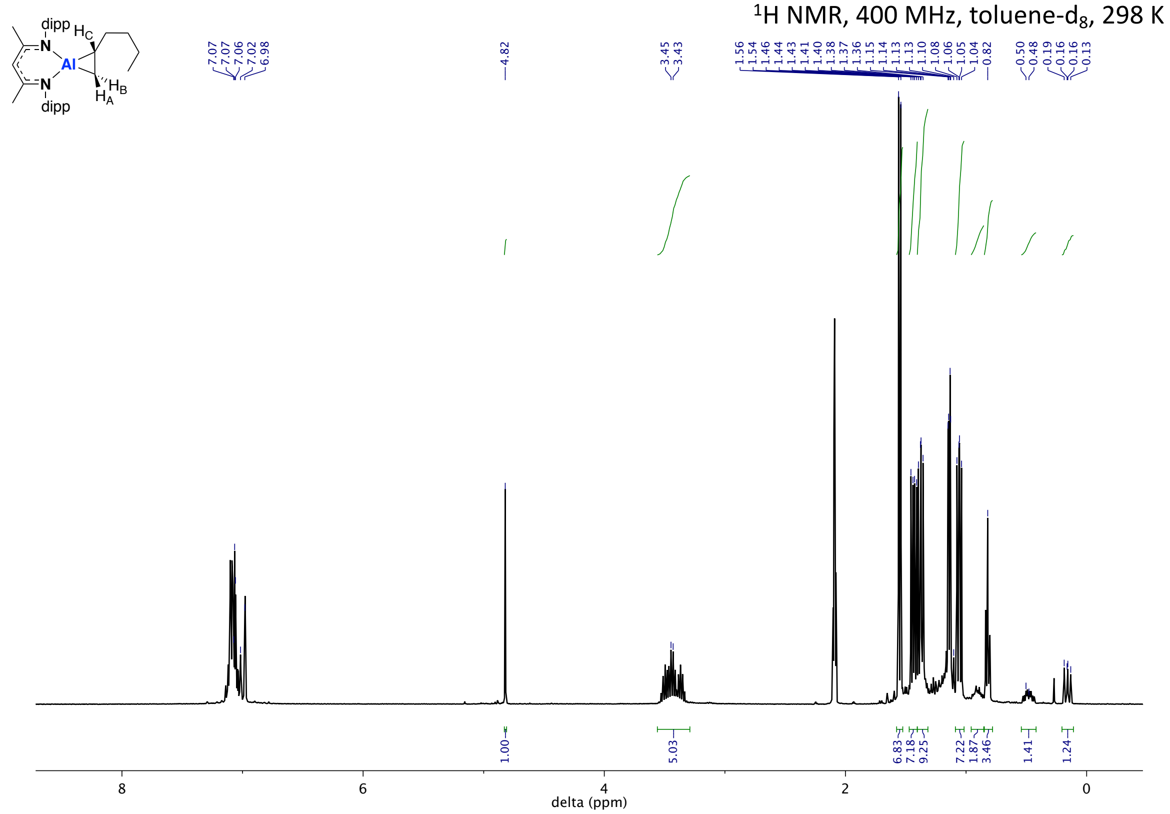

Figure S26: $^{13}C\{^1H\}$ NMR spectrum of compound **2d**.

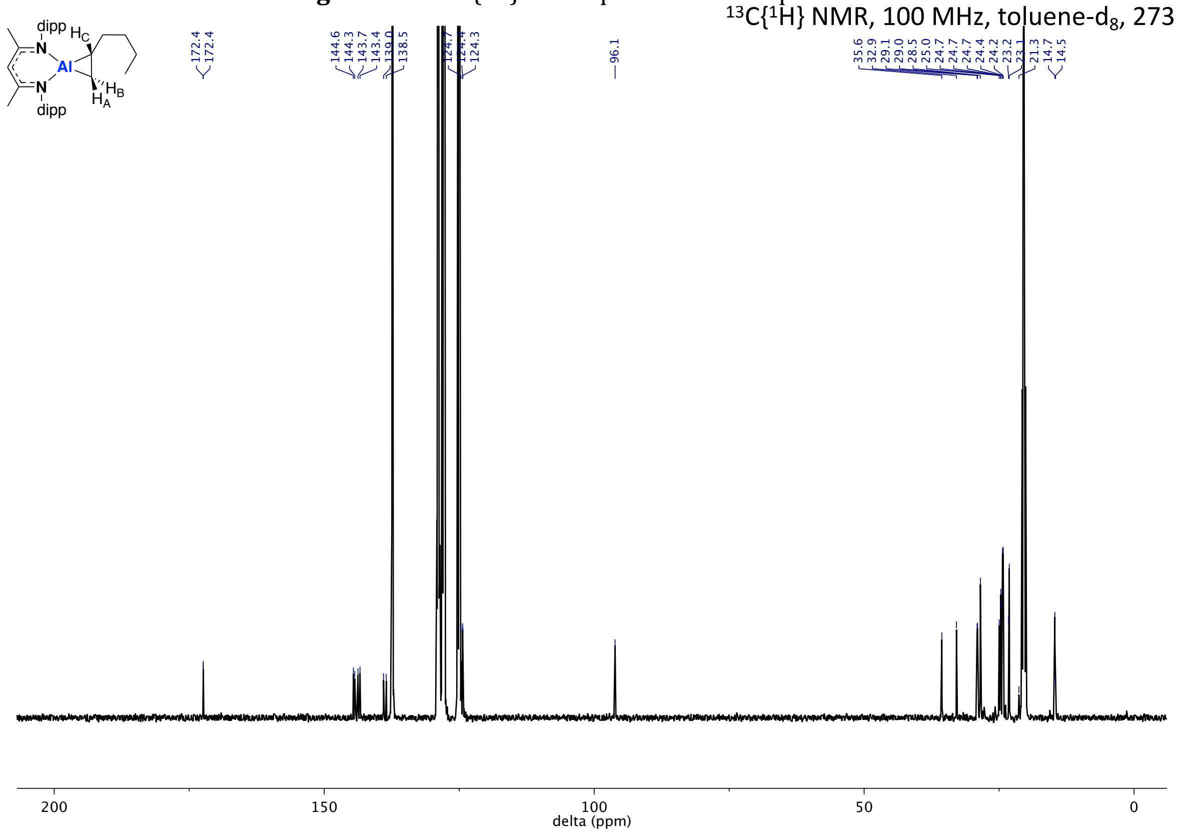

Figure S27: ^1H NMR spectrum of compound **2e**.

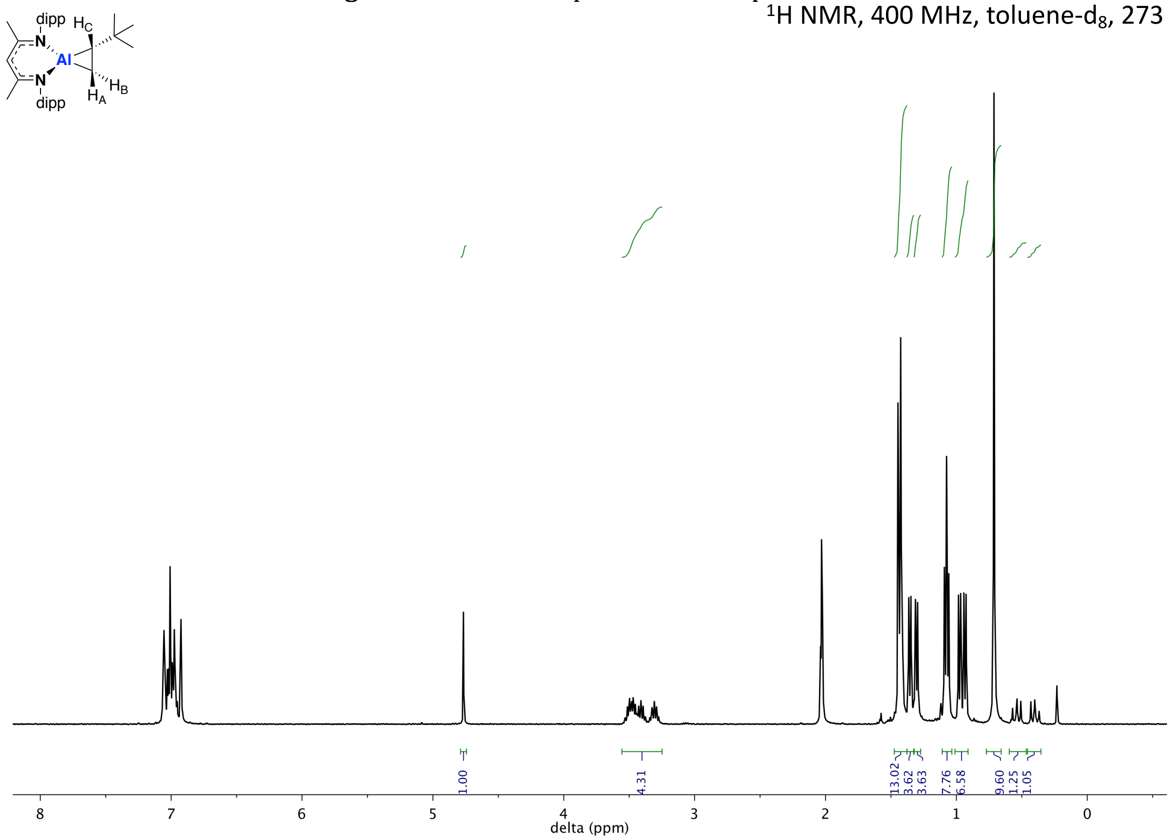

Figure S28: $^{13}C\{^1H\}$ NMR spectrum of compound **2e**.

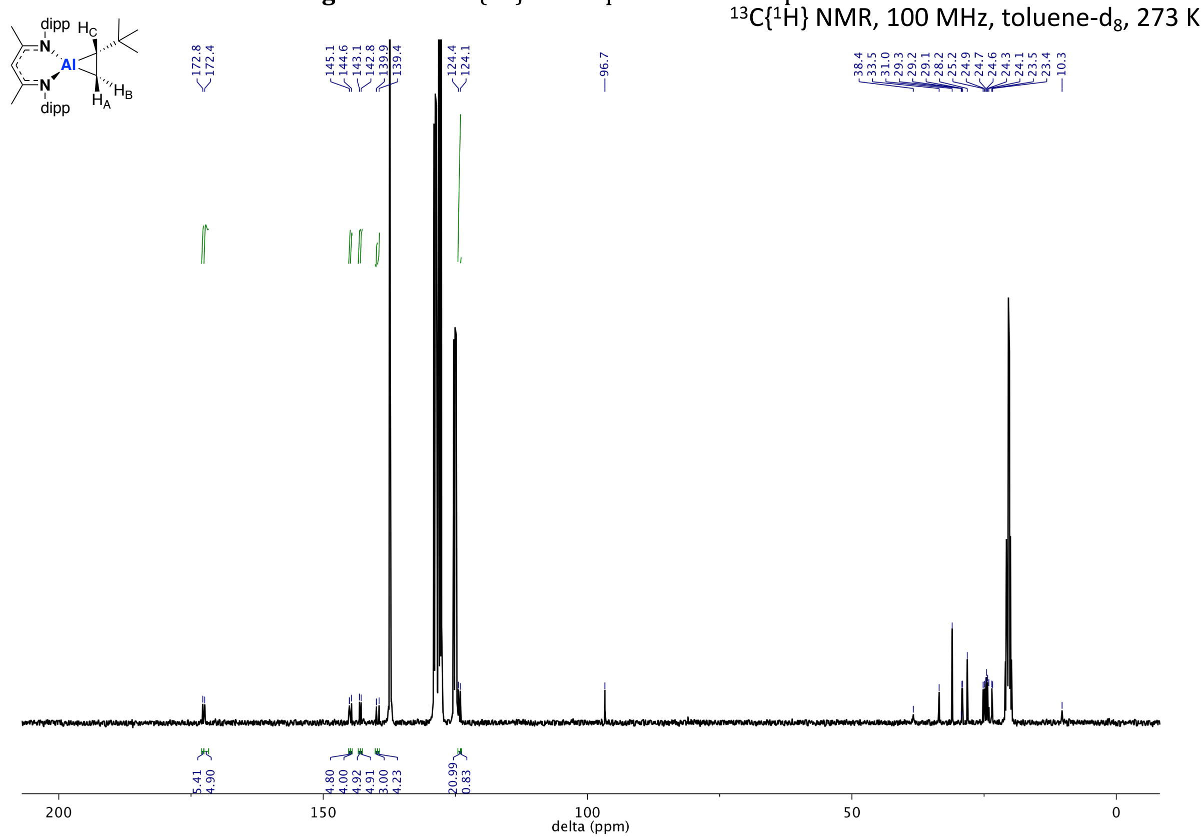

Figure S29: ^1H NMR spectrum of compound **2f**.

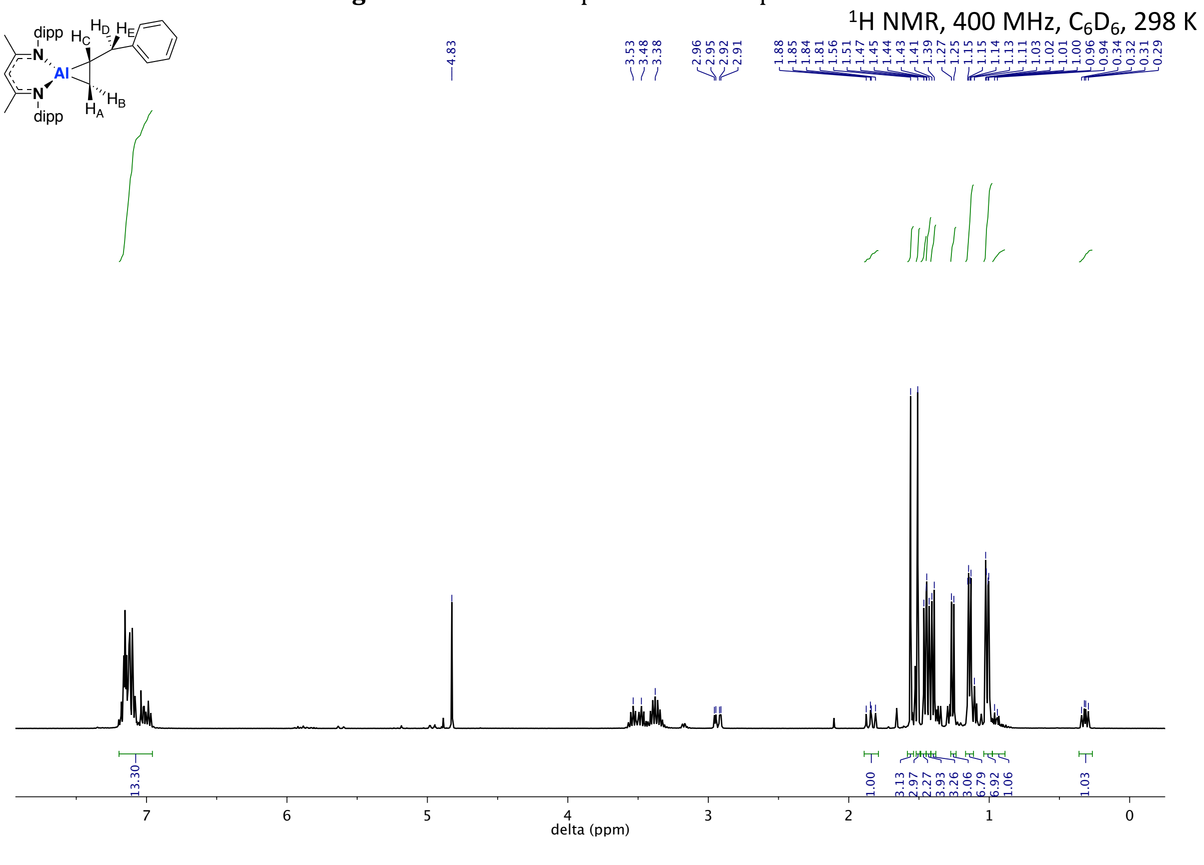

Figure S30: $^{13}C\{^1H\}$ NMR spectrum of compound **2f**.

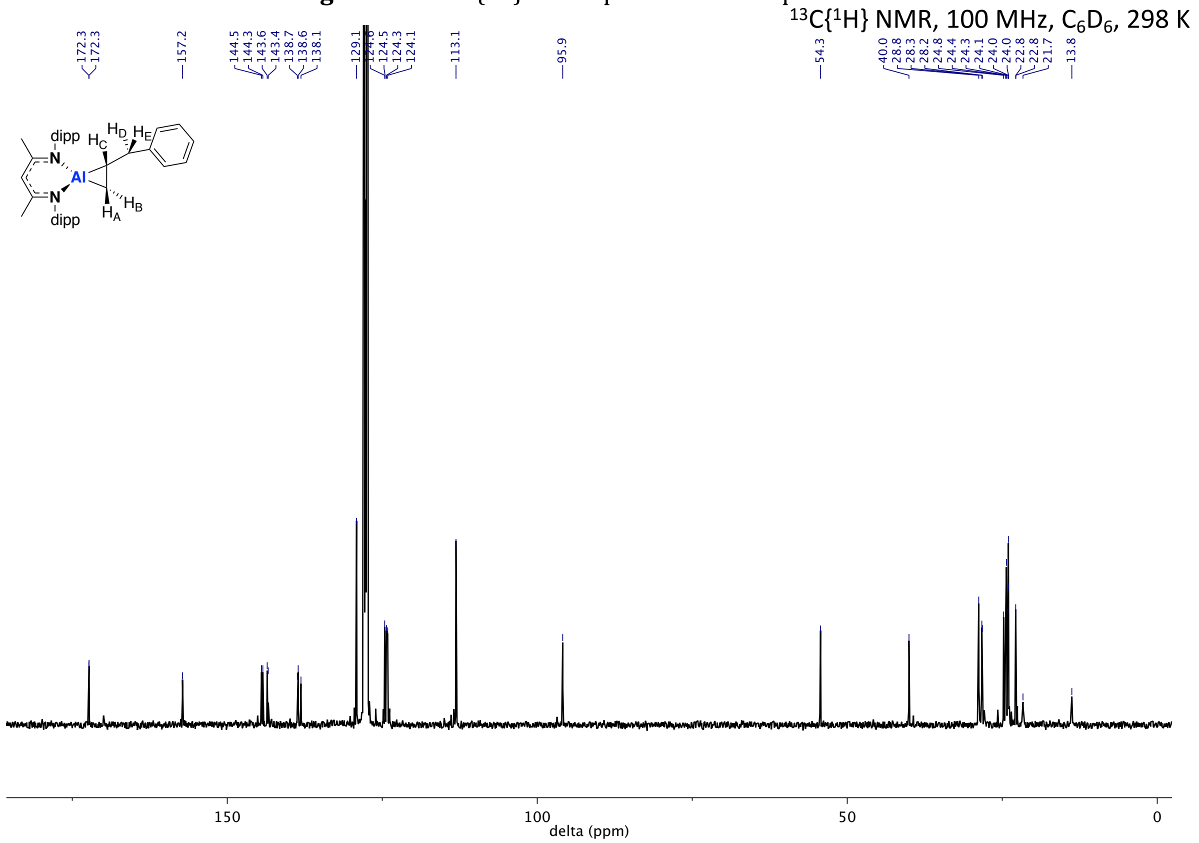

Figure S31: ^1H NMR spectrum of compound **2g**.

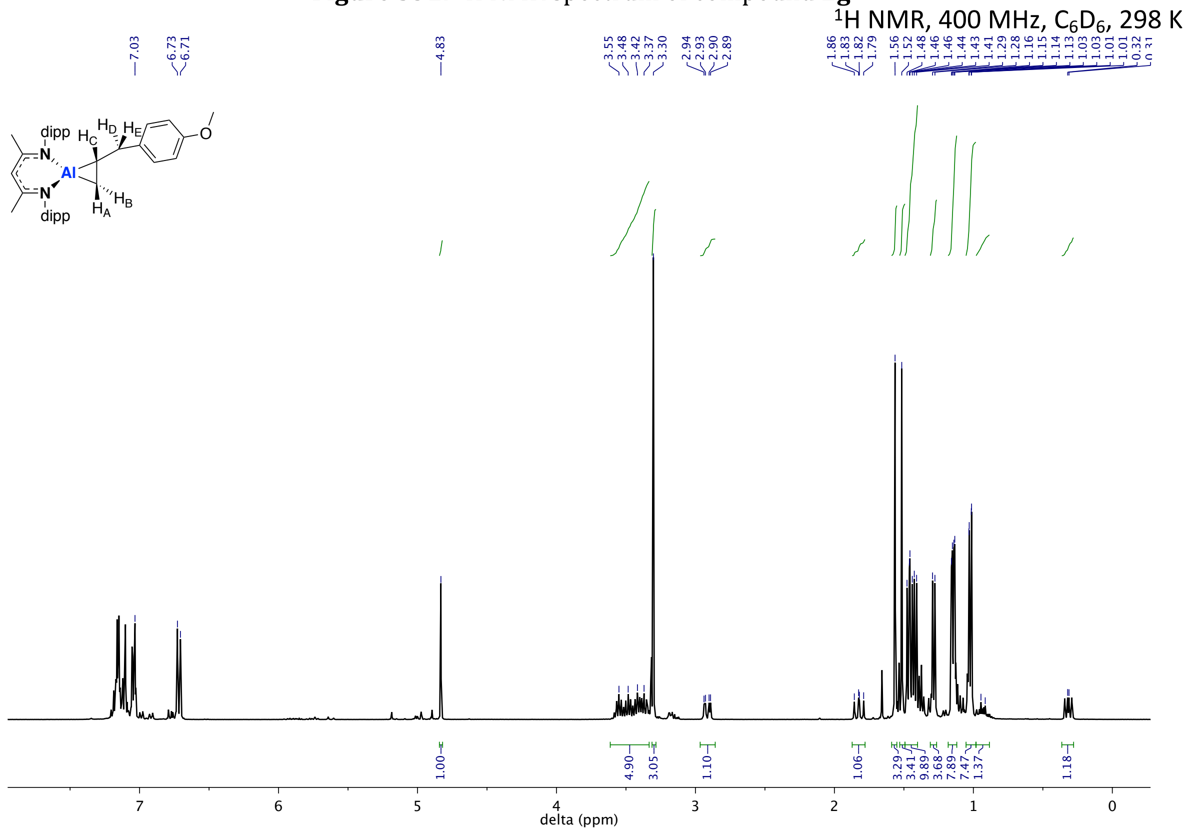

Figure S32: $^{13}C\{^{1}H\}$ NMR spectrum of compound **2g**.

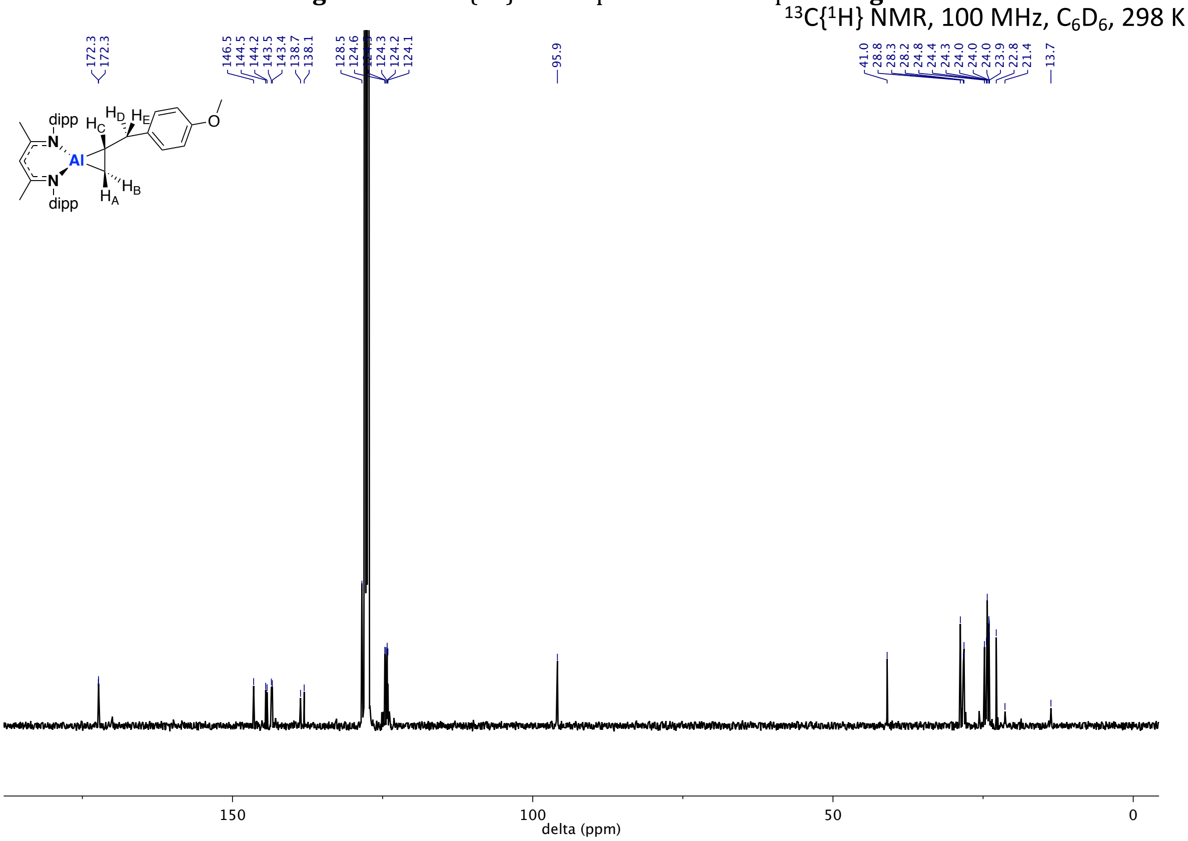

Figure S33: ^{1}H NMR spectrum of compound **3**.

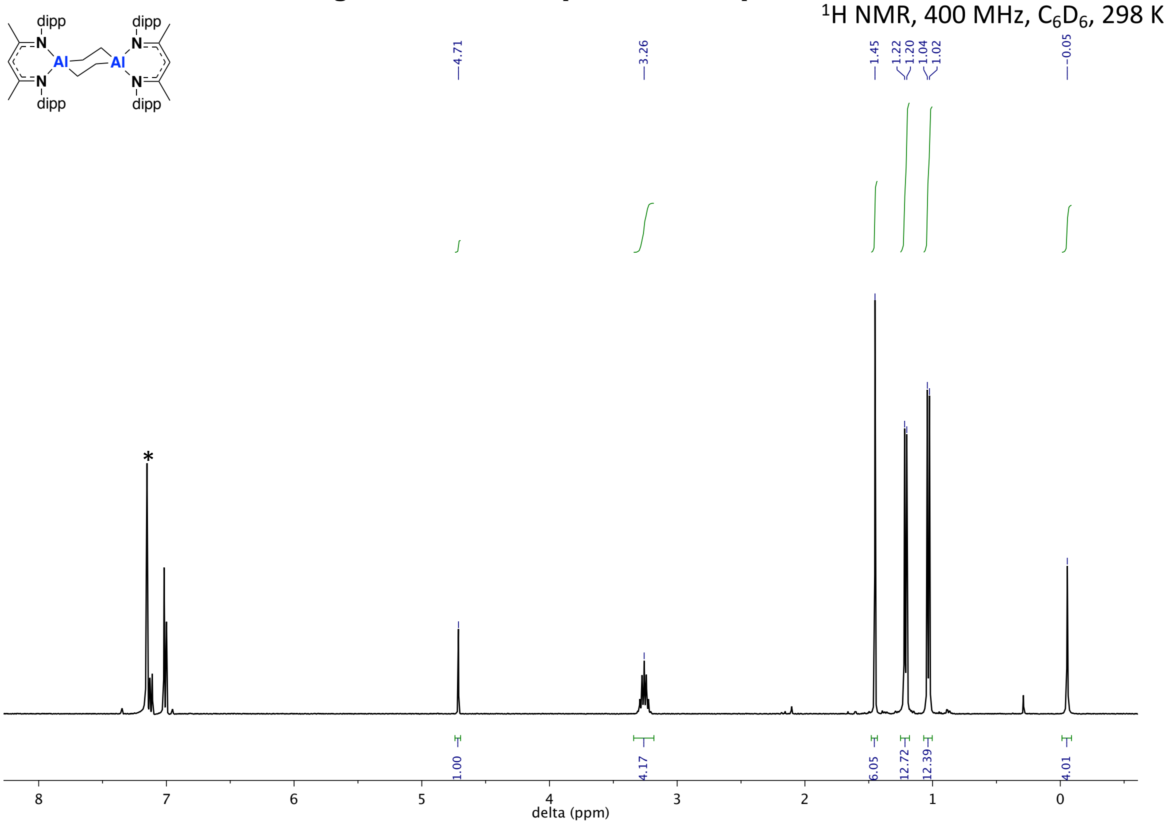

Figure S34: $^{13}C\{^{1}H\}$ NMR spectrum of compound **3**.

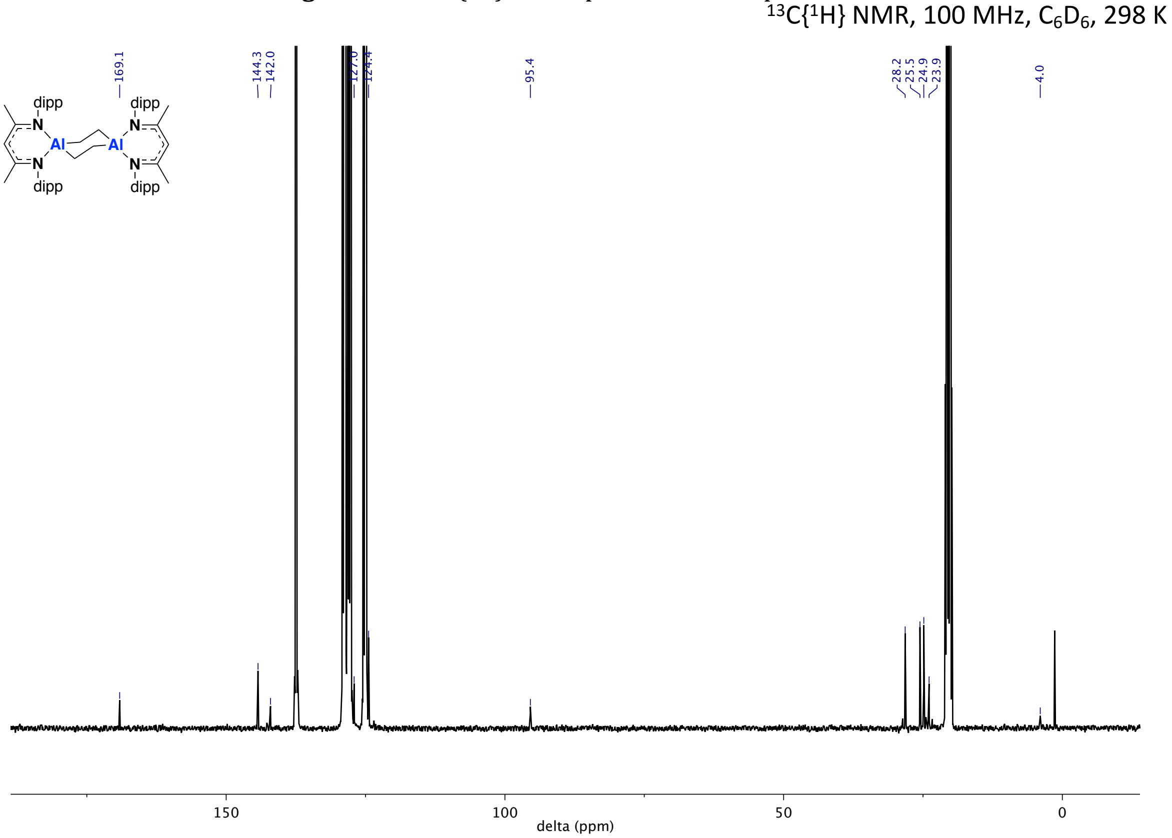

Figure S35: ^{1}H NMR spectrum of compound **4c**.

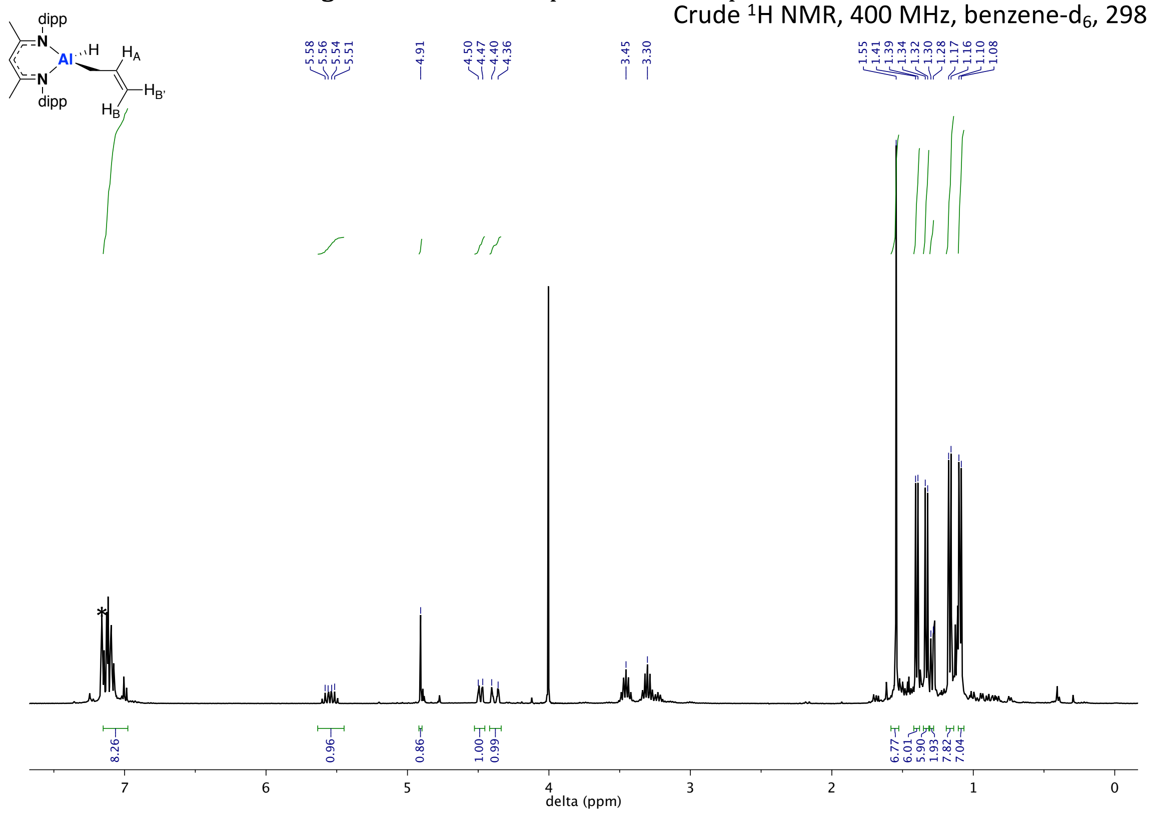

Figure S36: ^{1}H NMR spectrum of compound **4f**.

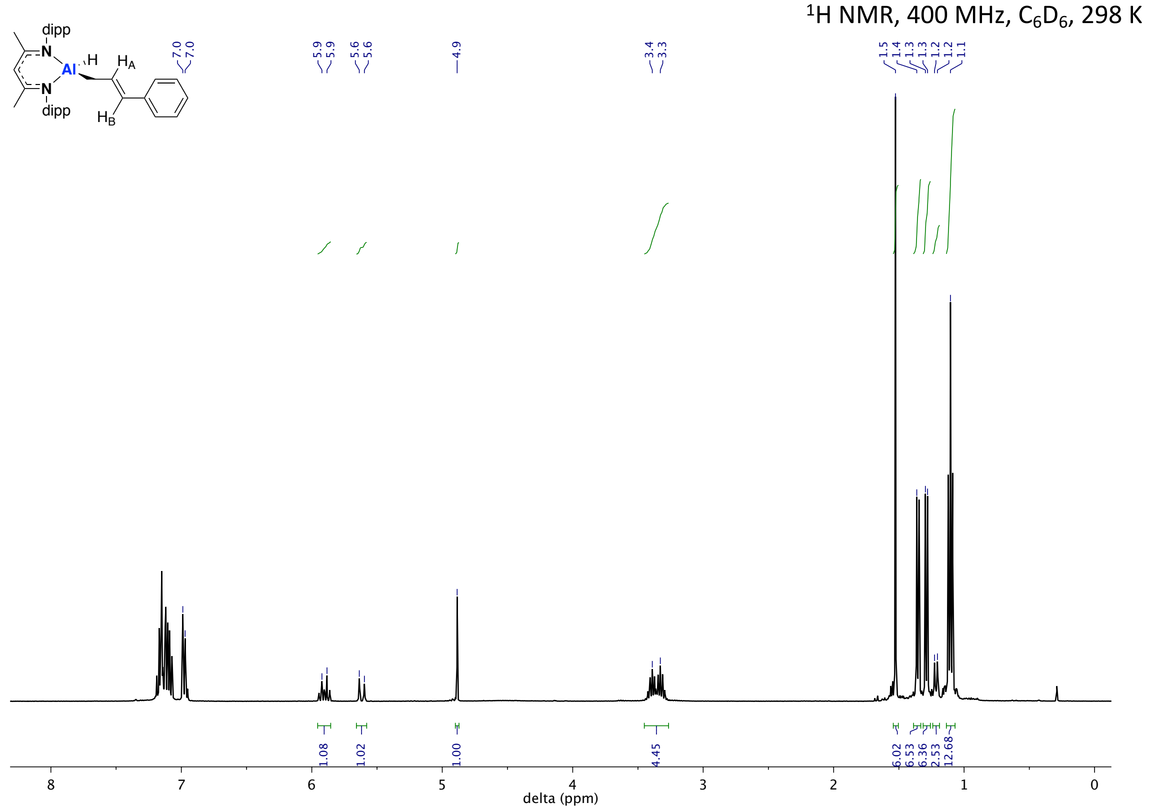

Figure S37: ^{13}C{^{1}H} NMR spectrum of compound **4f**.

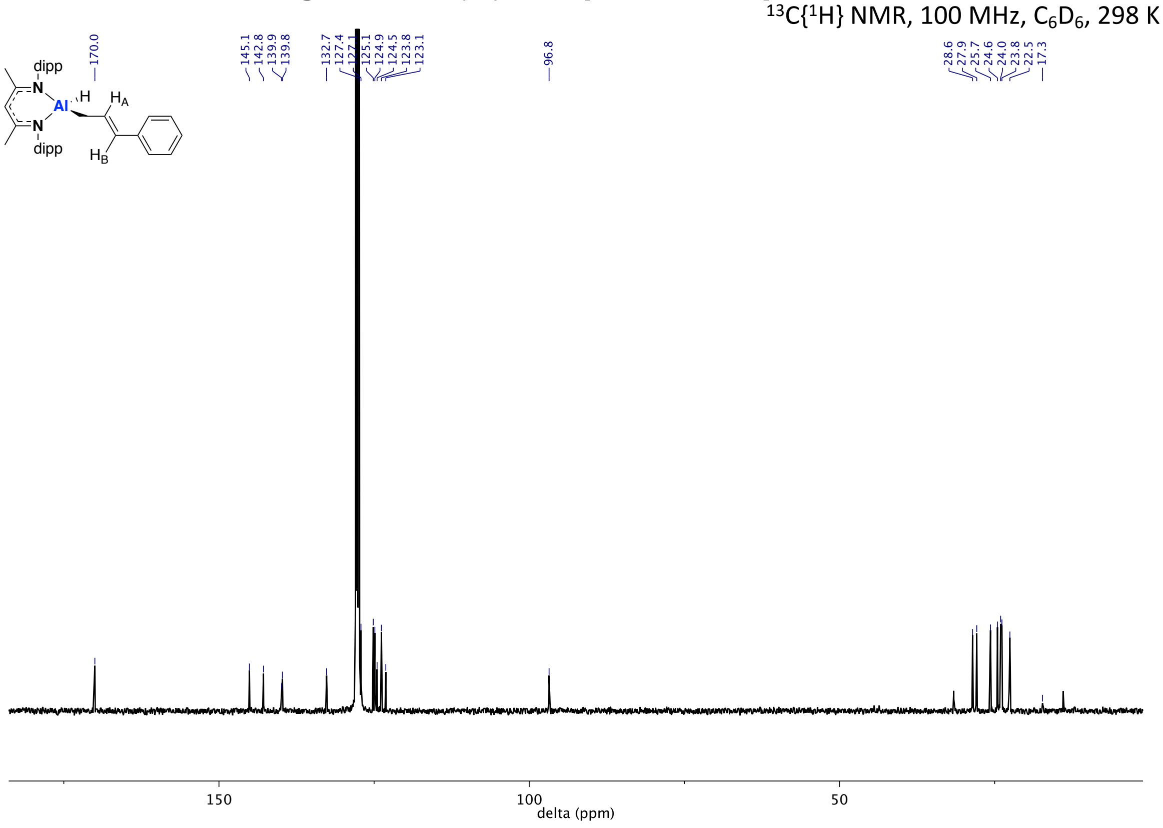

Figure S38: ^{1}H NMR spectrum of compound **4g**.

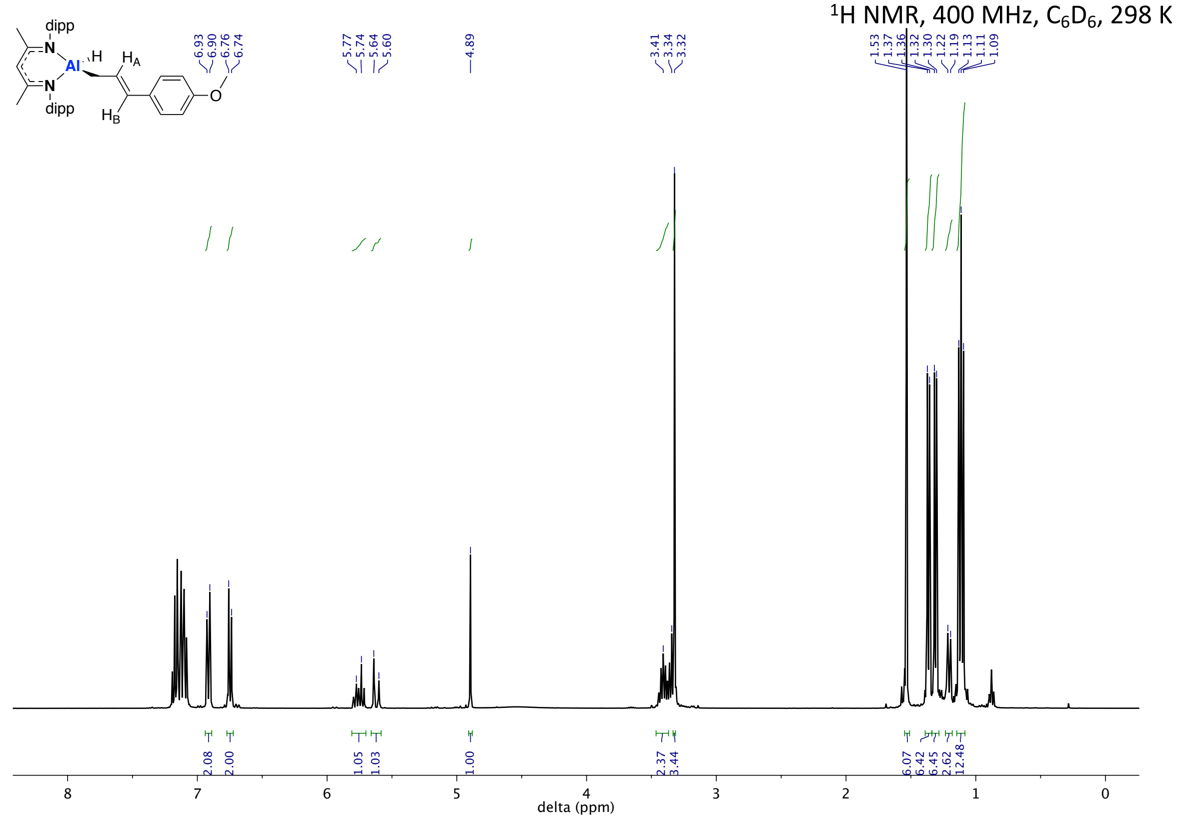

Figure S39: ^{13}C{^{1}H} NMR spectrum of compound **4g**.

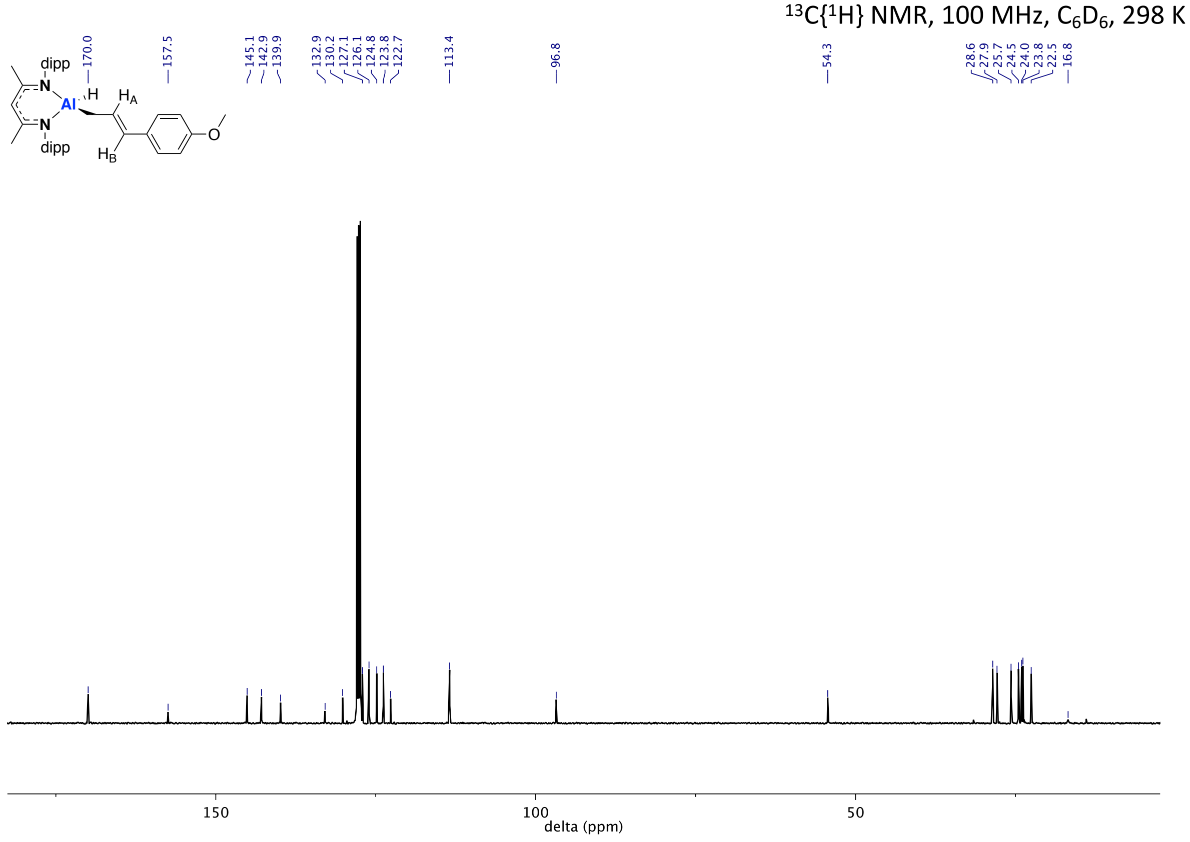

8. References

(1) Cui, C.; Roesky, H. W.; Schmidt, H. G.; Noltemeyer, M.; Hao, H.; Cimpoesu, F. Synthesis and Structure of a Monomeric Aluminum(I) Compound [{HC(CMeNAr)$_2$}Al] (Ar=2,6-*I*Pr$_2$C$_6$H$_3$): A Stable Aluminum Analogue of a Carbene. *Angew. Chem. Int. Ed.* **2000**, *39*, 4274–4276.

(2) SHELXTL v5.1, Bruker AXS, Madison, WI, 1998.

(3) Sheldrick, G. M. Shelx-2013. *Act. Cryst.* **2015**, *C17*, 3.

(4) Frisch, M. J.; Trucks, G. W.; Schegel, H. B.; Scuseria, G. E.; Robb, M. A.; Cheeseman, J. R.; Scalmani, G.; Barone, V.; Mennucci, B.; Petersson, G. A.; et al. *Gaussian Inc.* Wallingford CT January 1, 2009.

(5) Hehre, W. J.; Ditchfield, R.; Pople, J. A. Self-Consistent Molecular Orbital Methods. XII. Further Extensions of Gaussian-Type Basis Sets for Use in Molecular Orbital Studies of Organic Molecules. *J. Chem. Phys.* **1972**, *56* (5), 2257–2261.

(6) Hariharan, P. C.; Pople, J. A. The Influence of Polarization Functions on Molecular Orbital Hydrogenation Energies. *Theoret. chim. Acta* **1973**, *28* (3), 213–222.

(7) Clark, T.; Chandrasekhar, J.; Spitznagel, G. W.; Rague Schleyer, von, P. Efficient Diffuse Function-Augmented Basis Sets for Anion Calculations. *J. Comput. Chem.* **1983**, *4* (3), 294–301.

(8) Fukui, K. The Path of Chemical Reactions - the IRC Approach. *Acc. Chem. Res.* **2002**, *14* (12), 363–368.

(9) Hratchian, H. P.; Schlegel, H. B. *Theory and Applications of Computational Chemistry; Theory and Applications of Computational Chemistry*; 2005; pp 195–249.

(10) Tomasi, J.; Mennucci, B.; Cammi, R. Quantum Mechanical Continuum Solvation Models. *Chem. Rev.* **2005**, *105* (8), 2999–3094.

(11) Chai, J.-D.; Head-Gordon, M. Systematic Optimization of Long-Range Corrected Hybrid Density Functionals. *J. Chem. Phys.* **2008**, *128* (8), 84106–841116.

(12) Dennington, R.; Keith, T.; Millam, J. GaussView, Version 5.0.9 *Semichem Inc., Shawnee Mission, KS*, 2009.

(13) Glendening, E. D.; Badenhoop, J. K.; Reed, A. E.; Carpenter, J. E.; Bohmann, J. A.; Morales, C. M.; Landis, C. R.; Weinhold, F. NBO Version 6.0, Theoretical Chemistry Institute, University of Wisconsin, Madison (2013).

9. XYZ Coordinates

1

SCF (wB97X) = -1241.24017203
H(0 K)= -1240.602498
H(298 K)= -1240.566154
G(298 K)= -1240.668856
Lowest Frequency = 18.3028cm-1
PCM (Benzene) Energy = -1241.24348553

Al	-0.000669	0.029384	-1.011722
N	-1.404006	-0.002257	0.454574
C	-1.253342	-0.036403	1.783811
C	-0.002393	0.029119	2.407571
H	-0.002663	0.032317	3.491793
C	1.249247	0.082562	1.784337
N	1.400868	0.043069	0.455189
C	-2.465534	-0.143354	2.664183
H	-3.067183	-1.016585	2.391459
H	-3.122855	0.723879	2.538048
H	-2.189978	-0.219525	3.716934
C	2.462180	0.176035	2.665049
H	3.080004	1.035626	2.385332
H	3.103360	-0.704361	2.546788
H	2.187126	0.265767	3.716869
C	-2.734748	-0.054401	-0.077136
C	-3.471501	1.141181	-0.209214
C	-4.730476	1.079633	-0.807387
H	-5.310674	1.991915	-0.921841
C	-5.253102	-0.125812	-1.263178
H	-6.234163	-0.152321	-1.729242
C	-4.517321	-1.293003	-1.119605
H	-4.926569	-2.236800	-1.475394
C	-3.251354	-1.283703	-0.526623
C	-2.892222	2.468920	0.243032
H	-2.160010	2.263503	1.034699
C	-2.137235	3.142463	-0.903573
H	-1.353868	2.489857	-1.311549
H	-1.665460	4.074422	-0.573228
H	-2.818296	3.380489	-1.728279
C	-3.938452	3.414353	0.824152
H	-4.528059	2.937934	1.614100
H	-4.637597	3.769173	0.059433
H	-3.457314	4.300513	1.248842
C	-2.488662	-2.587568	-0.385107
H	-1.547538	-2.377301	0.140907
C	-3.266833	-3.603389	0.450115
H	-4.207533	-3.882291	-0.037621
H	-3.516881	-3.213030	1.441889
H	-2.685574	-4.520862	0.586706
C	-2.134480	-3.171135	-1.752037
H	-3.037841	-3.406065	-2.326407
H	-1.558583	-4.096488	-1.644562
H	-1.539069	-2.465368	-2.341635
C	2.733546	0.058310	-0.074203
C	3.436861	-1.157929	-0.199899
C	4.698164	-1.134362	-0.795990
H	5.252619	-2.063173	-0.905349
C	5.255140	0.053885	-1.256110
H	6.237262	0.050866	-1.720670
C	4.552119	1.241837	-1.118581
H	4.988143	2.172399	-1.477484
C	3.285526	1.270658	-0.527476
C	2.821148	-2.467672	0.257011
H	2.087129	-2.239212	1.040745
C	3.839380	-3.433606	0.854136
H	3.333507	-4.304858	1.280960
H	4.433368	-2.965605	1.645800
H	4.536491	-3.810678	0.098267
C	2.060461	-3.131789	-0.891379
H	1.294906	-2.464910	-1.310097
H	1.565514	-4.050787	-0.558383
H	2.742568	-3.390693	-1.708912
C	2.559725	2.595803	-0.391694
H	1.615280	2.414354	0.138580
C	2.216737	3.180252	-1.761110
H	1.667430	4.122165	-1.657428
H	1.600043	2.487761	-2.344376
H	3.124361	3.386601	-2.339800
C	3.368831	3.595055	0.434060
H	2.813520	4.528893	0.567429
H	4.314651	3.845128	-0.059364
H	3.612641	3.204025	1.427154

Norbornene

SCF (wB97X) = -272.698707246
H(0 K)= -272.545312
H(298 K)= -272.539144
G(298 K)= -272.573837
Lowest Frequency = 251.7257cm-1
PCM (Benzene) Energy = -272.699087584

C	1.271839	-0.669642	-0.498914
H	1.907593	-1.327340	-1.082940
C	1.271879	0.669589	-0.498901
H	1.907669	1.327261	-1.082917
C	0.085699	1.120088	0.322302
H	0.111809	2.148560	0.690325
C	-1.177225	0.773310	-0.517390
H	-2.076750	1.170363	-0.034619
H	-1.130523	1.202461	-1.522084
C	-1.177265	-0.773269	-0.517373
H	-1.130593	-1.202442	-1.522058
H	-2.076805	-1.170268	-0.034585
C	0.085652	-1.120089	0.322309
H	0.111724	-2.148559	0.690339
C	0.033805	0.000004	1.370979
H	0.899973	-0.000012	2.038730
H	-0.890406	0.000027	1.961741

Int-1a
SCF (wB97X) = -1513.93708345
H(0 K)= -1513.144899
H(298 K)= -1513.103173
G(298 K)= -1513.214822
Lowest Frequency = -245.8546cm-1
PCM (Benzene) Energy = -1513.93951567

N	1.299152	-0.770465	-0.789441
Al	-0.077712	-0.889021	0.612415
N	-1.533496	-0.786448	-0.708068
C	1.088938	-1.500216	-1.895955
C	-0.177886	-1.956221	-2.297077
C	-1.426318	-1.512284	-1.818106
C	-0.528022	0.310288	2.255859
C	-0.263986	1.421537	1.377797
H	-0.201080	-2.577366	-3.185347
H	-1.083823	1.972615	0.922219
H	-1.555480	0.047146	2.522825
C	2.251599	-1.830108	-2.786073
H	1.922670	-2.337927	-3.693175
H	2.800990	-0.926215	-3.066486
H	2.970096	-2.473393	-2.266534
C	-2.651268	-1.918563	-2.583957
H	-2.994323	-2.893548	-2.215341
H	-3.476595	-1.216325	-2.449413
H	-2.441711	-2.028727	-3.649732
C	-2.757093	-0.146478	-0.343046
C	-3.693534	-0.811547	0.468663
C	-2.945657	1.196429	-0.738073
C	-4.812717	-0.095033	0.905086
C	-4.084029	1.863175	-0.285105
C	-5.008078	1.229227	0.539870
H	-5.542953	-0.593530	1.540152
H	-4.246926	2.897674	-0.577037
H	-5.883285	1.768518	0.891617
C	2.615527	-0.261169	-0.541633
C	3.580642	-1.092664	0.061573
C	2.896464	1.091146	-0.842950
C	4.835753	-0.550020	0.351922
C	4.169674	1.578256	-0.539495
C	5.134372	0.769989	0.051580
H	5.584823	-1.177618	0.830309
H	4.410395	2.613714	-0.760292
H	6.115590	1.175076	0.283339
C	1.835071	1.996544	-1.441626
H	0.921538	1.814707	-0.853437
C	3.289270	-2.526803	0.464910
H	2.341831	-2.833514	0.003016
C	-3.544424	-2.270114	0.856995
H	-2.651350	-2.667170	0.357211
C	-1.951680	1.870286	-1.664181
H	-0.977227	1.399779	-1.477480
C	-1.797837	3.366810	-1.419057
H	-2.708216	3.922458	-1.670577
H	-1.542510	3.593854	-0.378532
H	-0.999022	3.766693	-2.053234
C	-2.314190	1.621341	-3.130073
H	-3.317718	2.001313	-3.354919
H	-1.608362	2.129400	-3.796016
H	-2.295752	0.558011	-3.383947
C	-4.749832	-3.086198	0.388797
H	-5.663364	-2.782117	0.911891
H	-4.934078	-2.964466	-0.683427
H	-4.599320	-4.151697	0.589568
C	-3.336093	-2.440828	2.360664
H	-2.409233	-1.962682	2.692758
H	-4.167840	-2.006015	2.927185
H	-3.272534	-3.501388	2.624707
C	3.108214	-2.631718	1.980070
H	4.006666	-2.286933	2.505594
H	2.261759	-2.026446	2.324707
H	2.916558	-3.667432	2.279312
C	4.374342	-3.493623	-0.006461
H	4.562589	-3.412773	-1.082281
H	5.326506	-3.311164	0.503419
H	4.087283	-4.527372	0.208672
C	1.515076	1.672983	-2.903815
H	0.808596	2.410585	-3.302946
H	2.417933	1.718385	-3.524902
H	1.055871	0.689844	-3.033280
C	2.173947	3.479722	-1.334425
H	1.308683	4.083012	-1.625384
H	2.458052	3.774991	-0.320029
H	2.997487	3.756613	-2.003692
C	0.075910	1.678493	4.186369
H	-0.982777	1.650764	4.463570
H	0.650439	1.730569	5.117613
C	0.423499	2.856883	3.241739
H	1.206836	3.494379	3.673453
H	-0.437145	3.498212	3.026862
C	1.703833	0.971091	2.616147
H	2.116125	0.259161	1.891971
H	2.514801	1.303973	3.273516
C	0.490838	0.426739	3.373509
H	0.644762	-0.477069	3.970677
C	0.933792	2.132210	1.972602
H	1.509722	2.769732	1.294164

TS-1a
SCF (wB97X) = -1513.93708345
H(0 K)= -1513.144899
H(298 K)= -1513.103173
G(298 K)= -1513.214822
Lowest Frequency = -245.8546cm-1
PCM (Benzene) Energy = -1513.93951567

N	1.299152	-0.770465	-0.789441
Al	-0.077712	-0.889021	0.612415
N	-1.533496	-0.786448	-0.708068
C	1.088938	-1.500216	-1.895955
C	-0.177886	-1.956221	-2.297077

C	-1.426318	-1.512284	-1.818106
C	-0.528022	0.310288	2.255859
C	-0.263986	1.421537	1.377797
H	-0.201080	-2.577366	-3.185347
H	-1.083823	1.972615	0.922219
H	-1.555480	0.047146	2.522825
C	2.251599	-1.830108	-2.786073
H	1.922670	-2.337927	-3.693175
H	2.800990	-0.926215	-3.066486
H	2.970096	-2.473393	-2.266534
C	-2.651268	-1.918563	-2.583957
H	-2.994323	-2.893548	-2.215341
H	-3.476595	-1.216325	-2.449413
H	-2.441711	-2.028727	-3.649732
C	-2.757093	-0.146478	-0.343046
C	-3.693534	-0.811547	0.468663
C	-2.945657	1.196429	-0.738073
C	-4.812717	-0.095033	0.905086
C	-4.084029	1.863175	-0.285105
C	-5.008078	1.229227	0.539870
H	-5.542953	-0.593530	1.540152
H	-4.246926	2.897674	-0.577037
H	-5.883285	1.768518	0.891617
C	2.615527	-0.261169	-0.541633
C	3.580642	-1.092664	0.061573
C	2.896464	1.091146	-0.842950
C	4.835753	-0.550020	0.351922
C	4.169674	1.578256	-0.539495
C	5.134372	0.769989	0.051580
H	5.584823	-1.177618	0.830309
H	4.410395	2.613714	-0.760292
H	6.115590	1.175076	0.283339
C	1.835071	1.996544	-1.441626
H	0.921538	1.814707	-0.853437
C	3.289270	-2.526803	0.464910
H	2.341831	-2.833514	0.003016
C	-3.544424	-2.270114	0.856995
H	-2.651350	-2.667170	0.357211
C	-1.951680	1.870286	-1.664181
H	-0.977227	1.399779	-1.477480
C	-1.797837	3.366810	-1.419057
H	-2.708216	3.922458	-1.670577
H	-1.542510	3.593854	-0.378532
H	-0.999022	3.766693	-2.053234
C	-2.314190	1.621341	-3.130073
H	-3.317718	2.001313	-3.354919
H	-1.608362	2.129400	-3.796016
H	-2.295752	0.558011	-3.383947
C	-4.749832	-3.086198	0.388797
H	-5.663364	-2.782117	0.911891
H	-4.934078	-2.964466	-0.683427
H	-4.599320	-4.151697	0.589568
C	-3.336093	-2.440828	2.360664
H	-2.409233	-1.962682	2.692758
H	-4.167840	-2.006015	2.927185
H	-3.272534	-3.501388	2.624707
C	3.108214	-2.631718	1.980070
H	4.006666	-2.286933	2.505594
H	2.261759	-2.026446	2.324707
H	2.916558	-3.667432	2.279312
C	4.374342	-3.493623	-0.006461
H	4.562589	-3.412773	-1.082281
H	5.326506	-3.311164	0.503419
H	4.087283	-4.527372	0.208672
C	1.515076	1.672983	-2.903815
H	0.808596	2.410585	-3.302946
H	2.417933	1.718385	-3.524902
H	1.055871	0.689844	-3.033280
C	2.173947	3.479722	-1.334425
H	1.308683	4.083012	-1.625384
H	2.458052	3.774991	-0.320029
H	2.997487	3.756613	-2.003692
C	0.075910	1.678493	4.186369
H	-0.982777	1.650764	4.463570
H	0.650439	1.730569	5.117613
C	0.423499	2.856883	3.241739
H	1.206836	3.494379	3.673453
H	-0.437145	3.498212	3.026862
C	1.703833	0.971091	2.616147
H	2.116125	0.259161	1.891971
H	2.514801	1.303973	3.273516
C	0.490838	0.426739	3.373509
H	0.644762	-0.477069	3.970677
C	0.933792	2.132210	1.972602
H	1.509722	2.769732	1.294164

2a

SCF (wB97X) = -1513.96912071
H(0 K)= -1513.175182
H(298 K)= -1513.133564
G(298 K)= -1513.245450
Lowest Frequency = 26.0505cm-1
PCM (Benzene) Energy = -1513.97187861

Al	-0.038692	-0.454630	-0.083700
N	-1.497031	0.363485	-1.039228
C	-1.357729	1.170224	-2.088480
C	-0.102566	1.490110	-2.639556
H	-0.118847	2.105115	-3.532110
C	-2.551789	1.858667	-2.680670
H	-3.480904	1.312123	-2.510335
H	-2.413346	2.017219	-3.752361
H	-2.669272	2.847556	-2.222216
C	-2.767227	0.245091	-0.380468
C	-3.539433	-0.914284	-0.591946
C	-4.739891	-1.036463	0.110917
H	-5.355615	-1.919197	-0.037846
C	-5.154430	-0.056633	1.005697
H	-6.089544	-0.174919	1.546071
C	-4.364386	1.064390	1.218179
H	-4.679492	1.819731	1.935674
C	-3.156998	1.238508	0.537378

C	-3.091580	-1.984645	-1.570172
H	-1.994359	-2.013146	-1.539779
C	-3.590724	-3.376878	-1.201248
H	-3.373855	-3.623479	-0.157349
H	-3.107866	-4.129176	-1.831582
H	-4.671350	-3.479158	-1.353065
C	-3.501924	-1.640117	-3.002754
H	-4.588471	-1.515065	-3.079769
H	-3.209267	-2.439391	-3.691430
H	-3.032587	-0.717014	-3.354205
C	-2.304388	2.455986	0.843849
H	-1.426178	2.442795	0.184462
C	-1.793745	2.418138	2.285068
H	-1.171852	3.296142	2.495623
H	-2.627767	2.428666	2.996179
H	-1.199027	1.518250	2.471963
N	1.348274	0.539175	-0.954155
C	1.154404	1.266475	-2.069341
C	2.331769	1.939635	-2.713871
H	3.058904	1.210912	-3.085959
H	2.015360	2.567651	-3.547082
H	2.868309	2.560051	-1.988787
C	2.657500	0.521524	-0.361126
C	3.648521	-0.331691	-0.882644
C	4.893587	-0.356900	-0.249773
H	5.670572	-1.014180	-0.631775
C	5.148442	0.431130	0.863947
H	6.122923	0.396994	1.343222
C	4.151683	1.252875	1.375535
H	4.355824	1.850000	2.259253
C	2.887943	1.314386	0.783725
C	3.362910	-1.271477	-2.038740
H	2.534343	-0.855004	-2.624338
C	2.890801	-2.625296	-1.506676
H	1.959690	-2.527604	-0.937625
H	2.710349	-3.326691	-2.328173
H	3.643105	-3.067024	-0.842569
C	4.551122	-1.438716	-2.980634
H	5.375190	-1.981497	-2.505738
H	4.258789	-2.015943	-3.862763
H	4.945440	-0.475416	-3.321490
C	1.791268	2.188121	1.367483
H	0.860363	1.599473	1.329959
C	1.562151	3.461398	0.550363
H	0.790341	4.082277	1.020335
H	1.229871	3.250929	-0.470339
H	2.478303	4.061242	0.492966
C	2.022927	2.546433	2.830214
H	1.131102	3.027576	3.242813
H	2.853927	3.250752	2.953675
H	2.237402	1.662242	3.437671
C	-0.326144	-2.205125	0.711768
H	-1.270141	-2.732877	0.533183
C	-0.433795	-0.954761	1.743405
H	-1.440021	-0.795856	2.151774
C	0.590073	-1.328401	2.806958
H	0.940404	-0.484753	3.415784
C	0.025911	-2.493000	3.641172
H	0.640892	-2.678099	4.531528
H	-0.990907	-2.283436	3.990112
C	0.091765	-3.692720	2.659396
H	-0.892568	-4.126941	2.452349
H	0.719866	-4.498261	3.060487
C	0.717642	-3.082399	1.388963
H	1.183050	-3.830517	0.735954
C	1.677843	-2.059704	2.008252
H	2.184734	-1.429669	1.265880
H	2.443369	-2.517286	2.646778
C	-3.062289	3.758423	0.587632
H	-3.901137	3.871639	1.283407
H	-2.404574	4.622658	0.727899
H	-3.474519	3.807337	-0.425329

Ethylene
SCF (wB97X) = -78.5733279183
H(0 K)= -78.522392
H(298 K)= -78.519338
G(298 K)= -78.544576
Lowest Frequency = 816.8401cm-1
PCM (Benzene) Energy = -78.5738275916

C	-1.145001	1.804238	0.000000
H	-0.631519	0.846676	0.000000
H	-2.230927	1.767726	0.000000
C	-0.482203	2.955119	0.000000
H	-0.995687	3.912680	0.000000
H	0.603723	2.991629	0.000000

Int-1b
SCF (wB97X) = -1319.82210053
H(0 K)= -1319.131966
H(298 K)= -1319.091354
G(298 K)= -1319.203007
Lowest Frequency = 26.2665cm-1
PCM (Benzene) Energy = -1319.82511721

N	-1.557442	-0.434511	0.419212
Al	-0.099735	-0.461634	-0.994622
N	1.229239	-0.677232	0.523619
C	-1.486633	-0.851100	1.689012
C	-0.295763	-1.310587	2.268684
C	0.994768	-1.184703	1.739748
C	2.285175	1.974868	-2.819537
C	1.132694	2.625604	-2.946471
H	-0.368917	-1.706984	3.275244
H	1.089896	3.700907	-3.106171
H	3.246218	2.482337	-2.864432
C	-2.710154	-0.814382	2.561023
H	-2.468574	-1.071678	3.593223
H	-3.166517	0.180900	2.542344
H	-3.479830	-1.506585	2.203959
C	2.148531	-1.632609	2.591708

H	1.818830	-1.933790	3.587254
H	2.650784	-2.486123	2.121428
H	2.907144	-0.850733	2.690444
C	2.570085	-0.299166	0.186156
C	3.448233	-1.210875	-0.426037
C	2.950988	1.043628	0.413673
C	4.724835	-0.765909	-0.783288
C	4.242756	1.432674	0.056142
C	5.127487	0.538322	-0.536484
H	5.410329	-1.460677	-1.264718
H	4.554726	2.459989	0.224915
H	6.125748	0.864135	-0.815723
C	-2.806100	0.070620	-0.069664
C	-3.843182	-0.816900	-0.417703
C	-2.939561	1.462666	-0.269443
C	-5.037822	-0.282113	-0.905659
C	-4.156694	1.947498	-0.752220
C	-5.204396	1.087148	-1.057489
H	-5.846426	-0.955218	-1.181243
H	-4.282547	3.015821	-0.903343
H	-6.143020	1.484579	-1.433525
C	-1.786674	2.404558	0.027856
H	-0.872884	1.910067	-0.347291
C	-3.659656	-2.321415	-0.360705
H	-2.809694	-2.540778	0.296096
C	3.042836	-2.636261	-0.741882
H	2.071809	-2.824531	-0.265656
C	1.982653	2.038373	1.029379
H	0.976943	1.761212	0.680754
C	2.237652	3.478147	0.598135
H	3.161238	3.877177	1.032773
H	2.301420	3.569375	-0.489747
H	1.423091	4.122005	0.947604
C	1.966863	1.955131	2.558003
H	2.973663	2.107018	2.964794
H	1.318760	2.732250	2.977746
H	1.595174	0.993807	2.921611
C	4.040796	-3.654276	-0.193061
H	5.014636	-3.565638	-0.686928
H	4.210295	-3.528426	0.881445
H	3.684966	-4.675391	-0.362004
C	2.864143	-2.819861	-2.249094
H	2.114314	-2.128033	-2.648226
H	3.806424	-2.632293	-2.776874
H	2.546248	-3.840529	-2.486268
C	-3.302403	-2.850089	-1.750718
H	-4.114437	-2.653574	-2.459867
H	-2.400250	-2.365298	-2.141204
H	-3.128205	-3.931036	-1.729150
C	-4.876325	-3.051928	0.200320
H	-5.181076	-2.656036	1.175111
H	-5.740868	-2.974834	-0.467507
H	-4.661478	-4.117933	0.321817
C	-1.594612	2.633046	1.527177
H	-0.777358	3.341382	1.706726
H	-2.502939	3.053170	1.974758
H	-1.348532	1.709266	2.059538
C	-1.880782	3.737392	-0.702540
H	-0.945537	4.294040	-0.585457
H	-2.060016	3.603444	-1.773587
H	-2.683616	4.367282	-0.302707
H	2.313634	0.898010	-2.653914
H	0.181967	2.096386	-2.893918

TS-1b
SCF (wB97X) = -1319.80820837
H(0 K)= -1319.118052
H(298 K)= -1319.078988
G(298 K)= -1319.185892
Lowest Frequency = -273.4428cm-1
PCM (Benzene) Energy = -1319.81088367

N	-1.409068	-0.304633	0.508593
Al	0.009539	-1.017664	-0.633011
N	1.428607	-0.389058	0.548772
C	-1.262223	-0.438073	1.831629
C	-0.015890	-0.653779	2.448766
C	1.257266	-0.510034	1.866038
C	0.094689	-0.674820	-2.655337
C	0.149519	0.704994	-2.296349
H	-0.035446	-0.796528	3.523283
H	1.107790	1.214117	-2.258807
H	0.987156	-1.157182	-3.053955
C	-2.472079	-0.371273	2.716798
H	-2.192253	-0.290821	3.767745
H	-3.119530	0.469570	2.454103
H	-3.078512	-1.275975	2.590602
C	2.450577	-0.539485	2.775605
H	2.838442	-1.564948	2.822920
H	3.265941	0.088801	2.410592
H	2.190326	-0.236651	3.791247
C	2.683258	0.017079	-0.003215
C	3.634015	-0.940761	-0.395610
C	2.897776	1.397445	-0.209842
C	4.806825	-0.491256	-1.012010
C	4.089201	1.795131	-0.816241
C	5.037209	0.860429	-1.221735
H	5.550507	-1.221292	-1.326326
H	4.275658	2.853715	-0.979538
H	5.955509	1.189511	-1.700239
C	-2.695782	0.012353	-0.036057
C	-3.626534	-1.014777	-0.287498
C	-2.984478	1.357542	-0.355182
C	-4.858440	-0.667915	-0.850891
C	-4.233858	1.650200	-0.905157
C	-5.167812	0.649925	-1.150889
H	-5.583647	-1.452857	-1.056386
H	-4.477303	2.679138	-1.154312
H	-6.132619	0.900090	-1.583486
C	-1.964962	2.453157	-0.106301
H	-0.983619	2.010808	-0.319568
C	-3.337235	-2.475389	0.002961

H	-2.396261	-2.537359	0.565390
C	3.436773	-2.424701	-0.157516
H	2.510699	-2.556175	0.417506
C	1.871518	2.414310	0.250176
H	0.900829	1.901594	0.252528
C	1.755356	3.619202	-0.676140
H	2.665476	4.229484	-0.675769
H	1.547272	3.325356	-1.709711
H	0.940109	4.269672	-0.341858
C	2.153255	2.881376	1.679899
H	3.148286	3.335626	1.755817
H	1.420053	3.632188	1.993366
H	2.106444	2.059117	2.399158
C	4.586273	-3.015684	0.658237
H	5.529794	-2.979809	0.102408
H	4.741875	-2.473978	1.596795
H	4.391641	-4.065414	0.899651
C	3.268020	-3.182071	-1.473275
H	2.396035	-2.822915	-2.028987
H	4.150419	-3.062886	-2.112581
H	3.128841	-4.252774	-1.292206
C	-3.142428	-3.256117	-1.296954
H	-4.046838	-3.216268	-1.915210
H	-2.311620	-2.851421	-1.884006
H	-2.922245	-4.308630	-1.090874
C	-4.435023	-3.122284	0.846796
H	-4.631635	-2.565725	1.769024
H	-5.380827	-3.180775	0.297140
H	-4.157842	-4.144594	1.121729
C	-1.943126	2.921480	1.349999
H	-1.262623	3.773038	1.464066
H	-2.936771	3.247507	1.680050
H	-1.595505	2.139293	2.031438
C	-2.141895	3.648529	-1.036174
H	-1.295182	4.334147	-0.936823
H	-2.204847	3.344724	-2.085892
H	-3.044900	4.223367	-0.800489
H	-0.812928	-1.063994	-3.116753
H	-0.745528	1.315181	-2.362371

2b
SCF (wB97X) = -1319.83909748
H(0 K)= -1319.147065
H(298 K)= -1319.108327
G(298 K)= -1319.214087
Lowest Frequency = 23.3040cm-1
PCM (Benzene) Energy = -1319.84229917

N	1.413041	-0.349635	0.603587
Al	-0.003835	-0.437693	-0.681121
N	-1.429336	-0.129268	0.555014
C	1.226188	-0.280023	1.924775
C	-0.045198	-0.291162	2.521530
C	-1.288787	-0.162637	1.885864
C	0.156010	0.182907	-2.496768
C	-0.029318	-1.385737	-2.361325
H	-0.062105	-0.292204	3.605318
H	-0.951042	-1.757356	-2.814548
H	-0.649291	0.679236	-3.043801
C	2.408957	-0.110371	2.831646
H	2.173706	-0.439187	3.845268
H	3.288467	-0.647127	2.468568
H	2.687708	0.948816	2.884829
C	-2.505002	-0.035370	2.754631
H	-2.230406	0.136964	3.795833
H	-3.153927	0.777352	2.415725
H	-3.107635	-0.949279	2.707769
C	-2.712286	0.138575	-0.031383
C	-2.951082	1.433619	-0.541289
C	-3.663688	-0.890731	-0.154375
C	-4.180746	1.678554	-1.154784
C	-4.875806	-0.592663	-0.783486
C	-5.138441	0.677904	-1.274535
H	-4.388726	2.667486	-1.553276
H	-5.620437	-1.377960	-0.894591
H	-6.087190	0.889073	-1.760120
C	2.718921	-0.122981	0.051877
C	3.205331	1.194860	-0.046076
C	3.439824	-1.219192	-0.463300
C	4.455670	1.390070	-0.637749
C	4.686933	-0.969048	-1.039782
C	5.196578	0.321451	-1.122613
H	4.844582	2.402065	-0.729695
H	5.263967	-1.798089	-1.439408
H	6.167176	0.494057	-1.579356
C	2.883999	-2.628375	-0.372211
H	1.798217	-2.559164	-0.526170
C	2.396424	2.400132	0.397754
H	1.532573	2.049299	0.977720
C	-1.910398	2.530969	-0.406548
H	-0.925632	2.060108	-0.538151
C	-3.402461	-2.304785	0.328620
H	-2.504344	-2.292673	0.958024
C	-3.113875	-3.234188	-0.850392
H	-3.963618	-3.259448	-1.542175
H	-2.231764	-2.905303	-1.407180
H	-2.935955	-4.257528	-0.503600
C	-4.556052	-2.848619	1.169753
H	-5.460195	-2.989644	0.567875
H	-4.297303	-3.824043	1.592993
H	-4.819003	-2.179430	1.996036
C	-1.933368	3.165227	0.985095
H	-2.916793	3.597308	1.205002
H	-1.699135	2.442517	1.772458
H	-1.193946	3.971144	1.053119
C	-2.025920	3.604659	-1.480788
H	-2.048248	3.170593	-2.484616
H	-2.925304	4.218638	-1.356499
H	-1.168098	4.281775	-1.428001
C	1.850516	3.150106	-0.819204
H	2.670369	3.529208	-1.440161
H	1.229130	2.496862	-1.441570

H	1.246820	4.009553	-0.505611
C	3.200605	3.342925	1.290716
H	3.638638	2.826755	2.151582
H	4.023997	3.811461	0.740825
H	2.565029	4.150155	1.668250
C	3.112591	-3.231614	1.014410
H	2.741964	-4.261060	1.053951
H	4.180738	-3.252493	1.261210
H	2.597011	-2.668220	1.797163
C	3.429696	-3.559124	-1.448093
H	2.878972	-4.504082	-1.441867
H	3.338740	-3.123547	-2.447487
H	4.485403	-3.803396	-1.282915
H	1.120607	0.476394	-2.919943
H	0.815301	-1.975280	-2.726247

Propylene
SCF (wB97X) = -117.889239091
H(0 K)= -117.809499
H(298 K)= -117.805402
G(298 K)= -117.834502
Lowest Frequency = 208.7048cm-1
PCM (Benzene) Energy = -117.889747995

C	6.051980	15.756385	6.514615
C	6.473952	14.463512	5.907870
H	7.137302	14.531403	5.044395
C	6.104459	13.258865	6.338803
H	5.444444	13.141474	7.195794
H	6.443748	12.347648	5.856486
H	5.523803	16.386668	5.789774
H	5.390678	15.602178	7.371727
H	6.914723	16.340910	6.854646

Int-1c
SCF (wB97X) = -1359.13986146
H(0 K)= -1358.421315
H(298 K)= -1358.379228
G(298 K)= -1358.495093
Lowest Frequency = 21.8347cm-1
PCM (Benzene) Energy = -1359.14311938

N	1.754848	-0.316178	-0.507828
Al	0.161942	-0.294880	0.750060
N	-1.011027	-0.568865	-0.883350
C	1.814830	-0.689353	-1.791394
C	0.676881	-1.032659	-2.532900
C	-0.656885	-0.911378	-2.128268
C	-3.041482	0.738635	3.061944
C	-3.644792	1.920662	3.184182
H	0.843993	-1.337675	-3.559798
H	-4.105585	2.412748	2.331777
C	3.139109	-0.726332	-2.501383
H	3.011776	-0.916325	-3.568121
H	3.681041	0.215683	-2.370288
H	3.786149	-1.507828	-2.088349
C	-1.722228	-1.151000	-3.159068
H	-2.416664	-1.931468	-2.829136
H	-2.330480	-0.252767	-3.308381
H	-1.293010	-1.445550	-4.117678
C	-2.391492	-0.267560	-0.636903
C	-3.286378	-1.282134	-0.245903
C	-2.808366	1.076144	-0.720816
C	-4.604211	-0.927639	0.051855
C	-4.136682	1.380765	-0.413486
C	-5.032463	0.391422	-0.030356
H	-5.301661	-1.699909	0.368819
H	-4.466733	2.416994	-0.466761
H	-6.058795	0.648885	0.215683
C	2.962515	0.115049	0.133023
C	3.894172	-0.829321	0.604757
C	3.151560	1.498380	0.343288
C	5.045124	-0.362515	1.243891
C	4.322577	1.913858	0.980451
C	5.268639	0.995742	1.419894
H	5.772257	-1.079852	1.617772
H	4.491782	2.973826	1.146783
H	6.171767	1.340600	1.915714
C	2.112240	2.506555	-0.114718
H	1.122752	2.057090	0.078256
C	3.638740	-2.320979	0.507241
H	2.854088	-2.486453	-0.240792
C	-2.832205	-2.716223	-0.067641
H	-1.858122	-2.825466	-0.560970
C	-1.858687	2.194931	-1.105732
H	-0.894458	1.745649	-1.378438
C	-1.614722	3.128033	0.079764
H	-2.545527	3.607776	0.404031
H	-1.213062	2.583941	0.943865
H	-0.907015	3.920966	-0.188452
C	-2.360274	2.976643	-2.318110
H	-3.306069	3.486412	-2.103346
H	-1.636312	3.744187	-2.610523
H	-2.527345	2.326402	-3.182613
C	-3.789219	-3.723618	-0.698547
H	-4.761973	-3.729222	-0.195213
H	-3.970258	-3.509953	-1.757142
H	-3.384431	-4.737565	-0.623966
C	-2.632617	-3.011502	1.419919
H	-1.908175	-2.320248	1.868595
H	-3.576777	-2.896709	1.965883
H	-2.273384	-4.033853	1.577980
C	3.108418	-2.844985	1.842480
H	3.849124	-2.699312	2.636835
H	2.196215	-2.316833	2.143257
H	2.882061	-3.914919	1.785245
C	4.869735	-3.108663	0.067582
H	5.294701	-2.721740	-0.864872
H	5.661914	-3.079223	0.823231
H	4.616524	-4.161702	-0.088618
C	2.195279	2.776991	-1.617256
H	1.452563	3.525390	-1.916460

H	3.184503	3.163905	-1.888729
H	2.010259	1.876265	-2.209749
C	2.158356	3.814326	0.663981
H	1.306071	4.444877	0.394058
H	2.122541	3.644555	1.744063
H	3.063240	4.390873	0.441686
H	-3.699293	2.437846	4.140516
H	-3.010486	0.266348	2.076995
C	-2.383651	-0.011022	4.166242
H	-1.331467	-0.208198	3.926960
H	-2.429117	0.534511	5.113434
H	-2.852031	-0.991746	4.316352

TS-1c endo
SCF (wB97X) = -1359.12103360
H(0 K)= -1358.402020
H(298 K)= -1358.361800
G(298 K)= -1358.470469
Lowest Frequency = -263.8601cm-1
PCM (Benzene) Energy = -1359.12360606

N	-1.354924	-0.196601	0.672313
Al	0.016327	-0.996914	-0.478072
N	1.475808	-0.351641	0.652754
C	-1.184738	-0.258718	1.995701
C	0.070757	-0.470900	2.597553
C	1.331205	-0.399841	1.978970
C	0.063153	-0.730498	-2.544022
C	0.160264	0.648443	-2.194383
H	0.072013	-0.553300	3.678725
H	1.128772	1.139793	-2.177072
C	-2.370901	-0.127007	2.905903
H	-2.069659	0.179409	3.908790
H	-3.107911	0.581052	2.519967
H	-2.879014	-1.095366	2.990622
C	2.546561	-0.420776	2.859859
H	2.316983	-0.083477	3.871999
H	2.921360	-1.449876	2.927301
H	3.361700	0.183684	2.455861
C	2.720584	0.019276	0.057456
C	3.649264	-0.961931	-0.329860
C	2.945935	1.390001	-0.199085
C	4.810755	-0.546895	-0.990103
C	4.125918	1.753513	-0.847906
C	5.052078	0.794710	-1.247601
H	5.537269	-1.295802	-1.300275
H	4.320257	2.804105	-1.049765
H	5.961612	1.097057	-1.759322
C	-2.641514	0.083519	0.109671
C	-3.576278	-0.957025	-0.059708
C	-2.912954	1.394454	-0.342049
C	-4.781377	-0.664111	-0.706391
C	-4.135089	1.633619	-0.972584
C	-5.062825	0.614852	-1.161396
H	-5.507205	-1.461774	-0.852764
H	-4.362964	2.633892	-1.329543
H	-6.004331	0.821881	-1.662652
C	-1.910520	2.513140	-0.130334
H	-0.917718	2.068286	-0.271313
C	-3.330870	-2.375528	0.419387
H	-2.395064	-2.391753	0.993066
C	3.438869	-2.434855	-0.040454
H	2.528330	-2.534360	0.564953
C	1.942152	2.434339	0.250069
H	0.967811	1.931340	0.306229
C	1.804387	3.600126	-0.722508
H	2.718785	4.201335	-0.778545
H	1.558878	3.265009	-1.735145
H	1.005832	4.271494	-0.388970
C	2.273116	2.960222	1.648407
H	3.269476	3.417384	1.669737
H	1.550413	3.723570	1.955965
H	2.253917	2.168986	2.402649
C	4.603595	-3.017262	0.759577
H	5.531163	-3.011383	0.176389
H	4.793236	-2.449899	1.676464
H	4.402151	-4.056409	1.038282
C	3.220506	-3.229886	-1.326501
H	2.333313	-2.879918	-1.864242
H	4.082560	-3.138864	-1.997547
H	3.077546	-4.293152	-1.108255
C	-3.155617	-3.332167	-0.758921
H	-4.042567	-3.328415	-1.403526
H	-2.290585	-3.050617	-1.368251
H	-2.999410	-4.357633	-0.408352
C	-4.456138	-2.860633	1.333796
H	-4.638691	-2.172658	2.165765
H	-5.399326	-2.961345	0.785636
H	-4.218239	-3.843926	1.751422
C	-1.956612	3.072211	1.292937
H	-1.274982	3.925057	1.388265
H	-2.962844	3.422442	1.551980
H	-1.651760	2.330893	2.037301
C	-2.057013	3.645183	-1.140659
H	-1.222811	4.346715	-1.047149
H	-2.069366	3.276295	-2.171359
H	-2.975764	4.221382	-0.981584
H	-0.725297	1.273611	-2.273775
H	0.991242	-1.208259	-2.868454
C	-1.158252	-1.236438	-3.271734
H	-1.216974	-2.328400	-3.276279
H	-2.085123	-0.852080	-2.828966
H	-1.135854	-0.887240	-4.313322

TS-1c exo
SCF (wB97X) = -1359.11860442
H(0 K)= -1358.399498
H(298 K)= -1358.359457
G(298 K)= -1358.467324
Lowest Frequency = -299.5091cm-1
PCM (Benzene) Energy = -1359.12116027

N	-1.373420	-0.420269	0.577372
Al	0.067537	-1.032222	-0.588674
N	1.465391	-0.383744	0.610431
C	-1.204071	-0.662055	1.887313
C	0.052420	-0.866309	2.482142
C	1.315083	-0.594406	1.915038
C	0.481343	-0.740290	-2.561960
C	0.160167	0.636272	-2.315035
H	0.045434	-1.085792	3.543917
H	0.999399	1.313512	-2.164030
H	1.536989	-1.004812	-2.656217
C	-2.406472	-0.692224	2.784245
H	-2.120129	-0.848288	3.824681
H	-2.978044	0.238150	2.711734
H	-3.091214	-1.492779	2.482955
C	2.503793	-0.588446	2.829766
H	2.881832	-1.613337	2.933289
H	3.323102	0.018604	2.438951
H	2.238956	-0.237069	3.828963
C	2.701646	0.055431	0.043939
C	3.675569	-0.881109	-0.347063
C	2.863571	1.435408	-0.205189
C	4.810899	-0.409185	-1.014271
C	4.017671	1.855879	-0.865740
C	4.983030	0.942107	-1.277371
H	5.570782	-1.121840	-1.329876
H	4.161056	2.914810	-1.065491
H	5.871127	1.287149	-1.799585
C	-2.682266	-0.066614	0.110851
C	-3.601358	-1.078599	-0.226285
C	-3.011340	1.299990	-0.033513
C	-4.866092	-0.701423	-0.688091
C	-4.294366	1.621358	-0.480843
C	-5.219090	0.633981	-0.802320
H	-5.581094	-1.474967	-0.960695
H	-4.572720	2.664419	-0.596638
H	-6.209167	0.910040	-1.154592
C	-1.984485	2.385986	0.235072
H	-1.049302	2.029715	-0.224952
C	-3.253879	-2.553035	-0.151221
H	-2.301705	-2.658365	0.385393
C	3.545691	-2.363818	-0.056314
H	2.626178	-2.520362	0.522385
C	1.827495	2.426113	0.285297
H	0.866203	1.895848	0.296094
C	1.671857	3.647965	-0.611564
H	2.562536	4.286308	-0.598384
H	1.471407	3.373160	-1.652121
H	0.836780	4.264117	-0.261399
C	2.133354	2.862562	1.719739
H	3.123804	3.328185	1.784432
H	1.397642	3.594306	2.070233
H	2.115189	2.019435	2.416552
C	4.723063	-2.864469	0.781065
H	5.663321	-2.801854	0.221985
H	4.851151	-2.279649	1.697533
H	4.581590	-3.912670	1.063086
C	3.418129	-3.182329	-1.339734
H	2.523332	-2.903540	-1.904552
H	4.289245	-3.037573	-1.989112
H	3.346929	-4.250899	-1.112569
C	-3.050280	-3.119862	-1.556711
H	-3.971764	-3.041918	-2.145504
H	-2.262710	-2.580471	-2.094281
H	-2.762166	-4.175334	-1.516557
C	-4.306940	-3.363923	0.601397
H	-4.498148	-2.964603	1.602931
H	-5.263966	-3.375573	0.068503
H	-3.985603	-4.404249	0.710139
C	-1.705521	2.618446	1.722629
H	-1.046973	3.485718	1.847085
H	-2.632107	2.833557	2.268401
H	-1.209885	1.771292	2.202941
C	-2.346774	3.720954	-0.408405
H	-1.500941	4.411963	-0.342234
H	-2.613914	3.618162	-1.464281
H	-3.190007	4.200242	0.102972
H	-0.143447	-1.326964	-3.236113
C	-1.117023	1.195806	-2.854528
H	-1.331892	2.197615	-2.469771
H	-1.987007	0.568237	-2.606557
H	-1.110809	1.274356	-3.955550

2c

SCF (wB97X) = -1359.14824567
H(0 K)= -1358.427805
H(298 K)= -1358.387540
G(298 K)= -1358.496699
Lowest Frequency = 28.8836cm-1
PCM (Benzene) Energy = -1359.15122015

N	-1.361560	-0.145478	0.689826
Al	0.027689	-0.307824	-0.623833
N	1.464941	-0.349501	0.639217
C	-1.184689	-0.306303	2.006570
C	0.072305	-0.529143	2.591498
C	1.323954	-0.485773	1.961491
C	-0.056426	-0.955853	-2.461448
C	0.169889	0.604698	-2.314248
H	0.080886	-0.661468	3.667179
H	1.121231	0.954937	-2.724963
C	-2.369366	-0.227152	2.925540
H	-2.052778	-0.075521	3.958227
H	-3.043821	0.582881	2.634605
H	-2.959680	-1.149377	2.887090
C	2.550744	-0.576184	2.817798
H	3.093478	-1.504681	2.605552
H	3.248526	0.238023	2.600687
H	2.301238	-0.557572	3.879047
C	2.736900	0.013342	0.081026
C	3.656203	-0.975147	-0.308239
C	2.986416	1.385278	-0.137104

C	4.837375	-0.565425	-0.933562
C	4.186400	1.741874	-0.754028
C	5.104162	0.778407	-1.156581
H	5.556651	-1.318483	-1.249334
H	4.402220	2.794853	-0.922301
H	6.028541	1.077019	-1.643170
C	-2.674019	0.088012	0.157833
C	-3.573846	-0.988012	0.029846
C	-2.990271	1.382413	-0.310473
C	-4.805572	-0.742531	-0.582775
C	-4.237337	1.571168	-0.909779
C	-5.137748	0.521403	-1.048715
H	-5.509259	-1.562777	-0.704207
H	-4.505737	2.555480	-1.281643
H	-6.099150	0.690416	-1.525764
C	-2.013177	2.534494	-0.149708
H	-1.013022	2.144415	-0.389330
C	-3.230452	-2.396409	0.480783
H	-2.371258	-2.344348	1.158879
C	3.398776	-2.451092	-0.079067
H	2.503387	-2.543082	0.548507
C	2.023845	2.465065	0.326214
H	1.075302	1.985419	0.600076
C	1.707500	3.483069	-0.764818
H	2.602111	4.022821	-1.094549
H	1.255583	2.996800	-1.634147
H	1.001794	4.231777	-0.387788
C	2.559341	3.161498	1.578541
H	3.512123	3.663054	1.373155
H	1.853434	3.920194	1.933116
H	2.728760	2.456576	2.399132
C	4.559766	-3.119247	0.656268
H	5.467259	-3.131324	0.042823
H	4.807616	-2.603426	1.590029
H	4.318767	-4.159362	0.896930
C	3.112154	-3.172267	-1.395171
H	2.232240	-2.757010	-1.894756
H	3.962001	-3.083464	-2.081558
H	2.932835	-4.238776	-1.224345
C	-2.796815	-3.257287	-0.704629
H	-3.578752	-3.293216	-1.471951
H	-1.890876	-2.856447	-1.169478
H	-2.591673	-4.284045	-0.383595
C	-4.377655	-3.054877	1.243957
H	-4.735706	-2.429686	2.069140
H	-5.235067	-3.256904	0.593450
H	-4.059335	-4.015751	1.659638
C	-1.971631	3.051784	1.289281
H	-1.285286	3.902326	1.369780
H	-2.961141	3.392344	1.616648
H	-1.625590	2.289715	1.993807
C	-2.273032	3.684113	-1.114488
H	-1.458868	4.412570	-1.057269
H	-2.338527	3.337970	-2.150297
H	-3.199355	4.220431	-0.877796
H	-0.651630	1.187987	-2.743448
H	0.836206	-1.441323	-2.870368
C	-1.287607	-1.335433	-3.251867
H	-1.442751	-2.419833	-3.299123
H	-2.203148	-0.905058	-2.820633
H	-1.229924	-0.969397	-4.288825

Int-2

SCF (wB97X) = -1359.14073026
H(0 K)= -1358.421834
H(298 K)= -1358.380098
G(298 K)= -1358.494030
Lowest Frequency = 20.6249cm-1
PCM (Benzene) Energy = -1359.14355501

Al	-0.153620	-0.415901	-0.930632
N	-1.661423	-0.450586	0.436834
C	-1.638526	-0.942587	1.680349
C	-0.470607	-1.439960	2.275457
H	-0.581561	-1.889411	3.255893
C	0.839126	-1.294114	1.801747
N	1.123534	-0.721010	0.625614
C	-2.893345	-0.954448	2.507611
H	-3.354394	0.038652	2.522380
H	-2.688670	-1.263743	3.533550
H	-3.645042	-1.630892	2.087502
C	1.955240	-1.786608	2.679250
H	1.579688	-2.148569	3.637550
H	2.698016	-1.004440	2.862906
H	2.491607	-2.605923	2.187105
C	-2.891561	0.082470	-0.068088
C	-3.021277	1.484189	-0.188523
C	-4.218909	1.996229	-0.691478
H	-4.342125	3.071417	-0.783870
C	-5.250514	1.155282	-1.091401
H	-6.173594	1.574299	-1.482251
C	-5.086896	-0.220320	-1.013694
H	-5.881659	-0.876378	-1.361496
C	-3.912053	-0.782839	-0.508887
C	-1.883976	2.406783	0.212217
H	-0.956199	1.946580	-0.171908
C	-1.968009	3.790758	-0.416803
H	-2.094086	3.740131	-1.502482
H	-1.049854	4.349821	-0.209838
H	-2.799166	4.375989	-0.007066
C	-1.736876	2.527768	1.729165
H	-2.662192	2.905759	2.179620
H	-0.934542	3.230667	1.981641
H	-1.493721	1.571611	2.201441
C	-3.730819	-2.288632	-0.530284
H	-2.899588	-2.545592	0.136864
C	-4.963630	-3.045697	-0.044074
H	-5.295564	-2.703503	0.942078
H	-4.753476	-4.117392	0.024370
H	-5.809109	-2.929644	-0.730451
C	-3.336717	-2.741807	-1.936814
H	-4.129060	-2.504544	-2.655772

H	-3.165306	-3.822959	-1.969382
H	-2.423310	-2.239380	-2.275189
C	2.486619	-0.375461	0.347915
C	3.366730	-1.310395	-0.227864
C	4.673869	-0.904993	-0.515657
H	5.360800	-1.617989	-0.967158
C	5.100721	0.387754	-0.249061
H	6.119839	0.685620	-0.479822
C	4.214555	1.306097	0.303582
H	4.549806	2.322247	0.493352
C	2.898054	0.952916	0.605952
C	2.926938	-2.713275	-0.595757
H	1.943625	-2.890450	-0.141578
C	3.885206	-3.780855	-0.072034
H	4.866229	-3.711720	-0.554458
H	3.496064	-4.782919	-0.277045
H	4.048356	-3.696040	1.007568
C	2.763934	-2.834186	-2.111598
H	2.035291	-2.109117	-2.491596
H	2.426438	-3.837215	-2.392974
H	3.717236	-2.645835	-2.619787
C	1.940581	1.965710	1.210753
H	0.955623	1.786893	0.751963
C	2.323728	3.414049	0.930001
H	3.210331	3.716449	1.499246
H	1.509621	4.080474	1.234403
H	2.524218	3.588352	-0.129590
C	1.785149	1.777663	2.723133
H	1.307065	0.831556	2.986158
H	1.169751	2.578489	3.146989
H	2.762952	1.816214	3.217874
C	1.103430	2.829647	-2.448807
H	0.234333	2.313309	-2.851908
H	0.924952	3.781355	-1.954023
C	2.329434	2.315192	-2.564545
C	2.661514	1.021614	-3.218947
H	3.174196	2.863869	-2.141088
H	3.384552	1.158509	-4.031981
H	1.769491	0.533649	-3.621215
H	3.127745	0.331966	-2.502342

TS-2
SCF (wB97X) = -1359.11262469
H(0 K)= -1358.397459
H(298 K)= -1358.357470
G(298 K)= -1358.465680
Lowest Frequency = -1066.6195cm-1
PCM (Benzene) Energy = -1359.11540831

Al	-0.060115	-0.595517	-0.749265
N	-1.515512	-0.292666	0.574089
C	-1.407237	-0.493886	1.893020
C	-0.175742	-0.672702	2.535733
H	-0.212349	-0.856851	3.603464
C	1.101858	-0.524452	1.975457
N	1.305516	-0.323974	0.670126
C	-2.644474	-0.526394	2.743133
H	-3.276441	0.347938	2.559645
H	-2.396490	-0.568259	3.804478
H	-3.257805	-1.400474	2.497722
C	2.271536	-0.623020	2.912730
H	1.976167	-0.401954	3.939738
H	3.091016	0.039729	2.624761
H	2.669717	-1.644349	2.895154
C	-2.809574	-0.016090	0.022758
C	-3.134521	1.324529	-0.278228
C	-4.386888	1.593143	-0.833052
H	-4.657269	2.619202	-1.066651
C	-5.287602	0.569907	-1.105993
H	-6.256121	0.799073	-1.541812
C	-4.937947	-0.745164	-0.836423
H	-5.633981	-1.547012	-1.074032
C	-3.700810	-1.066118	-0.270201
C	-2.142438	2.440563	-0.009466
H	-1.147018	2.028223	-0.214765
C	-2.332172	3.642842	-0.926524
H	-2.386563	3.346940	-1.978554
H	-1.494199	4.338111	-0.816458
H	-3.243424	4.202482	-0.686957
C	-2.136816	2.882058	1.454974
H	-3.137280	3.190043	1.780696
H	-1.465232	3.737156	1.591889
H	-1.788684	2.087599	2.122308
C	-3.344004	-2.522064	-0.040198
H	-2.433608	-2.559239	0.571322
C	-4.440605	-3.281276	0.704561
H	-4.720001	-2.792125	1.643672
H	-4.113156	-4.298804	0.938884
H	-5.350819	-3.367404	0.101309
C	-3.030912	-3.209537	-1.369820
H	-3.903788	-3.187825	-2.032489
H	-2.754296	-4.257452	-1.213642
H	-2.202115	-2.715787	-1.888769
C	2.612972	0.023336	0.196721
C	3.551002	-0.967079	-0.146126
C	4.763678	-0.556238	-0.709873
H	5.495229	-1.311704	-0.990879
C	5.053463	0.786205	-0.903869
H	6.003359	1.082149	-1.340360
C	4.125601	1.753915	-0.531641
H	4.358111	2.805183	-0.680947
C	2.891558	1.396271	0.010877
C	3.321445	-2.444579	0.108205
H	2.341563	-2.558641	0.589734
C	4.390185	-3.003661	1.050785
H	5.374633	-3.001802	0.569551
H	4.164653	-4.039261	1.324802
H	4.481753	-2.419011	1.971383
C	3.299902	-3.258277	-1.184438
H	2.491025	-2.940716	-1.845902
H	3.165368	-4.322802	-0.966392
H	4.245073	-3.151900	-1.729801

C	1.878342	2.454535	0.406163
H	0.881589	2.005805	0.296601
C	1.925771	3.693917	-0.479999
H	2.833168	4.284532	-0.311243
H	1.076657	4.348729	-0.257034
H	1.887203	3.438018	-1.543729
C	2.028021	2.848621	1.877044
H	1.836158	2.007291	2.549227
H	1.323169	3.644707	2.139417
H	3.039793	3.217860	2.081250
C	0.109202	0.949101	-2.298073
H	-0.821437	0.784619	-2.851779
H	0.140768	1.973038	-1.917670
C	1.303837	0.463333	-2.917992
C	1.318009	-0.875871	-3.348909
H	2.251336	0.901389	-2.602701
H	2.268279	-1.313454	-3.658145
H	0.481090	-1.228341	-3.955584
H	0.818830	-1.501657	-2.207780

4c

SCF (wB97X) = -1359.18170408
H(0 K)= -1358.465845
H(298 K)= -1358.424868
G(298 K)= -1358.536601
Lowest Frequency = 24.7114cm-1
PCM (Benzene) Energy = -1359.18492584

Al	-0.031036	-0.752990	-0.546963
N	-1.504101	-0.238046	0.614107
C	-1.406370	-0.355402	1.943630
C	-0.184018	-0.531153	2.614334
H	-0.243822	-0.651733	3.689855
C	1.107036	-0.426764	2.073082
N	1.328604	-0.311362	0.761213
C	-2.649593	-0.285251	2.780829
H	-3.261352	0.581111	2.511745
H	-2.411519	-0.236605	3.843815
H	-3.277645	-1.166077	2.607394
C	2.272421	-0.455401	3.017213
H	1.954977	-0.330974	4.053246
H	3.011437	0.312653	2.773862
H	2.791236	-1.417819	2.931266
C	-2.783914	0.025895	0.022112
C	-3.076144	1.344879	-0.386520
C	-4.321630	1.597717	-0.963361
H	-4.567403	2.607847	-1.278882
C	-5.246986	0.577751	-1.154317
H	-6.210985	0.794861	-1.605981
C	-4.924878	-0.720626	-0.788178
H	-5.635903	-1.524385	-0.968450
C	-3.693768	-1.025481	-0.199834
C	-2.056917	2.453650	-0.207982
H	-1.071594	2.000419	-0.371534
C	-2.212324	3.576897	-1.226011
H	-2.260780	3.192104	-2.249286
H	-1.362034	4.263091	-1.165202
H	-3.114755	4.172240	-1.047211
C	-2.051284	3.015842	1.214381
H	-3.044291	3.383592	1.498054
H	-1.352166	3.856334	1.291292
H	-1.741151	2.267465	1.950531
C	-3.360444	-2.471647	0.111632
H	-2.423367	-2.496701	0.681543
C	-4.443424	-3.154761	0.945148
H	-4.665069	-2.610207	1.868937
H	-4.136496	-4.168910	1.218350
H	-5.382633	-3.241042	0.387845
C	-3.127762	-3.247656	-1.185892
H	-4.041135	-3.271882	-1.791731
H	-2.836343	-4.281490	-0.974903
H	-2.334393	-2.796548	-1.789031
C	2.622486	0.055724	0.269847
C	3.575487	-0.929022	-0.043023
C	4.793544	-0.516469	-0.591247
H	5.538859	-1.267976	-0.844497
C	5.061621	0.825191	-0.823421
H	6.014858	1.125356	-1.249702
C	4.101989	1.784976	-0.519317
H	4.311884	2.833762	-0.714292
C	2.866838	1.423232	0.020667
C	3.307114	-2.406527	0.158030
H	2.343931	-2.510312	0.673624
C	4.381819	-3.066672	1.020371
H	5.356415	-3.055282	0.519717
H	4.131390	-4.113760	1.216985
H	4.507260	-2.561535	1.983932
C	3.186905	-3.119801	-1.188680
H	2.409920	-2.672246	-1.816306
H	2.942339	-4.177493	-1.047455
H	4.132370	-3.066002	-1.741698
C	1.820988	2.472719	0.345636
H	0.843042	1.974250	0.313084
C	1.789822	3.616871	-0.661416
H	2.688311	4.241054	-0.603349
H	0.936026	4.272824	-0.460266
H	1.702934	3.252804	-1.690474
C	1.996004	3.016084	1.765008
H	1.862625	2.236272	2.520762
H	1.263266	3.802788	1.973859
H	2.995301	3.446405	1.898775
C	0.149582	0.355209	-2.199340
H	-0.773742	0.229057	-2.783486
H	0.232721	1.423466	-1.962993
C	1.328440	-0.104027	-2.978147
C	1.332677	-1.022811	-3.951581
H	2.290484	0.318812	-2.672769
H	2.252867	-1.339435	-4.432535
H	0.412046	-1.489815	-4.297687
H	-0.077866	-2.324625	-0.767378

Butene
SCF (wB97X) = -157.197786537
H(0 K)= -157.089242
H(298 K)= -157.083945
G(298 K)= -157.116780
Lowest Frequency = 103.7357cm-1
PCM (Benzene) Energy = -157.198274057

C	-1.137357	1.935701	-0.281011
H	-0.670374	1.195594	-0.927772
H	-2.218226	1.890457	-0.190807
C	-0.411905	2.844807	0.367031
H	-0.921201	3.573834	1.001593
C	1.073896	2.979737	0.289889
H	1.506900	2.804586	1.285025
H	1.479576	2.195357	-0.360659
C	1.513648	4.353799	-0.202954
H	1.127639	5.148696	0.443638
H	2.603181	4.444271	-0.221704
H	1.142291	4.547330	-1.213534

Int-1d'
SCF (wB97X) = -1398.45039134
H(0 K)= -1397.703084
H(298 K)= -1397.659825
G(298 K)= -1397.777614
Lowest Frequency = 29.6346cm-1
PCM (Benzene) Energy = -1398.45334490

N	1.037186	-1.069372	-0.584562
Al	-0.163272	-0.337508	0.876706
N	-1.718348	-0.626341	-0.391650
C	0.696480	-1.839158	-1.624999
C	-0.632226	-2.015462	-2.027926
C	-1.758683	-1.361343	-1.509898
C	1.268651	3.197725	2.169247
C	1.263469	4.503273	1.899304
H	-0.795362	-2.646652	-2.894310
H	0.352695	5.094695	1.924462
C	1.768402	-2.545649	-2.405705
H	1.355777	-3.076435	-3.265041
H	2.540580	-1.853401	-2.753905
H	2.280674	-3.271827	-1.763428
C	-3.043988	-1.489755	-2.278177
H	-3.801963	-2.034404	-1.705495
H	-3.471134	-0.502642	-2.483479
H	-2.888913	-2.010491	-3.224121
C	-2.913266	0.045792	0.028150
C	-3.943318	-0.674151	0.664626
C	-2.996706	1.445651	-0.140467
C	-5.085188	0.020602	1.070053
C	-4.162962	2.091587	0.275034
C	-5.205946	1.387664	0.864545
H	-5.887755	-0.521163	1.565214
H	-4.251217	3.166398	0.145790
H	-6.104100	1.910153	1.181871
C	2.408840	-0.678097	-0.448696
C	3.306223	-1.431507	0.328297
C	2.805033	0.536414	-1.052960
C	4.616158	-0.964361	0.468113
C	4.130811	0.947175	-0.904081
C	5.033436	0.206215	-0.150147
H	5.317548	-1.533710	1.075131
H	4.454369	1.876801	-1.365782
H	6.058102	0.548906	-0.035633
C	1.816833	1.385225	-1.833177
H	0.814297	1.151673	-1.448272
C	2.883990	-2.701099	1.038441
H	1.879090	-2.965142	0.684030
C	-3.801481	-2.148021	0.993834
H	-3.010941	-2.568389	0.361089
C	-1.847769	2.228350	-0.751541
H	-0.918881	1.816506	-0.316250
C	-1.863917	3.709290	-0.398033
H	-2.681275	4.239751	-0.900002
H	-1.970272	3.867698	0.679362
H	-0.928079	4.180758	-0.713046
C	-1.761494	2.044034	-2.266839
H	-2.690792	2.367966	-2.749998
H	-0.944693	2.646085	-2.681800
H	-1.576575	1.003139	-2.549546
C	-5.075176	-2.945909	0.729921
H	-5.883455	-2.659689	1.411170
H	-5.444720	-2.805475	-0.291610
H	-4.895992	-4.014936	0.879403
C	-3.350197	-2.310924	2.446052
H	-2.409297	-1.780384	2.632661
H	-4.099706	-1.901107	3.132423
H	-3.203963	-3.366528	2.698182
C	2.797134	-2.466417	2.546619
H	3.776453	-2.187969	2.953513
H	2.097416	-1.657211	2.783140
H	2.463953	-3.370277	3.067253
C	3.814330	-3.871812	0.727800
H	3.911500	-4.045611	-0.349049
H	4.822059	-3.697436	1.120492
H	3.443922	-4.793601	1.187060
C	1.813756	1.029982	-3.321103
H	1.128479	1.682215	-3.873375
H	2.813463	1.152750	-3.754105
H	1.494809	-0.001692	-3.497180
C	2.037515	2.882342	-1.643124
H	1.227267	3.444009	-2.121450
H	2.057486	3.162974	-0.584911
H	2.971144	3.223140	-2.104195
H	2.180589	5.031535	1.642930
H	0.329759	2.698303	2.425422
C	2.472637	2.315424	2.151656
H	2.347077	1.563914	1.356843
H	3.362384	2.899484	1.879952
C	2.685653	1.575983	3.465840
H	3.553494	0.911462	3.409274

H	2.841514	2.270070	4.297649
H	1.811669	0.959010	3.704040

TS-1d’
SCF (wB97X) = -1398.43090507
H(0 K)= -1397.683620
H(298 K)= -1397.641898
G(298 K)= -1397.754313
Lowest Frequency = -257.3555cm-1
PCM (Benzene) Energy = -1398.43342140

N	1.303121	-0.088927	-0.863697
Al	-0.033440	-1.004500	0.241540
N	-1.523964	-0.261002	-0.782208
C	1.097585	-0.027338	-2.182093
C	-0.172881	-0.190847	-2.767414
C	-1.416034	-0.186263	-2.110728
C	-0.034754	-0.922714	2.319232
C	-0.133054	0.484155	2.108876
H	-0.203664	-0.172338	-3.851095
H	-1.095879	0.983579	2.163054
C	2.258698	0.197789	-3.106120
H	1.928551	0.580163	-4.073125
H	2.994284	0.884380	-2.680505
H	2.779870	-0.751315	-3.281160
C	-2.655378	-0.135696	-2.956257
H	-3.028717	-1.156901	-3.104381
H	-3.460895	0.425711	-2.477636
H	-2.455235	0.291532	-3.940259
C	-2.756373	0.037092	-0.123420
C	-3.666002	-0.987554	0.189863
C	-2.988969	1.374603	0.267043
C	-4.816099	-0.650488	0.911476
C	-4.157492	1.661033	0.973098
C	-5.064833	0.657803	1.299916
H	-5.528164	-1.433730	1.165109
H	-4.357647	2.685365	1.277973
H	-5.965613	0.899430	1.857377
C	2.602733	0.148794	-0.312101
C	3.547477	-0.895458	-0.263799
C	2.878927	1.415054	0.250353
C	4.767942	-0.653319	0.374867
C	4.116703	1.605495	0.866940
C	5.054673	0.581127	0.937003
H	5.502008	-1.454999	0.428731
H	4.348992	2.570951	1.307271
H	6.008428	0.749142	1.429622
C	1.864163	2.539980	0.170499
H	0.878110	2.074893	0.290550
C	3.295725	-2.265839	-0.864398
H	2.348787	-2.233626	-1.418888
C	-3.448435	-2.424146	-0.242182
H	-2.551369	-2.455574	-0.874589
C	-2.005702	2.468598	-0.102940
H	-1.027184	1.984875	-0.221588
C	-1.863554	3.542756	0.969368
H	-2.781146	4.129499	1.089787
H	-1.604168	3.119929	1.945080
H	-1.073441	4.247530	0.688720
C	-2.367704	3.115856	-1.441467
H	-3.371746	3.554981	-1.405698
H	-1.662866	3.917555	-1.686643
H	-2.346817	2.398883	-2.266578
C	-4.626122	-2.942000	-1.067375
H	-5.539611	-3.001687	-0.465259
H	-4.842768	-2.292977	-1.921975
H	-4.421468	-3.947845	-1.447450
C	-3.193173	-3.334522	0.957690
H	-2.298369	-3.025883	1.508003
H	-4.041258	-3.318503	1.652101
H	-3.043800	-4.370406	0.636409
C	3.145051	-3.326156	0.225420
H	4.042585	-3.374711	0.853285
H	2.288666	-3.107038	0.871718
H	2.988412	-4.316746	-0.214048
C	4.402829	-2.662607	-1.841325
H	4.570336	-1.899774	-2.608744
H	5.356267	-2.814300	-1.323550
H	4.155573	-3.602586	-2.344459
C	1.870773	3.234447	-1.192481
H	1.179026	4.084522	-1.188611
H	2.867047	3.619522	-1.440281
H	1.554351	2.564927	-1.997672
C	2.026770	3.569698	1.282683
H	1.184577	4.267904	1.278727
H	2.068267	3.101972	2.271693
H	2.936367	4.168562	1.158619
H	0.762070	1.093091	2.210063
H	-0.958750	-1.422583	2.626796
C	1.199244	-1.496147	2.987856
H	1.239962	-2.581319	2.836619
H	2.103831	-1.087225	2.513612
C	1.219863	-1.169690	4.476749
H	2.109887	-1.577594	4.964912
H	1.207278	-0.087629	4.636899
H	0.342080	-1.584561	4.983771

2d’
SCF (wB97X) = -1398.45868315
H(0 K)= -1397.709755
H(298 K)= -1397.668056
G(298 K)= -1397.781312
Lowest Frequency = 18.7719cm-1
PCM (Benzene) Energy = -1398.46154519

N	1.316724	0.005871	-0.884792
Al	-0.040235	-0.286834	0.439150
N	-1.506623	-0.206087	-0.784837
C	1.107376	-0.003896	-2.206318
C	-0.165401	-0.153184	-2.780297
C	-1.400141	-0.192462	-2.117174
C	0.063264	-1.131509	2.190637

C	-0.079763	0.446640	2.219375
H	-0.202348	-0.160911	-3.863505
H	-0.957715	0.818076	2.754162
C	2.270633	0.166718	-3.140180
H	1.932522	0.458527	-4.135352
H	2.975361	0.914440	-2.766789
H	2.834097	-0.767825	-3.238848
C	-2.648325	-0.208161	-2.946740
H	-3.146965	-1.180816	-2.860562
H	-3.370477	0.534429	-2.595380
H	-2.432102	-0.026347	-3.999890
C	-2.770133	0.040729	-0.149968
C	-3.643469	-1.022013	0.138104
C	-3.053827	1.363922	0.250640
C	-4.811621	-0.737766	0.851005
C	-4.240155	1.595504	0.948684
C	-5.110677	0.556114	1.255357
H	-5.495028	-1.549858	1.090843
H	-4.483368	2.610039	1.257044
H	-6.024411	0.756645	1.807917
C	2.640980	0.173558	-0.357353
C	3.533585	-0.915829	-0.359508
C	2.974585	1.408489	0.241318
C	4.775303	-0.745948	0.258591
C	4.230443	1.522942	0.841014
C	5.123189	0.457738	0.854570
H	5.474539	-1.578449	0.280762
H	4.512247	2.459910	1.312075
H	6.091802	0.567442	1.334363
C	2.007443	2.579653	0.212429
H	1.004095	2.175037	0.409317
C	3.170015	-2.264325	-0.954351
H	2.313912	-2.128215	-1.624682
C	-3.352535	-2.445887	-0.293339
H	-2.481662	-2.424323	-0.960359
C	-2.149296	2.529573	-0.111590
H	-1.194421	2.125236	-0.472337
C	-1.830576	3.426813	1.079887
H	-2.732543	3.881697	1.503890
H	-1.322224	2.864935	1.868370
H	-1.174277	4.247087	0.768950
C	-2.761287	3.343861	-1.253181
H	-3.724060	3.775113	-0.955496
H	-2.101373	4.169478	-1.539912
H	-2.936393	2.733328	-2.145087
C	-4.523299	-3.049399	-1.068068
H	-5.403745	-3.174477	-0.428412
H	-4.824391	-2.422027	-1.913769
H	-4.262364	-4.039219	-1.455413
C	-2.990128	-3.325777	0.902172
H	-2.096488	-2.956458	1.413284
H	-3.808784	-3.351537	1.630424
H	-2.796904	-4.355204	0.583042
C	2.717533	-3.235030	0.135413
H	3.498297	-3.368080	0.893123
H	1.820687	-2.865370	0.642366
H	2.489812	-4.217923	-0.290621
C	4.307576	-2.860503	-1.780054
H	4.681085	-2.158621	-2.533481
H	5.157759	-3.148190	-1.152728
H	3.972118	-3.765461	-2.295707
C	1.970340	3.251530	-1.161240
H	1.297337	4.116165	-1.146699
H	2.964149	3.610928	-1.453568
H	1.611943	2.575117	-1.942772
C	2.278679	3.613323	1.297796
H	1.472791	4.352667	1.320805
H	2.338953	3.154483	2.289208
H	3.211118	4.162156	1.121567
H	0.820934	0.929386	2.616967
H	-0.867674	-1.594589	2.542489
C	1.249818	-1.685833	2.954627
H	1.347805	-2.766563	2.779279
H	2.182889	-1.243381	2.567308
C	1.160526	-1.425997	4.453053
H	2.040934	-1.789675	4.993886
H	1.061845	-0.353817	4.654371
H	0.279094	-1.916164	4.882147

Allylbenzene

SCF (wB97X) = -348.914321283
H(0 K)= -348.752606
H(298 K)= -348.744458
G(298 K)= -348.786435
Lowest Frequency = 32.5645cm-1
PCM (Benzene) Energy = -348.915477267

C	-0.834515	1.922636	-0.809547
H	-0.106713	1.229423	-1.226574
H	-1.859983	1.815775	-1.149058
C	-0.478723	2.848676	0.078825
H	-1.232848	3.534544	0.467862
C	0.912010	3.053693	0.590245
H	0.939936	2.871722	1.673396
H	1.572081	2.297790	0.142150
C	1.458797	4.435936	0.320730
C	2.176916	5.127198	1.299287
C	1.271230	5.044623	-0.924089
C	2.702858	6.390303	1.041768
H	2.323671	4.666579	2.274699
C	1.794297	6.307085	-1.185083
H	0.703795	4.517815	-1.688770
C	2.512889	6.984658	-0.202682
H	3.257736	6.912607	1.816580
H	1.638637	6.765190	-2.158278
H	2.919284	7.971669	-0.405024

Int-1f
SCF (wB97X) = -1590.16983498
H(0 K)= -1589.369532
H(298 K)= -1589.323329
G(298 K)= -1589.448384
Lowest Frequency = 20.8524cm-1
PCM (Benzene) Energy = -1590.17333366

N	0.258082	1.171720	-1.216053
Al	-0.431272	-0.438170	-0.199356
N	-2.307593	0.223080	-0.600098
C	-0.412298	2.016168	-2.009795
C	-1.802725	1.976417	-2.162774
C	-2.705358	1.176697	-1.449738
C	1.690178	-0.827598	3.160860
C	1.743600	-0.408753	4.424164
H	-2.233386	2.698430	-2.847475
H	0.925734	-0.574701	5.119259
H	0.791338	-1.336290	2.805149
C	0.336886	3.087098	-2.748922
H	-0.301490	3.590179	-3.476755
H	0.721512	3.840871	-2.052433
H	1.211870	2.675521	-3.261600
C	-4.171766	1.432425	-1.657372
H	-4.652105	0.580191	-2.150588
H	-4.689574	1.559510	-0.701466
H	-4.337023	2.319579	-2.270462
C	-3.299550	-0.458504	0.178265
C	-4.049589	-1.510927	-0.379508
C	-3.443828	-0.096055	1.535052
C	-4.983956	-2.159833	0.431115
C	-4.397043	-0.769495	2.301213
C	-5.169491	-1.787643	1.755554
H	-5.568406	-2.977902	0.015905
H	-4.528203	-0.499135	3.345559
H	-5.904326	-2.301764	2.368839
C	1.652410	1.418982	-0.988551
C	2.621944	0.631794	-1.638897
C	2.025071	2.393696	-0.037756
C	3.966935	0.836398	-1.316731
C	3.381444	2.567543	0.240873
C	4.350067	1.794984	-0.389443
H	4.723206	0.223514	-1.804137
H	3.680750	3.306691	0.980771
H	5.401294	1.937032	-0.152601
C	0.989376	3.167255	0.756503
H	0.026702	3.099043	0.233567
C	2.262379	-0.406805	-2.682098
H	1.168968	-0.426396	-2.774443
C	-3.809935	-1.997806	-1.795252
H	-3.251798	-1.222069	-2.333383
C	-2.593762	1.005105	2.140828
H	-1.610902	0.960671	1.646105
C	-2.338736	0.821387	3.630907
H	-3.243007	0.986053	4.227435
H	-1.965919	-0.183379	3.853399
H	-1.589906	1.540609	3.977714
C	-3.169923	2.390911	1.850950
H	-4.182834	2.486103	2.259431
H	-2.551563	3.172772	2.306189
H	-3.219116	2.595219	0.776893
C	-5.103821	-2.254798	-2.562793
H	-5.666338	-3.094047	-2.140046
H	-5.766527	-1.382774	-2.556262
H	-4.888826	-2.507344	-3.605583
C	-2.934961	-3.251840	-1.772930
H	-1.982837	-3.064852	-1.263337
H	-3.438871	-4.065513	-1.239103
H	-2.717462	-3.599628	-2.788265
C	2.722420	-1.801581	-2.265063
H	3.813074	-1.852951	-2.171495
H	2.300661	-2.090531	-1.296877
H	2.419401	-2.547713	-3.007151
C	2.835904	-0.031688	-4.048653
H	2.495676	0.954653	-4.381007
H	3.931304	-0.008510	-4.022929
H	2.540977	-0.762602	-4.808329
C	1.326312	4.647747	0.903869
H	0.508788	5.177954	1.402316
H	2.225043	4.803937	1.509960
H	1.497186	5.127432	-0.065429
C	0.812571	2.509540	2.125387
H	0.036248	3.011371	2.714618
H	0.538964	1.450481	2.032191
H	1.746772	2.541753	2.698994
C	2.759463	-0.602264	2.141541
H	3.568193	0.001547	2.573923
H	2.333836	0.014551	1.330077
H	2.613745	0.118589	4.811574
C	3.337514	-1.840163	1.499433
C	2.592543	-3.012097	1.335097
C	4.643836	-1.810744	1.000456
C	3.138582	-4.117948	0.686924
H	1.572890	-3.059608	1.707465
C	5.192074	-2.913128	0.352783
H	5.233082	-0.902972	1.121377
C	4.438669	-4.074057	0.191859
H	2.541103	-5.018138	0.568309
H	6.211121	-2.867112	-0.023730
H	4.863482	-4.938247	-0.311586

TS-1f exo
SCF (wB97X) = -1590.14822913
H(0 K)= -1589.347592
H(298 K)= -1589.303268
G(298 K)= -1589.421367
Lowest Frequency = -308.5524cm-1
PCM (Benzene) Energy = -1590.15126332

N	-0.609566	-1.213299	-0.821290
Al	0.487322	0.383473	-1.108798
N	2.120734	-0.475474	-0.484370

C	-0.056665	-2.337368	-1.318162
C	1.326765	-2.520985	-1.454187
C	2.349199	-1.720411	-0.900510
C	0.088332	2.305709	-0.636041
C	0.126552	1.830585	0.724434
H	1.641033	-3.467014	-1.880314
H	1.062962	1.988333	1.256994
H	0.909861	2.935631	-0.977305
C	-0.949533	-3.457355	-1.772568
H	-0.366356	-4.309564	-2.123292
H	-1.629416	-3.796628	-0.987196
H	-1.587540	-3.106460	-2.592164
C	3.725687	-2.306575	-0.801324
H	4.187848	-2.085560	0.164715
H	3.707716	-3.386985	-0.948974
H	4.380857	-1.868302	-1.563654
C	3.162530	0.266447	0.160083
C	4.132791	0.930975	-0.613267
C	3.158550	0.350361	1.569102
C	5.110355	1.675027	0.055326
C	4.162654	1.096828	2.186574
C	5.134644	1.754374	1.439688
H	5.863454	2.200906	-0.527959
H	4.178703	1.172424	3.270451
H	5.905567	2.334177	1.939629
C	-1.983714	-1.298355	-0.418621
C	-3.013845	-0.876665	-1.278679
C	-2.274835	-1.865955	0.842855
C	-4.338203	-1.085697	-0.880958
C	-3.613101	-2.072952	1.184012
C	-4.641878	-1.699924	0.325290
H	-5.141978	-0.760564	-1.539029
H	-3.852838	-2.524200	2.144011
H	-5.677361	-1.868534	0.608495
C	-1.171950	-2.242220	1.817627
H	-0.281467	-1.669350	1.527957
C	-2.740615	-0.193041	-2.602428
H	-1.654312	-0.193224	-2.771293
C	4.137777	0.891780	-2.129901
H	3.421594	0.127793	-2.458671
C	2.086595	-0.349436	2.380396
H	1.169819	-0.297483	1.777985
C	1.794484	0.341784	3.706832
H	2.612701	0.220121	4.425982
H	1.622597	1.415100	3.575067
H	0.898059	-0.087094	4.166113
C	2.405118	-1.827243	2.609762
H	3.364753	-1.952589	3.125128
H	1.631553	-2.292126	3.232696
H	2.445932	-2.390775	1.672153
C	5.510546	0.522577	-2.690596
H	6.250308	1.305043	-2.489717
H	5.897679	-0.406234	-2.258656
H	5.462168	0.396692	-3.776525
C	3.670083	2.226274	-2.710366
H	2.650484	2.464276	-2.391751
H	4.325487	3.043978	-2.388930
H	3.677976	2.199634	-3.804819
C	-3.202708	1.263653	-2.553054
H	-4.292759	1.326693	-2.452708
H	-2.776426	1.794990	-1.696320
H	-2.917617	1.794605	-3.466998
C	-3.391989	-0.922140	-3.775748
H	-3.074708	-1.968135	-3.839265
H	-4.484595	-0.916992	-3.692349
H	-3.135471	-0.436280	-4.722175
C	-0.802493	-3.726149	1.750069
H	-0.092239	-3.980288	2.544287
H	-1.688711	-4.357943	1.884003
H	-0.333477	-3.996345	0.800095
C	-1.522838	-1.866278	3.256123
H	-0.651376	-2.005305	3.905588
H	-1.846531	-0.823753	3.341100
H	-2.321858	-2.497561	3.660316
H	-0.860768	2.638328	-1.058402
C	-1.100998	1.581694	1.566689
H	-0.845498	1.850499	2.603890
H	-1.371971	0.514363	1.630994
C	-2.352840	2.327077	1.173269
C	-2.325471	3.689270	0.850057
C	-3.580418	1.661812	1.125094
C	-3.489356	4.361224	0.490925
H	-1.372819	4.215241	0.875214
C	-4.748815	2.329857	0.766915
H	-3.611375	0.599997	1.364129
C	-4.707292	3.683260	0.444882
H	-3.447179	5.418778	0.242219
H	-5.690088	1.785396	0.729383
H	-5.614351	4.207658	0.156300

TS-1f endo

SCF (wB97X) = -1590.14876528
H(0 K)= -1589.348848
H(298 K)= -1589.303849
G(298 K)= -1589.425761
Lowest Frequency = -248.5540cm-1
PCM (Benzene) Energy = -1590.15216592

N	-0.463767	-1.796830	-0.171235
Al	0.352193	-0.212185	-0.996555
N	2.122665	-0.645074	-0.293916
C	0.219946	-2.942497	-0.189380
C	1.616621	-2.994163	-0.368867
C	2.520808	-1.920535	-0.304888
C	-0.505748	1.671393	-0.740063
C	-0.252429	1.432140	0.638617
H	2.052674	-3.985838	-0.418609
H	0.629699	1.852109	1.112932
H	0.155153	2.386311	-1.238294
C	-0.500666	-4.252047	-0.051114
H	0.131238	-5.003481	0.426129
H	-1.432063	-4.155620	0.510651

H	-0.762647	-4.629859	-1.047117
C	3.987915	-2.238147	-0.287832
H	4.391102	-2.127014	-1.302362
H	4.552025	-1.552128	0.348215
H	4.174719	-3.263162	0.036280
C	3.025398	0.403007	0.065730
C	3.747797	1.095504	-0.921034
C	3.108715	0.762760	1.429294
C	4.559110	2.161822	-0.518598
C	3.942440	1.824507	1.780604
C	4.661522	2.524359	0.816421
H	5.121539	2.710367	-1.271952
H	4.024523	2.113796	2.825366
H	5.299747	3.353509	1.109191
C	-1.875112	-1.768286	0.065332
C	-2.768992	-1.988344	-1.001380
C	-2.340697	-1.408624	1.350158
C	-4.136099	-1.808242	-0.766331
C	-3.715864	-1.249806	1.531718
C	-4.609993	-1.435404	0.482107
H	-4.835431	-1.961527	-1.586038
H	-4.094386	-0.968169	2.510394
H	-5.675147	-1.293910	0.642675
C	-1.373136	-1.229622	2.504339
H	-0.471629	-0.767799	2.084318
C	-2.315625	-2.407091	-2.387116
H	-1.230437	-2.569589	-2.363628
C	3.682599	0.717818	-2.387517
H	3.081374	-0.196835	-2.475592
C	2.326580	-0.004015	2.478393
H	1.449089	-0.422639	1.968381
C	1.823435	0.875324	3.617565
H	2.643944	1.269283	4.227787
H	1.236706	1.725021	3.254671
H	1.185813	0.289551	4.288106
C	3.133407	-1.174868	3.043217
H	4.063228	-0.824551	3.506839
H	2.557917	-1.704104	3.810452
H	3.398201	-1.904714	2.273563
C	5.072149	0.425823	-2.952631
H	5.697893	1.325186	-2.958158
H	5.600165	-0.333136	-2.366343
H	5.004621	0.071121	-3.985891
C	2.986834	1.802241	-3.208506
H	1.959674	1.963697	-2.865259
H	3.522127	2.756040	-3.135226
H	2.943144	1.523425	-4.266307
C	-2.585769	-1.310553	-3.415872
H	-3.651946	-1.058189	-3.454148
H	-2.028719	-0.399534	-3.173047
H	-2.281348	-1.632960	-4.416945
C	-2.981631	-3.715588	-2.814382
H	-2.834811	-4.511954	-2.077640
H	-4.062218	-3.588116	-2.942472
H	-2.579924	-4.059703	-3.772630
C	-0.950875	-2.569300	3.109559
H	-0.292766	-2.408161	3.970809
H	-1.818824	-3.141927	3.457110
H	-0.401967	-3.187352	2.393225
C	-1.903079	-0.304322	3.593526
H	-1.119606	-0.095875	4.327913
H	-2.243504	0.655177	3.189119
H	-2.740045	-0.751964	4.141500
C	-1.946718	1.746193	-1.234558
H	-2.498166	0.850011	-0.920139
H	-1.957850	1.754381	-2.330770
H	-1.073614	1.124087	1.279322
C	-2.646041	2.962556	-0.685897
C	-2.475977	4.216772	-1.277994
C	-3.442128	2.866990	0.459281
C	-3.086357	5.347340	-0.742738
H	-1.857469	4.303113	-2.170171
C	-4.053080	3.994967	0.999318
H	-3.586972	1.889594	0.919719
C	-3.876162	5.239622	0.399587
H	-2.948027	6.314311	-1.219501
H	-4.671575	3.902419	1.888443
H	-4.354599	6.120932	0.817982

2f

SCF (wB97X) = -1590.17804135
H(0 K)= -1589.376533
H(298 K)= -1589.331693
G(298 K)= -1589.452796
Lowest Frequency = 18.7694cm-1
PCM (Benzene) Energy = -1590.18185456

N	-0.461612	-1.795044	-0.078164
Al	0.310325	-0.049375	-0.281301
N	2.121952	-0.652506	-0.290532
C	0.216358	-2.944893	-0.160675
C	1.606807	-3.002415	-0.352794
C	2.510098	-1.931479	-0.355904
C	-0.577125	1.550188	-0.976202
C	-0.263475	1.571564	0.574320
H	2.037774	-3.994407	-0.423906
H	0.445930	2.354642	0.855989
H	-0.017472	2.326742	-1.508693
C	-0.517091	-4.248707	-0.032440
H	0.166412	-5.054405	0.239085
H	-1.314097	-4.187196	0.713137
H	-0.996582	-4.523901	-0.978479
C	3.975897	-2.239773	-0.408382
H	4.420861	-1.824212	-1.319834
H	4.508844	-1.773635	0.426017
H	4.160303	-3.314192	-0.390038
C	3.073554	0.370616	0.042835
C	3.770770	1.052149	-0.968628
C	3.212900	0.716605	1.403633
C	4.612986	2.102617	-0.593618
C	4.075891	1.765425	1.725784
C	4.766931	2.459159	0.739390

H	5.154116	2.648053	-1.363844
H	4.203978	2.042865	2.770020
H	5.426722	3.278400	1.011256
C	-1.885937	-1.795156	0.097276
C	-2.719161	-2.079677	-1.002642
C	-2.411031	-1.392072	1.344864
C	-4.099116	-1.943077	-0.831024
C	-3.798205	-1.275636	1.459947
C	-4.636912	-1.541874	0.383743
H	-4.759582	-2.140221	-1.672035
H	-4.229041	-0.961174	2.406097
H	-5.712241	-1.432904	0.493448
C	-1.499714	-1.118651	2.528760
H	-0.623375	-0.576495	2.146248
C	-2.168872	-2.453990	-2.367334
H	-1.129096	-2.777427	-2.247491
C	3.613725	0.690171	-2.431936
H	3.082371	-0.268564	-2.484199
C	2.501087	-0.040785	2.511660
H	1.749338	-0.697527	2.054645
C	1.758469	0.882912	3.472416
H	2.440081	1.564790	3.992863
H	1.009744	1.479028	2.943165
H	1.244698	0.293110	4.239809
C	3.484468	-0.932959	3.271280
H	4.266403	-0.334888	3.753231
H	2.970272	-1.500230	4.054314
H	3.980568	-1.652419	2.611580
C	4.962639	0.515650	-3.126531
H	5.511088	1.461992	-3.184185
H	5.604750	-0.200438	-2.602786
H	4.825451	0.160590	-4.152562
C	2.758855	1.725616	-3.161701
H	1.758411	1.796433	-2.723911
H	3.220175	2.718343	-3.108541
H	2.647886	1.465258	-4.219457
C	-2.140423	-1.239446	-3.294645
H	-3.141355	-0.807368	-3.407645
H	-1.482267	-0.458288	-2.899854
H	-1.779568	-1.520441	-4.289720
C	-2.936452	-3.610058	-3.004326
H	-3.011604	-4.472659	-2.333563
H	-3.956328	-3.319509	-3.277137
H	-2.441852	-3.936997	-3.924000
C	-0.993110	-2.415380	3.161844
H	-0.358188	-2.195916	4.027662
H	-1.824578	-3.039832	3.509456
H	-0.395233	-3.007931	2.462527
C	-2.134064	-0.222348	3.584364
H	-1.388814	0.057868	4.334554
H	-2.529320	0.700789	3.148181
H	-2.951107	-0.723379	4.116435
C	-2.062172	1.631867	-1.324670
H	-2.599142	0.746038	-0.952409
H	-2.182388	1.617336	-2.416417
H	-1.160449	1.662931	1.195914
C	-2.728683	2.856690	-0.757708
C	-2.485980	4.124336	-1.297246
C	-3.564141	2.764725	0.359630
C	-3.063252	5.261938	-0.743732
H	-1.831856	4.212900	-2.163760
C	-4.141520	3.900967	0.922447
H	-3.766946	1.779475	0.781094
C	-3.893378	5.154666	0.371529
H	-2.867763	6.237119	-1.183168
H	-4.788167	3.806466	1.791610
H	-4.344116	6.043182	0.805568

Int-3

SCF (wB97X) = -1590.16932038
H(0 K)= -1589.368576
H(298 K)= -1589.322597
G(298 K)= -1589.447497
Lowest Frequency = 17.7132cm-1
PCM (Benzene) Energy = -1590.17297969

Al	-0.495481	-0.064738	-0.536039
N	0.130642	1.670411	0.297477
N	-2.389922	0.595400	-0.267503
C	-1.944340	2.884263	0.314452
H	-2.399737	3.849285	0.505968
C	-2.817444	1.826413	0.033723
C	-3.997677	-0.732360	-1.584186
C	-0.558647	2.801209	0.497224
C	2.472363	2.202309	-0.253306
C	-3.359028	-0.458518	-0.359612
C	1.524319	1.654041	0.632520
C	-4.291714	2.099024	0.107519
H	-4.764287	1.469933	0.869158
H	-4.785376	1.847585	-0.837467
H	-4.494819	3.144662	0.342515
C	1.923953	1.011285	1.827017
C	3.287963	0.972758	2.128109
H	3.620526	0.481134	3.037099
C	-3.593928	-1.254566	0.779093
C	0.150934	4.035992	0.976073
H	0.736979	3.826091	1.876718
H	-0.554000	4.839729	1.193298
H	0.864079	4.396406	0.226958
C	-4.869663	-1.821114	-1.647607
H	-5.362579	-2.049668	-2.590201
C	1.379923	-3.733008	1.800205
H	1.134832	-3.422183	2.812255
H	1.296372	-4.795768	1.582424
C	-4.476943	-2.331118	0.665776
H	-4.661636	-2.956759	1.537039
C	-2.910196	-0.987890	2.107496
H	-2.337227	-0.055342	2.015694
C	4.231394	1.541583	1.281184
H	5.287127	1.493924	1.533190
C	3.822613	2.143714	0.100169
H	4.565404	2.563116	-0.575632

C	1.752788	-2.859766	0.865900
H	1.825011	-1.797067	1.115672
C	2.076900	2.769640	-1.602863
H	0.996286	2.957035	-1.593920
C	-3.726656	0.091947	-2.826866
H	-3.206928	1.007289	-2.517258
C	-5.009009	0.506917	-3.543939
H	-5.540550	-0.358010	-3.955181
H	-4.783094	1.172971	-4.382351
H	-5.702900	1.028475	-2.876267
C	0.902854	0.373375	2.756050
H	0.202345	-0.195240	2.118014
C	-5.111435	-2.617645	-0.535364
H	-5.791518	-3.461927	-0.606303
C	1.516970	-0.614094	3.741467
H	2.162833	-1.348968	3.251731
H	0.726567	-1.157866	4.268379
H	2.113499	-0.100603	4.504289
C	0.079223	1.407412	3.527049
H	0.733229	2.052999	4.125000
H	-0.609032	0.907001	4.217616
H	-0.521417	2.043801	2.872933
C	-1.920974	-2.103201	2.443633
H	-2.430741	-3.068809	2.540930
H	-1.410509	-1.900213	3.392902
H	-1.154664	-2.214851	1.666482
C	-3.919098	-0.798139	3.238437
H	-4.631413	0.003930	3.020699
H	-3.409016	-0.549674	4.174813
H	-4.498126	-1.711083	3.416476
C	2.773362	4.090264	-1.920058
H	2.631310	4.833038	-1.127699
H	2.386713	4.513725	-2.851944
H	3.852380	3.958259	-2.052956
C	2.352795	1.736071	-2.696162
H	3.421620	1.496034	-2.743617
H	2.047167	2.109235	-3.679260
H	1.818638	0.797707	-2.503728
C	-2.797616	-0.666191	-3.775070
H	-1.853909	-0.929136	-3.283789
H	-2.566450	-0.068692	-4.663152
H	-3.265075	-1.599107	-4.110297
C	2.082388	-3.200726	-0.553442
H	1.316594	-2.734796	-1.193325
C	3.439132	-2.704159	-0.983778
C	4.423353	-3.592883	-1.421516
C	3.739039	-1.337211	-0.953816
C	5.673634	-3.134889	-1.829419
H	4.201429	-4.658300	-1.444205
C	4.987987	-0.877541	-1.359381
H	2.981771	-0.627039	-0.615008
C	5.960180	-1.773328	-1.799938
H	6.424580	-3.843606	-2.169034
H	5.200789	0.188665	-1.326655
H	6.934621	-1.411473	-2.116806
H	2.010319	-4.284164	-0.704147

TS-3 trans
SCF (wB97X) = -1590.15132682
H(0 K)= -1589.354982
H(298 K)= -1589.310566
G(298 K)= -1589.428435
Lowest Frequency = -915.0560cm-1
PCM (Benzene) Energy = -1590.15463501

Al	0.415674	-0.675409	0.225832
N	-0.421002	0.965857	0.992757
N	2.211657	-0.021732	0.749916
C	1.435398	1.440002	2.476412
H	1.724022	2.000928	3.357902
C	2.449258	0.713554	1.839258
C	3.865453	-1.760016	0.171381
C	0.122474	1.639649	2.017726
C	-2.873840	0.872283	1.313887
C	3.304862	-0.489275	-0.045418
C	-1.753020	1.329525	0.592270
C	3.833829	0.763271	2.416349
H	3.920670	1.525130	3.192390
H	4.589843	0.947915	1.648342
H	4.080737	-0.208707	2.860615
C	-1.904315	2.162274	-0.539677
C	-3.189518	2.576052	-0.894034
H	-3.321088	3.226883	-1.754191
C	3.735585	0.329913	-1.113194
C	-0.688438	2.681594	2.734399
H	-0.069589	3.254090	3.426098
H	-1.501629	2.218963	3.303089
H	-1.164390	3.370044	2.029611
C	4.888280	-2.186658	-0.681073
H	5.328512	-3.169890	-0.526176
C	0.432302	-0.751535	-1.987099
H	0.890826	0.129328	-2.442532
H	1.070178	-1.631998	-2.116071
C	4.767996	-0.136065	-1.927987
H	5.117474	0.482425	-2.750861
C	3.096716	1.684121	-1.363493
H	2.066093	1.626180	-0.986768
C	-4.302669	2.166385	-0.166754
H	-5.295972	2.499209	-0.456057
C	-4.140240	1.309516	0.910928
H	-5.014776	0.956064	1.453319
C	-0.960591	-0.937271	-2.177494
H	-1.575106	-0.055871	-2.368498
C	-2.768019	-0.081395	2.489703
H	-1.711005	-0.158395	2.776099
C	3.376796	-2.683406	1.268663
H	2.662880	-2.124903	1.888356
C	4.516482	-3.153501	2.170582
H	5.231313	-3.775029	1.620063
H	4.132429	-3.757600	2.998390
H	5.076511	-2.313804	2.594916
C	-0.695229	2.608805	-1.339873
H	0.017109	1.775053	-1.322289

C	5.345443	-1.383418	-1.715661
H	6.143967	-1.731107	-2.365175
C	-1.014563	2.900976	-2.801628
H	-1.575630	2.086139	-3.270350
H	-0.089160	3.039539	-3.369446
H	-1.600868	3.819416	-2.917971
C	0.011349	3.807383	-0.703892
H	-0.674092	4.655971	-0.594462
H	0.847758	4.135776	-1.332206
H	0.420881	3.569734	0.282889
C	3.030590	2.050588	-2.842529
H	4.021982	2.265566	-3.256535
H	2.429116	2.955920	-2.979122
H	2.585113	1.252974	-3.445673
C	3.796663	2.798657	-0.582551
H	3.704804	2.666390	0.498945
H	3.362761	3.773909	-0.828421
H	4.864158	2.837005	-0.829207
C	-3.552527	0.409345	3.707253
H	-3.297125	1.435924	3.990227
H	-3.360797	-0.235170	4.570474
H	-4.631856	0.384617	3.522683
C	-3.245239	-1.481141	2.098587
H	-4.283951	-1.457951	1.747903
H	-3.193537	-2.159068	2.957243
H	-2.638409	-1.903740	1.293734
C	2.634769	-3.880250	0.673121
H	1.784308	-3.559278	0.060832
H	2.250926	-4.536026	1.461307
H	3.300885	-4.474558	0.036944
C	-1.569087	-2.048074	-1.521568
H	-1.061375	-1.884124	-0.302987
C	-3.041171	-2.213029	-1.457739
C	-3.599541	-3.490839	-1.323741
C	-3.906744	-1.112035	-1.439162
C	-4.972770	-3.666361	-1.187762
H	-2.939078	-4.356369	-1.324720
C	-5.279773	-1.284374	-1.308551
H	-3.498688	-0.105995	-1.509204
C	-5.822090	-2.562304	-1.181489
H	-5.381335	-4.668680	-1.087272
H	-5.928344	-0.411015	-1.294117
H	-6.895029	-2.695789	-1.073674
H	-1.049935	-3.002236	-1.671828

TS-3 cis

SCF (wB97X) = -1590.14605457
H(0 K)= -1589.349626
H(298 K)= -1589.305197
G(298 K)= -1589.423519
Lowest Frequency = -1033.2191cm-1
PCM (Benzene) Energy = -1590.14969015

Al	0.138313	0.320936	0.357911
N	1.931991	-0.422199	0.743602
N	-0.685027	-1.435255	0.766298
C	1.160183	-2.289829	2.047782
H	1.460968	-3.055540	2.753896
C	-0.166610	-2.348649	1.592023
C	-3.110024	-1.155049	1.112254
C	2.166814	-1.422624	1.601614
C	3.624466	1.377067	0.654722
C	-2.033404	-1.562059	0.299145
C	3.030088	0.232418	0.095633
C	-1.018724	-3.487582	2.070433
H	-1.597580	-3.923987	1.251324
H	-1.748191	-3.134718	2.808181
H	-0.416006	-4.267593	2.537612
C	3.435243	-0.264018	-1.164778
C	4.453941	0.407413	-1.841762
H	4.779923	0.040937	-2.811716
C	-2.239699	-1.980219	-1.035246
C	3.555351	-1.630986	2.133510
H	4.312850	-1.547413	1.349896
H	3.656257	-2.599641	2.625266
H	3.785193	-0.850937	2.869385
C	-4.398785	-1.202995	0.571923
H	-5.238400	-0.881170	1.185111
C	0.075351	0.872258	-1.794256
H	0.737795	0.273233	-2.423897
H	-0.974289	0.616234	-1.986721
C	-3.546917	-2.013739	-1.523333
H	-3.726171	-2.332306	-2.546142
C	-1.067098	-2.382534	-1.910522
H	-0.245624	-1.694656	-1.673856
C	5.051417	1.542091	-1.302677
H	5.838273	2.054438	-1.849214
C	4.635297	2.018280	-0.068369
H	5.099361	2.908849	0.351465
C	0.385829	2.252136	-1.691440
H	1.423286	2.542776	-1.867654
C	3.204157	1.940746	1.996762
H	2.456797	1.263446	2.430396
C	-2.924937	-0.627198	2.522407
H	-1.879053	-0.783402	2.815388
C	-3.811494	-1.362557	3.527388
H	-4.872453	-1.159171	3.345948
H	-3.591058	-1.034772	4.547952
H	-3.679510	-2.448913	3.484359
C	2.798080	-1.508775	-1.756296
H	1.763150	-1.546572	-1.389077
C	-4.621858	-1.632392	-0.727995
H	-5.631444	-1.658295	-1.128941
C	2.751712	-1.495881	-3.280556
H	2.321028	-0.569623	-3.674266
H	2.147293	-2.332800	-3.645568
H	3.748630	-1.611194	-3.720370
C	3.488955	-2.785722	-1.271419
H	4.559408	-2.763739	-1.506828
H	3.058601	-3.666104	-1.760520
H	3.383865	-2.930121	-0.193003
C	-1.351495	-2.248091	-3.401220

H	-2.062244	-3.004539	-3.752936
H	-0.429638	-2.386016	-3.974478
H	-1.757051	-1.263291	-3.652287
C	-0.562911	-3.790307	-1.587901
H	-0.159829	-3.859364	-0.572842
H	0.239927	-4.076648	-2.276664
H	-1.365407	-4.530228	-1.687915
C	4.384582	2.029076	2.963924
H	4.895952	1.068145	3.081161
H	4.052899	2.360203	3.952999
H	5.130311	2.751009	2.612905
C	2.549801	3.311887	1.832993
H	3.257461	4.031942	1.405747
H	2.219358	3.705305	2.799649
H	1.678900	3.265012	1.173062
C	-3.195500	0.876648	2.579292
H	-2.524697	1.431654	1.915053
H	-3.061423	1.256115	3.597663
H	-4.223049	1.101417	2.270095
C	-0.352795	3.069667	-0.789949
H	-0.229752	2.283373	0.298887
H	0.114358	4.040830	-0.597138
C	-1.839567	3.220978	-0.866470
C	-2.372657	4.512034	-0.991408
C	-2.743253	2.149549	-0.804595
C	-3.742696	4.728643	-1.076840
H	-1.687990	5.357553	-1.029253
C	-4.117583	2.363908	-0.886703
H	-2.385919	1.134765	-0.647642
C	-4.625225	3.650981	-1.029857
H	-4.122745	5.742040	-1.178472
H	-4.790040	1.510763	-0.830130
H	-5.697286	3.815261	-1.097272

Trans 4f

SCF (wB97X) = -1590.22546835
H(0 K)= -1589.427911
H(298 K)= -1589.382723
G(298 K)= -1589.504542
Lowest Frequency = 15.4438cm-1
PCM (Benzene) Energy = -1590.22926398

Al	-0.504879	0.595608	0.316511
N	0.284839	-1.025068	1.039199
N	-2.324972	0.048136	0.673962
C	-1.650513	-1.434251	2.434705
H	-1.996634	-1.984932	3.301697
C	-2.622382	-0.695869	1.742483
C	-3.914978	1.837024	0.090126
C	-0.318990	-1.668631	2.047865
C	2.726486	-0.958357	1.397502
C	-3.361227	0.570376	-0.164149
C	1.615360	-1.424601	0.669691
C	-4.038306	-0.748371	2.234766
H	-4.748720	-0.878924	1.414083
H	-4.295779	0.203998	2.714066
H	-4.182784	-1.546516	2.963930
C	1.776174	-2.260846	-0.455591
C	3.064488	-2.685002	-0.786970
H	3.207131	-3.336611	-1.644667
C	-3.740003	-0.185443	-1.294076
C	0.438887	-2.718333	2.806369
H	0.884661	-3.450343	2.125613
H	-0.206136	-3.239424	3.514230
H	1.272724	-2.271557	3.358596
C	-4.884512	2.322336	-0.792295
H	-5.320617	3.302672	-0.610529
C	-0.176072	0.902006	-1.634147
H	-0.605477	0.131064	-2.284428
H	-0.660186	1.856253	-1.883398
C	-4.718306	0.338588	-2.140087
H	-5.029722	-0.231423	-3.011934
C	-3.115274	-1.540662	-1.566210
H	-2.122787	-1.538568	-1.095855
C	4.168165	-2.275648	-0.046375
H	5.164012	-2.613647	-0.320593
C	3.996796	-1.402794	1.017649
H	4.866308	-1.039869	1.561618
C	1.293673	0.990412	-1.752273
H	1.814071	0.044692	-1.923063
C	2.598132	0.045132	2.527240
H	1.537276	0.129787	2.795353
C	-3.463107	2.694637	1.254901
H	-2.798361	2.091236	1.885961
C	-4.637828	3.160113	2.113018
H	-5.299456	3.831950	1.555135
H	-4.282988	3.710200	2.989930
H	-5.248235	2.321263	2.463162
C	0.579966	-2.661452	-1.297947
H	-0.099662	-1.799246	-1.301695
C	-5.291917	1.581034	-1.892399
H	-6.048892	1.974296	-2.565253
C	0.941273	-2.952843	-2.749922
H	1.535998	-2.146837	-3.191960
H	0.032833	-3.066222	-3.349590
H	1.510159	-3.883559	-2.853491
C	-0.194388	-3.834537	-0.695333
H	0.455245	-4.707479	-0.562463
H	-1.016575	-4.131364	-1.356966
H	-0.633230	-3.584227	0.275856
C	-2.919622	-1.824097	-3.051243
H	-3.872688	-1.954167	-3.575574
H	-2.356628	-2.754205	-3.185337
H	-2.370354	-1.020286	-3.551811
C	-3.917343	-2.667171	-0.911436
H	-3.929011	-2.581500	0.179174
H	-3.488240	-3.644414	-1.157272
H	-4.956418	-2.661520	-1.260642
C	3.368001	-0.378206	3.776974
H	3.094152	-1.382714	4.117474
H	3.176381	0.318604	4.598534
H	4.449232	-0.378361	3.602063

C	3.062736	1.424884	2.057458
H	4.115735	1.402131	1.751334
H	2.957420	2.162296	2.859960
H	2.479940	1.773228	1.199790
C	-2.655485	3.893587	0.756697
H	-1.782784	3.579483	0.175572
H	-2.293586	4.496120	1.595750
H	-3.269527	4.539337	0.118313
C	2.041669	2.092813	-1.533792
H	-0.116377	1.830088	1.234291
C	3.493945	2.158661	-1.452618
C	4.116055	3.399603	-1.235925
C	4.319528	1.022015	-1.523559
C	5.495028	3.505399	-1.095833
H	3.494080	4.290486	-1.168562
C	5.695835	1.127067	-1.382727
H	3.874012	0.039766	-1.665017
C	6.295962	2.368478	-1.167291
H	5.945923	4.479870	-0.925976
H	6.307637	0.228725	-1.432397
H	7.373605	2.446049	-1.053278
H	1.520233	3.037623	-1.357995

Cis 4f
SCF (wB97X) = -1590.22341220
H(0 K)= -1589.424519
H(298 K)= -1589.379786
G(298 K)= -1589.499108
Lowest Frequency = 20.2655cm-1
PCM (Benzene) Energy = -1590.22732373

Al	0.204843	0.245107	0.471442
N	2.005873	-0.385228	0.774862
N	-0.588021	-1.508518	0.709268
C	1.284824	-2.331169	1.984453
H	1.604212	-3.115723	2.661080
C	-0.032508	-2.430606	1.500162
C	-3.027962	-1.365054	1.027126
C	2.268336	-1.412322	1.589587
C	3.573324	1.513690	0.696928
C	-1.921325	-1.664188	0.207790
C	3.064781	0.331937	0.132000
C	-0.828638	-3.636440	1.902669
H	-1.437438	-4.009053	1.074338
H	-1.524596	-3.378408	2.708853
H	-0.181827	-4.437806	2.262137
C	3.498270	-0.128319	-1.131114
C	4.480276	0.605982	-1.797234
H	4.831722	0.267867	-2.768926
C	-2.086794	-2.005337	-1.153962
C	3.660659	-1.590443	2.119335
H	4.415404	-1.444862	1.342235
H	3.799030	-2.572589	2.573382
H	3.857001	-0.829178	2.884231
C	-4.305305	-1.444705	0.462809
H	-5.168528	-1.210370	1.083291
C	0.059714	0.957609	-1.399790
H	0.844030	0.490936	-2.009752
H	-0.904565	0.664295	-1.833287
C	-3.383254	-2.067656	-1.666364
H	-3.532086	-2.325452	-2.710844
C	-0.883132	-2.303023	-2.028609
H	-0.094841	-1.598201	-1.735883
C	5.006748	1.769266	-1.245340
H	5.766666	2.330025	-1.782430
C	4.549779	2.217906	-0.014264
H	4.950637	3.136866	0.409194
C	0.242770	2.423216	-1.380323
H	1.277732	2.755700	-1.497016
C	3.072486	2.052899	2.021265
H	2.390200	1.310195	2.454045
C	-2.892663	-0.921185	2.471365
H	-1.838538	-1.004535	2.763587
C	-3.722126	-1.795647	3.412399
H	-4.794622	-1.665977	3.230175
H	-3.537391	-1.523062	4.456011
H	-3.503253	-2.862187	3.296162
C	2.916643	-1.389568	-1.744037
H	1.913557	-1.522473	-1.316045
C	-4.487637	-1.794674	-0.866717
H	-5.488544	-1.843407	-1.286524
C	2.762919	-1.302354	-3.258919
H	2.208798	-0.408870	-3.565017
H	2.223037	-2.178890	-3.633000
H	3.731514	-1.285588	-3.770424
C	3.729311	-2.630411	-1.369334
H	4.771584	-2.527579	-1.692959
H	3.316838	-3.523182	-1.851397
H	3.729917	-2.812702	-0.291199
C	-1.147257	-2.089835	-3.513791
H	-1.828437	-2.844947	-3.922125
H	-0.212522	-2.165861	-4.077416
H	-1.579161	-1.104391	-3.713318
C	-0.330704	-3.707789	-1.780375
H	0.038007	-3.829245	-0.757092
H	0.507703	-3.914620	-2.455297
H	-1.097080	-4.470682	-1.959246
C	4.215330	2.276584	3.009956
H	4.809634	1.369531	3.162125
H	3.828999	2.596127	3.982715
H	4.898988	3.058043	2.659952
C	2.277517	3.342399	1.814327
H	2.912132	4.125398	1.382331
H	1.886916	3.715525	2.766327
H	1.426345	3.193502	1.141353
C	-3.295754	0.545065	2.629871
H	-2.690372	1.204130	2.001686
H	-3.177755	0.866996	3.669533
H	-4.346224	0.693277	2.352243
C	-0.664047	3.408273	-1.187751
H	-0.273330	1.197108	1.644320
H	-0.276441	4.427008	-1.213303

C	-2.106815	3.340004	-0.995150
C	-2.837467	4.540719	-1.099334
C	-2.840220	2.173412	-0.697082
C	-4.215017	4.579462	-0.940822
H	-2.293712	5.458590	-1.315634
C	-4.221149	2.213360	-0.533074
H	-2.338586	1.220352	-0.563108
C	-4.920359	3.410191	-0.660127
H	-4.741878	5.525717	-1.034552
H	-4.748807	1.290972	-0.299428
H	-5.999324	3.434021	-0.534913
